# Pore Extractor 2D: An ImageJ toolkit for quantifying cortical pore morphometry on histological bone images, with application to intraskeletal and regional patterning

**DOI:** 10.1002/ajpa.24618

**Published:** 2022-09-14

**Authors:** Mary E. Cole, Sam D. Stout, Victoria M. Dominguez, Amanda M. Agnew

**Affiliations:** ^1^ Skeletal Biology Research Laboratory The Ohio State University Columbus Ohio USA; ^2^ Department of Anthropology The Ohio State University Columbus Ohio USA; ^3^ Department of Anthropology Lehman College CUNY Bronx New York USA

**Keywords:** bone histology, skeletal biology, cortical porosity, ImageJ, automated image analysis

## Abstract

**Objectives:**

Cortical porosity is used as a proxy of bone quality, fragility, and remodeling activity in anthropological contexts. Histological quantification is limited by time‐intensive manual annotation. Pore Extractor 2D is an ImageJ toolkit developed for computer‐assisted pore identification and automated pore morphometry.

**Materials and Methods:**

Toolkit components include: (1) Utilities for cortical border clearing, (2) *Image Pre‐Processing:* Image contrast enhancement and noise reduction, (3) *Pore Extractor:* User‐directed options for segmenting, closing, and smoothing pore spaces, (4) *Pore Modifier:* Utilities to expedite manual correction of extracted pore spaces, and (5) *Pore Analyzer:* Morphometric analyses by pore type and anatomical region. Pore Extractor 2D was validated against manual annotation in a sample of midshaft mid‐thoracic human ribs (*n* = 30). Intraskeletal and regional analyses were piloted on matched (*n* = 9) human midshaft femora, tibiae, and sixth ribs.

**Results:**

Pore Extractor 2D was statistically consistent with manual annotation of bone area, percent porosity, pore density, and mean pore size. The toolkit significantly (*p* < .05) reduced the smoothing effect of manual annotation by fitting pore borders to pixel brightness variations. Intraskeletal analyses found that the femur and tibia significantly exceeded the rib in “cortical” type porosity, while the rib predominantly formed “trabecularized” type porosity. Regional analyses determined that pore system expansion was elevated in the anterior femur, the anterior and medial tibia, and the cutaneous cortex of the rib.

**Discussion:**

This toolkit provides expedited, semi‐automated porosity quantification that replicates manual annotation. Intraskeletal and regional variation in pore morphometry reflect localized strain patterning.

## Graphical abstract

Regional subdivision of semi‐automatically extracted cortical pores is displayed on a histological cross‐section of a human tibia.

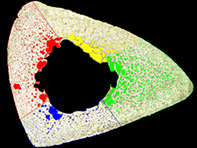

## 1 INTRODUCTION

Cortical pores transmit vascular networks through bone tissue to transport nutrients, cell signaling factors, and bone cell progenitors (Chen et al., 2021; Eriksen, 2010). Cortical pores within primary osteons are created by bone modeling, where forming bone surrounds a blood vessel on the bone surface (Kohara et al., 2015). Cortical pores within secondary osteons are created by bone remodeling, which extends and interconnects existing vascular networks within the bone cortex. Osteoclasts tunnel into and resorb existing bone in a “cutting cone,” and osteoblasts follow behind to form new bone tissue surrounding the central blood vessel. (Eriksen, 2010). Vascular systems, and their surrounding cortical pores, are complex 3D network structures (Maggiano et al., 2016; Stout et al., 1999). Closer to the active cutting cone, cortical pores appear as large, irregular “resorptive bays.” These resorption spaces can coalesce into “trabecularized” pores adjacent to the endosteum (Andreasen et al., 2020; Zebaze & Seeman, 2015).

Quantifying cortical porosity has broad applications in biological anthropology. Cortical porosity is a proxy of mechanical loading history, as it reflects the strain‐based patterning of bone remodeling (Stout et al., 2019). Cortical porosity is also a metric of bone quality and fragility. Porosity accounts for approximately 70% of appendicular age‐associated bone loss (Zebaze et al., 2010) and 76% of the reduction in tensile bone strength with age (McCalden et al., 1993). As early as the third decade of life, bone resorption begins to outpace bone formation due to reduced physical activity, declining sex steroids, and cellular senescence (Demontiero et al., 2012; Infante & Rodríguez, 2018). Aging is consistently associated with increases in percent porosity, pore size, and pore system convergence (Stout et al., 2019). Consequently, cortical pore size and shape are components of some histological age‐at‐death estimation methods (Andronowski & Cole, 2021). Cortical pores also concentrate mechanical stress (Ebacher et al., 2007) and increase the initiation and propagation of microcracks into fracture (Diab & Vashishth, 2005). In vivo cortical porosity has been identified as an independent predictor of fracture in HR‐pQCT visualization of the proximal femur (Ahmed et al., 2015; Kral et al., 2017), distal radius, (Bala et al., 2014, 2015) and distal tibia (Bjørnerem et al., 2013; Sundh et al., 2015).

Despite the 3D complexity of pore systems, there is a strong correlation between 2D histology and 3D micro‐computed tomography in quantifying percent porosity, pore diameter, and pore separation (Particelli et al., 2012; Wachter et al., 2001). Histological quantification of porosity is broadly accessible, lower‐cost than 3D imaging techniques, and can be applied to existing slide collections. However, histological analyses of porosity have been limited by the tedious task of manual annotation. For a single histological cross‐section, cortical pores number in the hundreds for human ribs and the thousands for human long bones. The time required to manually trace and measure each pore on a digital microscopic image necessarily limits the region or sample size for research.

To address the challenges of manual data collection, Pore Extractor 2D was developed to expedite cortical pore identification and to automate analyses of pore morphometry, type, and regional distribution. Pore Extractor 2D is a macro toolkit for the free, open‐source image analysis software ImageJ (NIH) (Schindelin et al., 2012). This study validates Pore Extractor 2D against manual pore identification, using a histological sample of midshaft, mid‐thoracic human ribs (*n* = 30).

The intraskeletal and regional applications of this toolkit are also demonstrated, using a pilot sample of matched (*n* = 9) midshaft femora, tibiae, and sixth ribs. The femur and tibia are both weight‐bearing, dynamically loaded bones. The rib experiences more systemic bone loss, being similarly loaded between individuals by intercostal and other muscles in breathing (Bellemare et al., 2003; Robling & Stout, 2003). Cortical pores and other microstructural products of bone remodeling have a complex relationship with mechanical strain. Bone remodeling can be triggered both by low strain (disuse‐related or stochastic remodeling) and by high strain (targeted remodeling). (Hughes et al., 2020). Consequently, the microstructural products of remodeling do not always clearly reflect intraskeletal strain patterning (Andronowski & Cole, 2021; Stout et al., 2019). Cross‐sections from skeletal elements with varying mechanical strain environments (midshaft femur, midshaft rib, and distal radius) have been reported as intraskeletally consistent in percent porosity (Hunter & Agnew, 2016). Within a cross‐section, however, localized variation in mechanical strain, and the resulting disparity in remodeling activity, has been shown to significantly influence microstructural patterning (Stout et al., 2019). Cortical porosity is elevated in lower‐strain regions, such as adjacent to the endosteum (Atkinson, 1965; Bousson et al., 2001; Jowsey, 1960; Martin et al., 1980; Thomas et al., 2005) and in regions under tension, as opposed to compression (Agnew et al., 2013; Agnew & Stout, 2012; Skedros, Mason, & Bloebaum, 1994).

The objectives of this research were to (1) develop and validate an image processing toolkit for expedited porosity quantification and (2) explore intraskeletal and regional variation in cortical porosity and strain environment to demonstrate the toolkit's utility.

## 2 MATERIALS AND METHODS

### 2.1 Cross‐sectional microscopic image acquisition

Pore Extractor 2D was developed for digital images of transverse cross‐sections acquired with brightfield (light transmission) microscopy. The toolkit has been successfully implemented on histological sections prepared with epoxy resin embedding (Cole & Stout, 2015), methyl methacrylate embedding (Cole et al., 2022), and decalcification followed by cryosectioning (Cole & Stout, 2017, 2018).

The cross‐section should be a complete transverse cut to facilitate geometric analysis, although thin cracks through the cortex can be corrected. Histological slides should be chosen to minimize surface debris, mounting medium crystallization, and taphonomic alteration (e.g., burning and diagenesis), which the computer‐assisted tool *Pore Extractor* may mistake for pore spaces. Slides with surface damage can still be analyzed by skipping *Pore Extractor* and adding pores completely manually in *Pore Modifier*, then proceeding with automated morphometry in *Pore Analyzer*.

The user should acquire overlapping brightfield microscopic images of the entire cross‐section with consistent camera settings, saving in a lossless format (e.g., TIFF). Use of a microscope with a fully automated stage and image stitching software simplifies cross‐sectional image acquisition. For microscopes with manual stages, overlapping images can be automatically merged with the software Microsoft Image Composite Editor or the *Photomerge* tool in Adobe Photoshop.

### 2.2 Pore Extractor 2D installation

**FIGURE 1:**
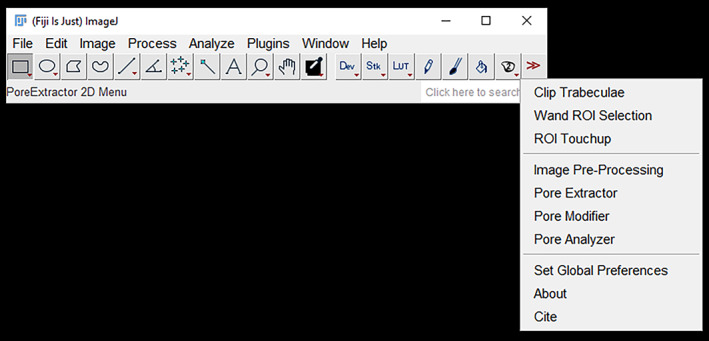
The Pore Extractor 2D button and dropdown menu after toolkit installation in ImageJ

ImageJ (NIH) is a free, open‐source image processing software. The FIJI (“Fiji Is Just ImageJ”) distribution of ImageJ has many plug‐ins pre‐installed (Schindelin et al., 2012). Installation instructions for ImageJ and the Pore Extractor 2D macro toolkit (Figure 1) are available in the supplemental material. File inputs and outputs for each Pore Extractor 2D tool are listed in Table 1.

**TABLE 1:** Pore Extractor 2D macro tool inputs and outputs

Clip Trabeculae: *Remove trabecular struts and soft tissue remnants from section borders* (Manual)	
Input	Original, unmodified brightfield cross‐section
Output	Brightfield cross‐section with peripheral tissue separated from cortex
Wand ROI Selection: *Clear regions external to the cortex* (Manual)	
Input	Brightfield cross‐section exported by *Clip Trabeculae*
Output	(1) Brightfield cross‐section cleared (black) outside the cortex (2) Zip file of border region‐of‐interest (ROI) overlays for Total, Endosteal, and Cortical Areas
ROI Touchup: *Optionally nudge and smooth border ROIs* (Manual)	
Input	(1) Original, unmodified brightfield cross‐section (2) Border ROI set exported by Wand ROI Selection
Output	(1) Brightfield cross‐section cleared (black) outside the cortex according to modified border ROIs (2) Modified and/or smoothed Border ROI set
Image Pre‐Processing: *Contrast enhancement, noise reduction, and color channel splitting* (Automatic)	
Input	Brightfield cross‐section(s) with externally cleared borders, as single image or folder of images
Output	For each loaded image (in separate named folders for batch processing): (1) TIFF image of preprocessed brightfield cross‐section, including combined (RGB) and/or individual Red, Blue, Green grayscale color channels as selected by user (2) Text file log of user‐selected preprocessing operations (3) Border ROI set detected by cleared section borders
Pore Extractor: *Identification of pore spaces* (Semi‐automatic)	
Input	Brightfield cross‐section with externally cleared borders, optionally preprocessed
Output	(1) Pore ROI set (2) Text file log of user‐selected thresholds for pore binarization (3) Text file log of user‐selected morphological modification and size/circularity filtering settings (4) Border ROI set detected by cleared section borders
Pore Modifier: *Inspection and correction of pore spaces* (Manual)	
Input	(1) Brightfield cross‐section with externally cleared borders, optionally preprocessed (2) Pore ROI set exported by *Pore Extractor* or a previous *Pore Modifier* session
Output	User‐modified pore ROI set(s), each appended with a three‐digit ID number
Pore Analyzer: *Morphometric pore analysis with regional and pore type subdivisions* (Automatic)	
Input	(1) Brightfield cross‐section with externally cleared borders (2) Finalized pore ROI set exported by *Pore Modifier*
Output: Summary Statistics	(1) Spreadsheet (.csv) of cross‐sectional geometry measurements (2) Spreadsheet (.csv) of total section pore morphometry, subdivided by pore type (3) Spreadsheet (.csv) of regional pore morphometry, subdivided by pore type
Output: Cross‐Sectional Geometry	(1) Border ROI set detected by cleared section borders (2) TIFF images of Total Area (Tt.Ar), Endosteal Area (Es.Ar), and Cortical Area (Ct. Ar)
Output: Regions	(1) TIFF image of Cortical Area with overlay line(s) for regional subdivisions (2) ROI files for each regional subdivision (3) Spreadsheet (.csv) of regional gray means for each pore ROI
Output: Pore Types	(1) TIFF images of binarized total, cortical, and trabecularized pore spaces (2) Pore ROI sets for total, cortical, and trabecularized pore types, colorized by regional subdivision (3) Spreadsheet (.csv) of individual pores listing regional assignment, pore type, and morphometry

### 2.3 Periosteal and endosteal clearing

**FIGURE 2:**
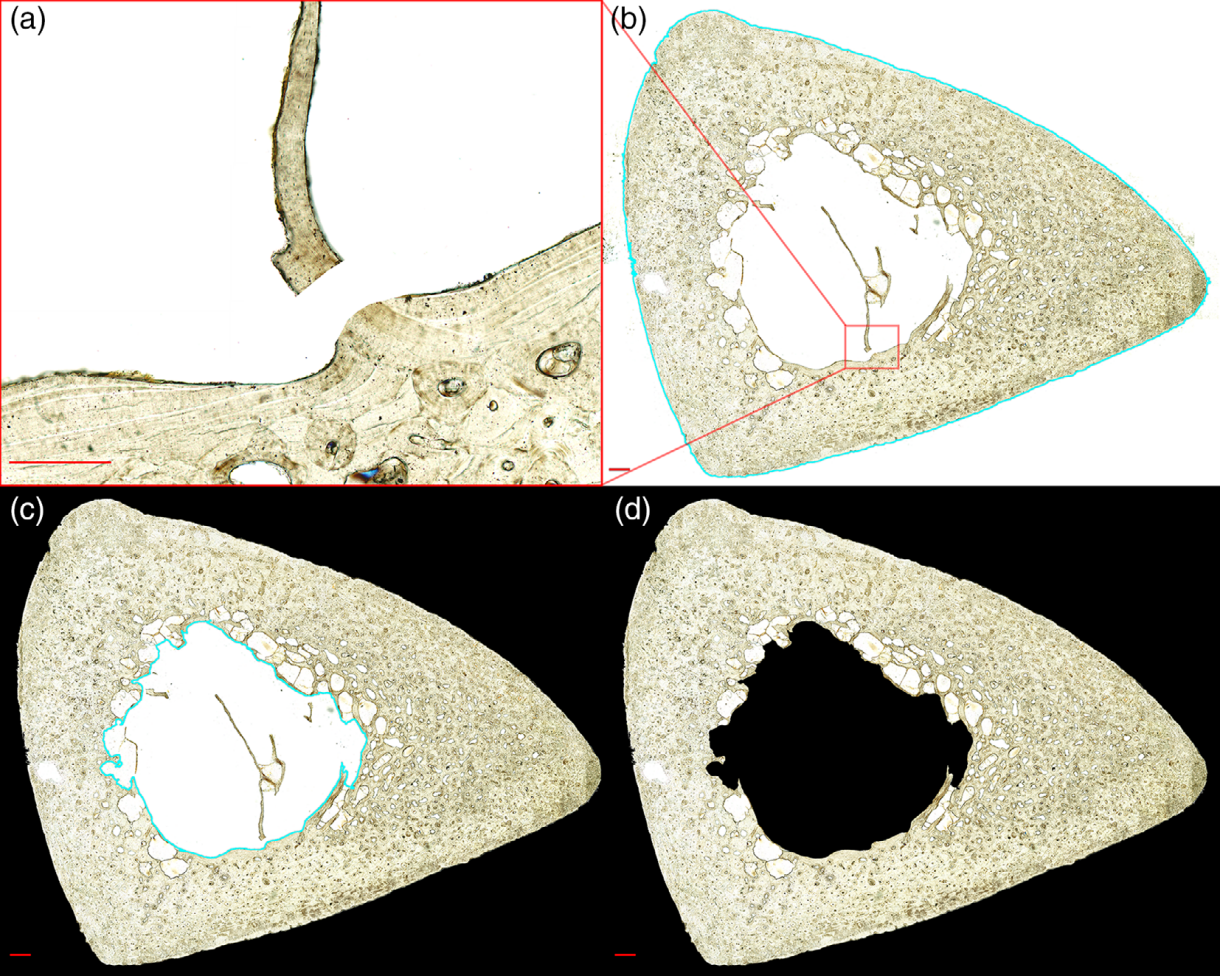
Periosteal and endosteal clearing tools are demonstrated on a human tibia. The *Clip Trabeculae* tool allows users to separate trabecular struts and tissue remnants from the cortex using the white paintbrush tool (a). The *Wand ROI Selection* tool selects the off‐white space outside the periosteum (b) or contained within the endosteum (c) by tracing connected pixels within a user‐selected pixel brightness range (b = 193, c = 23) of an initially clicked pixel. The final cross‐section is cleared (black) external to the bone cortex (d). Scale bar is 0.5 mm (a) and 1 mm (b–d)

Prior to pore extraction, spaces outside the section borders must be cleared of non‐cortical tissue, including large soft tissue remnants and trabecular bone struts. Pore Extractor 2D provides tools to expedite section border clearing (Figure 2).

#### 2.3.1 Clip Trabeculae Tool

This macro provides keyboard shortcuts for the ImageJ *Brush* tool, allowing the user to modify brush size, seal cracks through the cortex (black brush) and separate trabecular struts and soft tissue remnants from the cortex (white brush) (Figure 2a).

#### 2.3.2 Wand ROI Selection Tool

This macro provides keyboard shortcuts to automatically clear spaces external to the periosteum and endosteum. The user selects the *Wand* tool and clicks a pixel immediately outside the bone section. All connected pixels with the same brightness value are selected. The user then increases the tolerance of the *Wand* tool to expand the brightness range for connected pixel selection. This technique can often select most or all of the empty space bordering the bone section (Figure 2b,c). The selected space is then “cleared,” meaning that it is converted to the background value of absolute black (Figure 2d). Borders can be cleaned of remaining debris, such as mounting medium bubbles or adhered soft tissue, with the ImageJ *Freehand Selection* tool or the ImageJ *Brush* tool. Total area, endosteal area, and cortical area borders are automatically calculated from the modified image and are exported as region‐of‐interest (ROI) overlays.

#### 2.3.3 ROI Touchup Tool

This optional macro allows users to further modify the total area and endosteal area borders exported by *Wand ROI Selection Tool*. The users loads the original, unmodified brightfield image, followed by the *Borders_RoiSet.zip* file exported by *Wand ROI Selection Tool*. A section border can be nudged inward or outward with the *Selection Brush* tool. A section border can also be converted to nodes (adjustable vertex points) that are separated by a user‐selected pixel distance. This sub‐tool is a modification of the ImageJ Nodes macro developed by Dominguez and Agnew (2019). Node conversion smooths pixelized chatter along the section border. When the user exits the macro, a new “cleared” image (Figure 2d) is exported according to the revised section borders.

### 2.4 Image Pre‐processing tool

**FIGURE 3:**
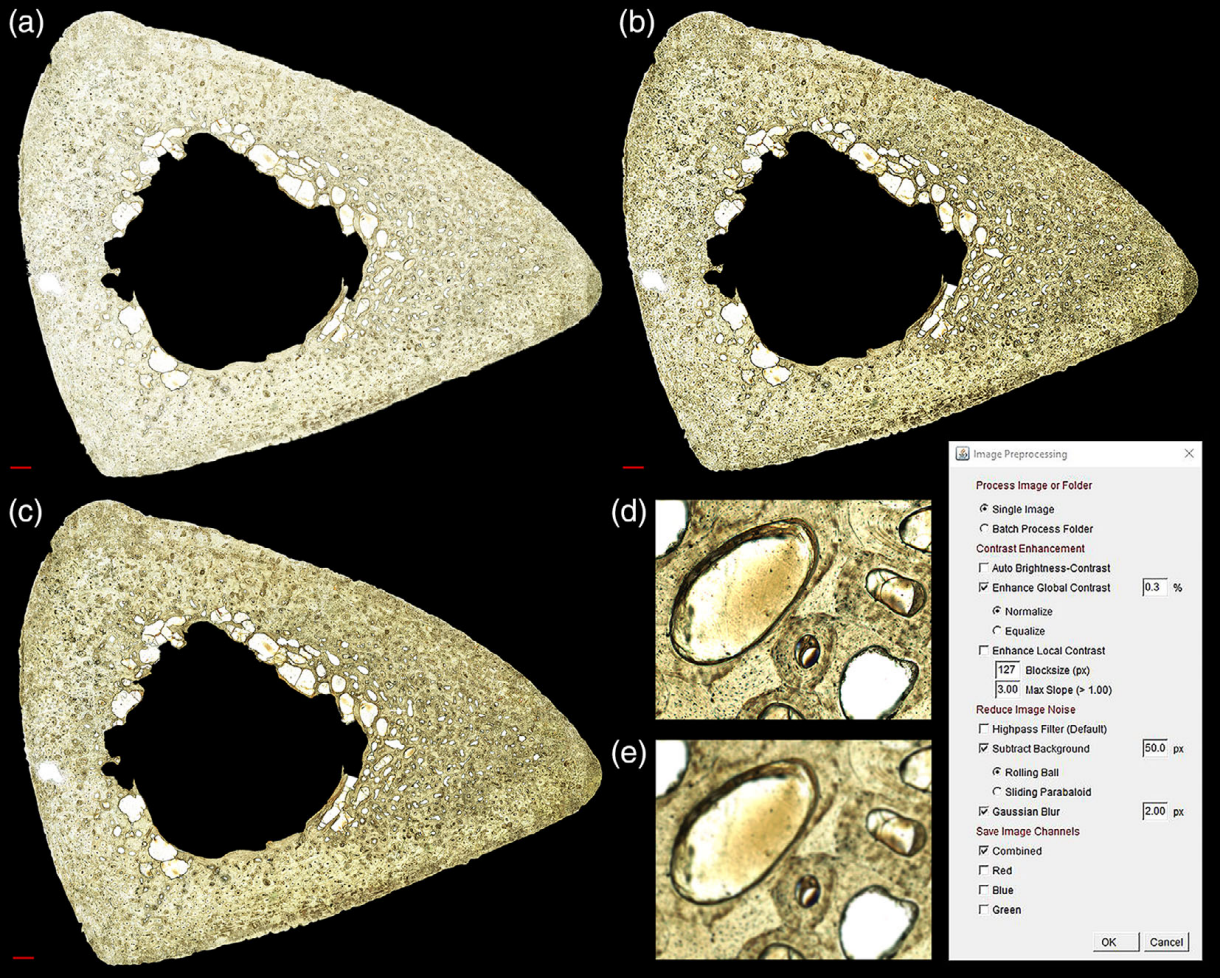
A sample image pre‐processing sequence from the *Image Pre‐Processing* tool is demonstrated on a human tibia. The original image (a) is contrast enhanced with histogram equalization (b) followed by background subtraction to minimize uneven illumination (c). Smoothing the image reduces noise for improved pore extraction, as demonstrated before (d) and after (e) a Gaussian blur. Scale bar is 1 mm (a–c) and 100 μm (d and e)

The *Image Pre‐Processing* tool (Figure 3) is an optional step for image contrast enhancement, noise removal, and stain reduction. These pre‐processing options are available as default ImageJ plugins, but must be accessed individually from separate menus, and may require a lengthy processing time for each command. This tool allows users to program a sequence of pre‐processing commands to run automatically on one image or on a folder of multiple images. Following selection of these commands, users can re‐order the workflow, although the default order is recommended. Processing speed is expedited by a batch mode that does not display the images. Recommended starter settings are provided in the supplemental material.

Image contrast can be enhanced in dim images by auto‐optimizing brightness‐contrast, which limits the minimum and maximum pixel brightness values. Contrast between dark and light structures can be enhanced globally for all pixels through histogram normalization (stretching) or equalization, or locally for each pixel based on a user‐selected blocksize (pixel neighborhood).

Image noise can be reduced by applying a highpass filter (ImageJ *FFT Bandpass Filter*), which subtracts a Gaussian blurred version of the image to correct uneven illumination. Another option for uneven illumination correction is background subtraction, which calculates and subtracts a background from each pixel based on a large radius ball or paraboloid. Image noise can also be smoothed with a Gaussian blur filter.

Pre‐processed images can be saved as a combined RGB (color) image channel, and as grayscale images representing red, blue, or green image channels. Histological staining of bone tissue may be reduced by selecting an individual grayscale color channel to load in *Pore Extractor* and *Pore Modifier*.

### 2.5 Pore Extractor tool

**FIGURE 4:**
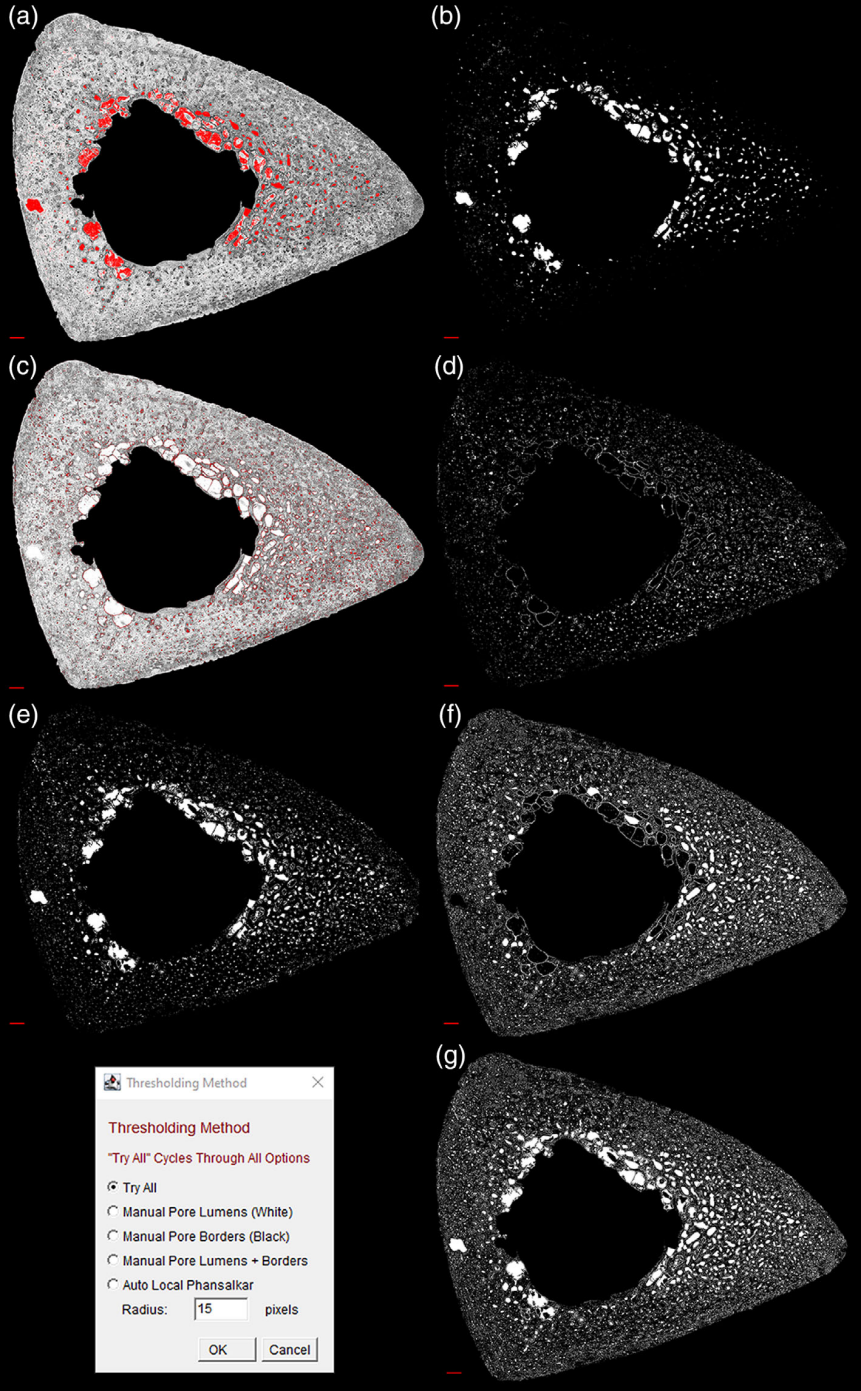
A sample pore thresholding sequence from the *Pore Extractor* tool is demonstrated on a human tibia. Manual options prompt the user to visualize (red overlay) a global threshold for converting pore lumens (a, b) and pore borders (c, d) to absolute white. Additional thresholding options include a combined image of manual pore lumens and borders (e), a completely automated local threshold with a Phansalkar algorithm (f), or all thresholding options combined (g). Scale bar is 1 mm

**FIGURE 5:**
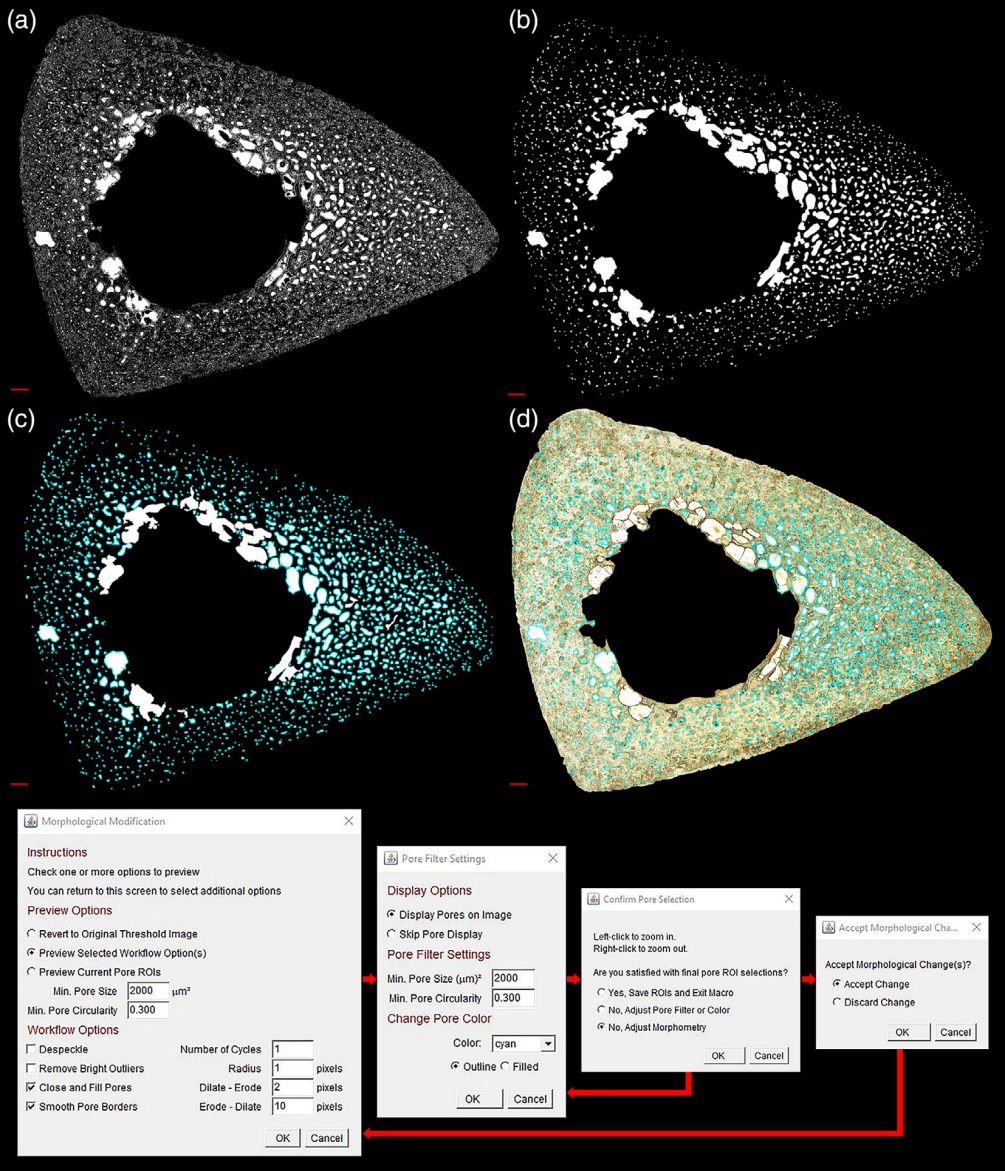
A sample pore morphological modification sequence from the *Pore Extractor* tool is demonstrated on a human tibia. The binarized image of pore spaces (a; from Figure 4g) undergoes two closing and filling cycles and 10 smoothing cycles (b). Spaces are filtered by user‐selected size (shown here as >2000 μm^2^) and circularity (shown here as >0.300) to reduce noise selection. Pore selections (cyan) are simultaneously superimposed on the morphometrically modified image of binarized pore spaces (c) and the cleared brightfield image (d). Pore selection color or fill can be changed for easier visualization. Users can repeatedly adjust morphological closing and pore size/shape filtering in a modification loop. Scale bar is 1 mm

The *Pore Extractor* tool (Figures 4 and 5) semi‐automatically isolates initial pore spaces, with user‐guided thresholding and morphological border closing. Recommended starter settings are provided in the supplemental material.

#### 2.5.1 Thresholding

Users can load any cleared brightfield image, optionally pre‐preprocessed. This image is converted to 8‐bit grayscale, and initial pore spaces are binarized with the user's choice of thresholding mechanisms (Figure 4). Manual threshold options prompt the user to visualize and adjust a global threshold (0–255) for pore structures, superimposed on the image as a red overlay. *Manual Pore Lumens (White)* converts near‐white pixels above a user‐set threshold, representing pore lumens, to absolute white (255). *Manual Pore Borders (Black)* converts dark pixels below a separate user‐set threshold, representing pore borders, to absolute white. *Manual Pore Lumens + Borders* combines the pore lumens and borders in a single image. *Auto Local Phansalkar* uses a Phansalkar algorithm (Phansalkar et al., 2011) for low‐contrast images to individually binarize each pixel based on the pixel brightness in its local neighborhood (default = 15 pixels). Following each thresholding operation, binarized pore spaces are closed through single‐pixel dilation and erosion, and holes within pores are filled. *Try All* cycles through each of these thresholding options, generates an image combining all thresholding options, and prompts the user to select the best result.

#### 2.5.2 Morphological modification

Binarized pore spaces can be de‐noised, closed, smoothed, and filtered by minimum size and/or circularity to improve pore selection (Figure 5). Dilations and erosions for pore closing and smoothing are performed with *EDM Binary Operations* from the BioVoxxel toolbox, which prevents deformation artifacts under large numbers of cycles (Brocher, 2015). Any spaces that pass user‐set minimum size and circularity filters are superimposed on both the brightfield image and the morphologically modified binarized pore image. This subtool is a repeating loop, where users can change the modification sequence and visualize the resulting pore selections.

### 2.6 Pore Modifier tool

**FIGURE 6:**
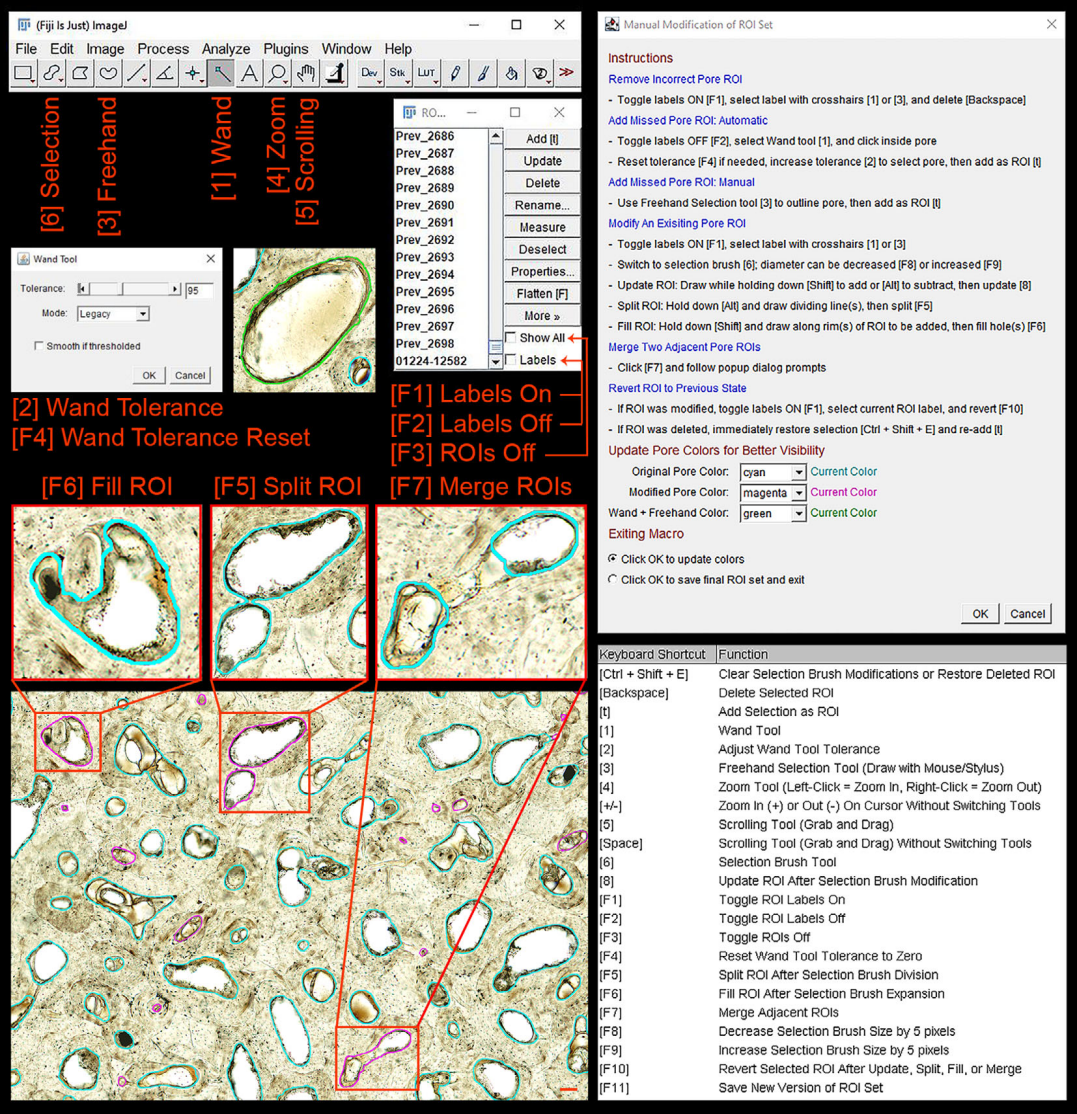
The *Pore Modifier* tool provides keyboard shortcuts for pore region‐of‐interest (ROI) inspection and correction. Users can set color codes for original (default = cyan), modified (default = magenta), and new (default = green) pore ROIs. Pore Modifier provides keyboard shortcuts for toggling ROI and ROI label visibility [F1, F2, F3], and quickly selecting ImageJ toolbar buttons, including the wand tool [1], wand tool tolerance [2], freehand selection [3], zoom [4], scroll while zoomed [5], and selection brush [6]. Many pores can be automatically selected by clicking the pore border or lumen and increasing wand tool tolerance [2]. Custom macros are available to update borders [8], split [F5], fill [F6], or merge [F7] existing pore ROIs using the selection brush tool [6], and to revert any modified ROI [F10]. Users can auto‐save a copy of the current ROI set at any time [F11]. Scale bar is 100 μm

Due to section debris, faint pore borders, and subsurface transverse connections, not all pores will be accurately selected on the histological image. The *Pore Modifier* tool (Figure 6) provides keyboard shortcuts for quick inspection and correction of the *Pore Extractor* pore ROI set. Pore ROIs can be overlaid on any cleared brightfield image loaded into *Pore Modifier*. There is also a load setting to superimpose pore ROIs on each color channel (RGB, Red, Green, Blue), so that users can select the best option. The user can also choose to skip *Pore Extractor* and select pores completely manually using *Pore Modifier* utilities.

Keyboard shortcuts allow users to easily add, delete, and modify (update/fill/split/merge/revert) pore ROIs (Table S1). A digital tablet or a touchscreen computer monitor with a stylus is useful for this modification. Many pores can be automatically selected by clicking the pore border or lumen with the *Wand* tool and modulating *Wand Tool Tolerance*. Color codes allow users to track original, modified, and newly added pore spaces. Colors can be modified within the macro dialog or under *Set Global Preferences*. An auto‐save function allows users to save a copy of the modified ROI set at any time. Previously modified ROI sets can be loaded, and new ROI sets will continue to append numerically in the output folder. This allows users to modify the same ROI set over multiple discrete sessions of *Pore Modifier*, which is useful for inspecting large cross‐sections.

### 2.7 Pore analyzer tool

**FIGURE 7:**
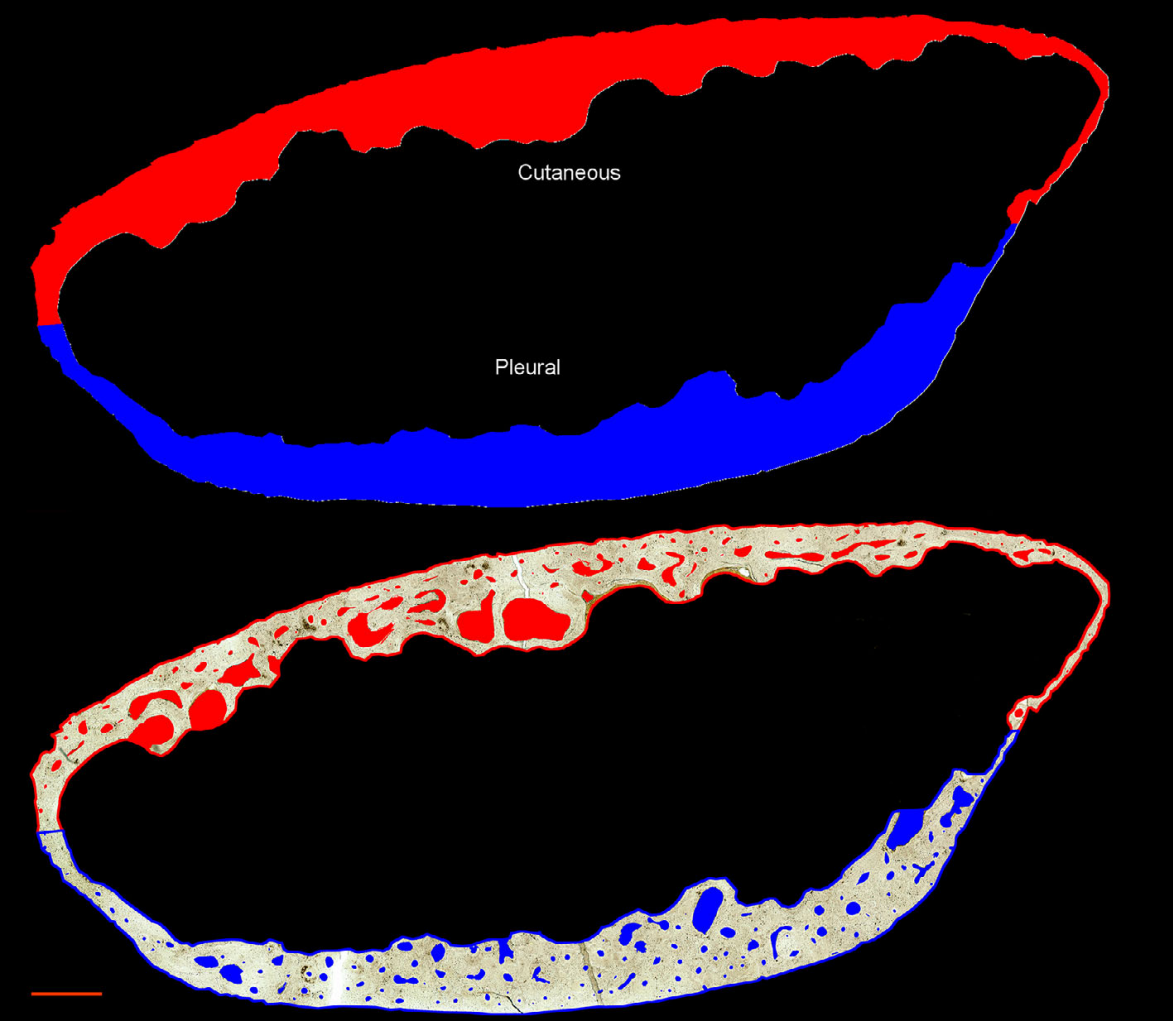
Regional subdivision from the *Pore Analyzer* tool is demonstrated on a human sixth rib. The major axis subdivides the cutaneous (red) and pleural (blue) cortices. The toolkit guesses the more circular region as the pleural cortex, which users can confirm or switch. Each pore ROI is sorted into the region that contains the majority of its individual pore area. Scale bar is 1 mm

**FIGURE 8:**
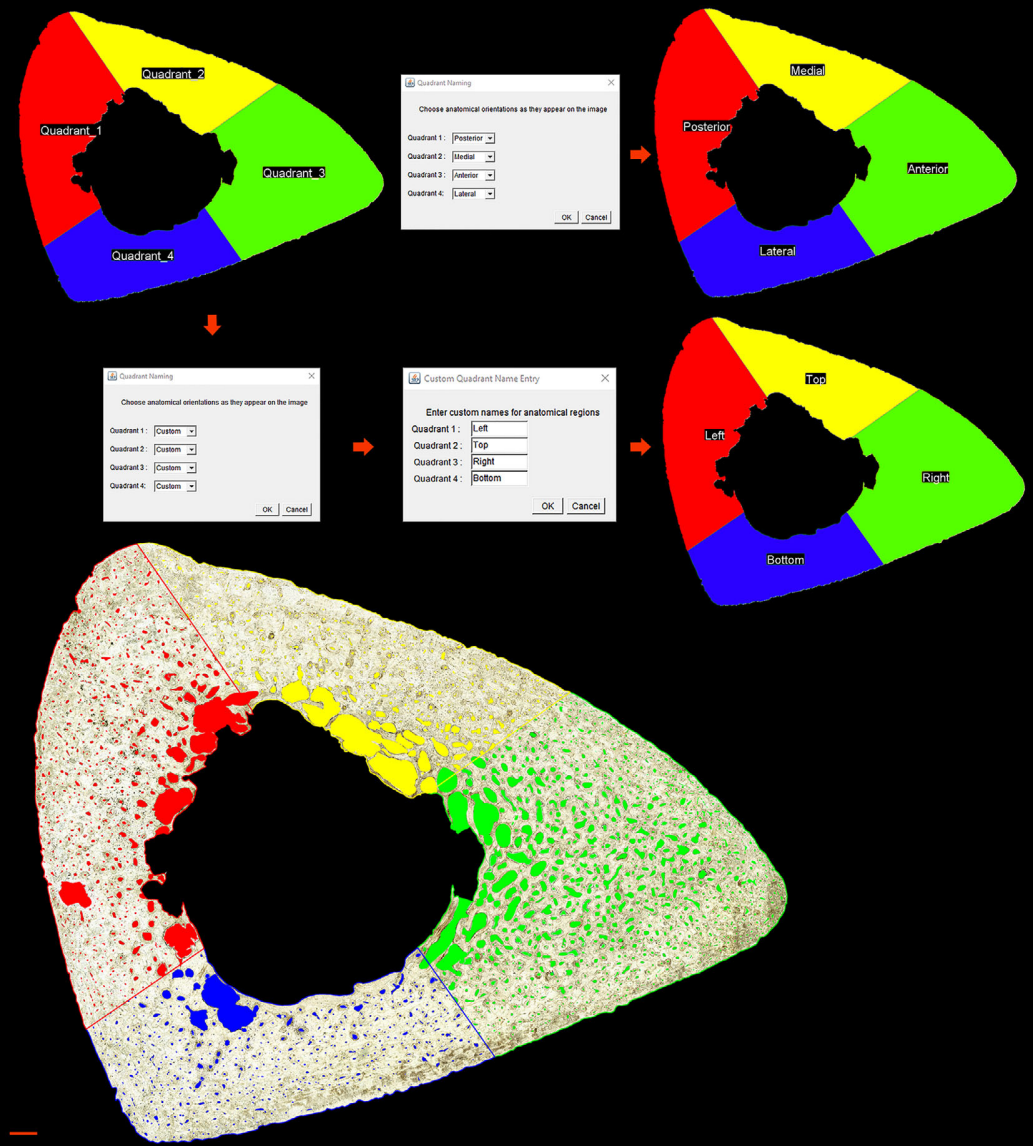
Regional subdivision from the *Pore Analyzer* tool is demonstrated on a human tibia. The starting axis is drawn using the major axis, and rotated 45° and then 90° to draw anatomical quadrants. The user is prompted to select common anatomical names or enter custom names. Each pore ROI is sorted into the region that contains the majority of its individual pore area. Scale bar is 1 mm

**FIGURE 9:**
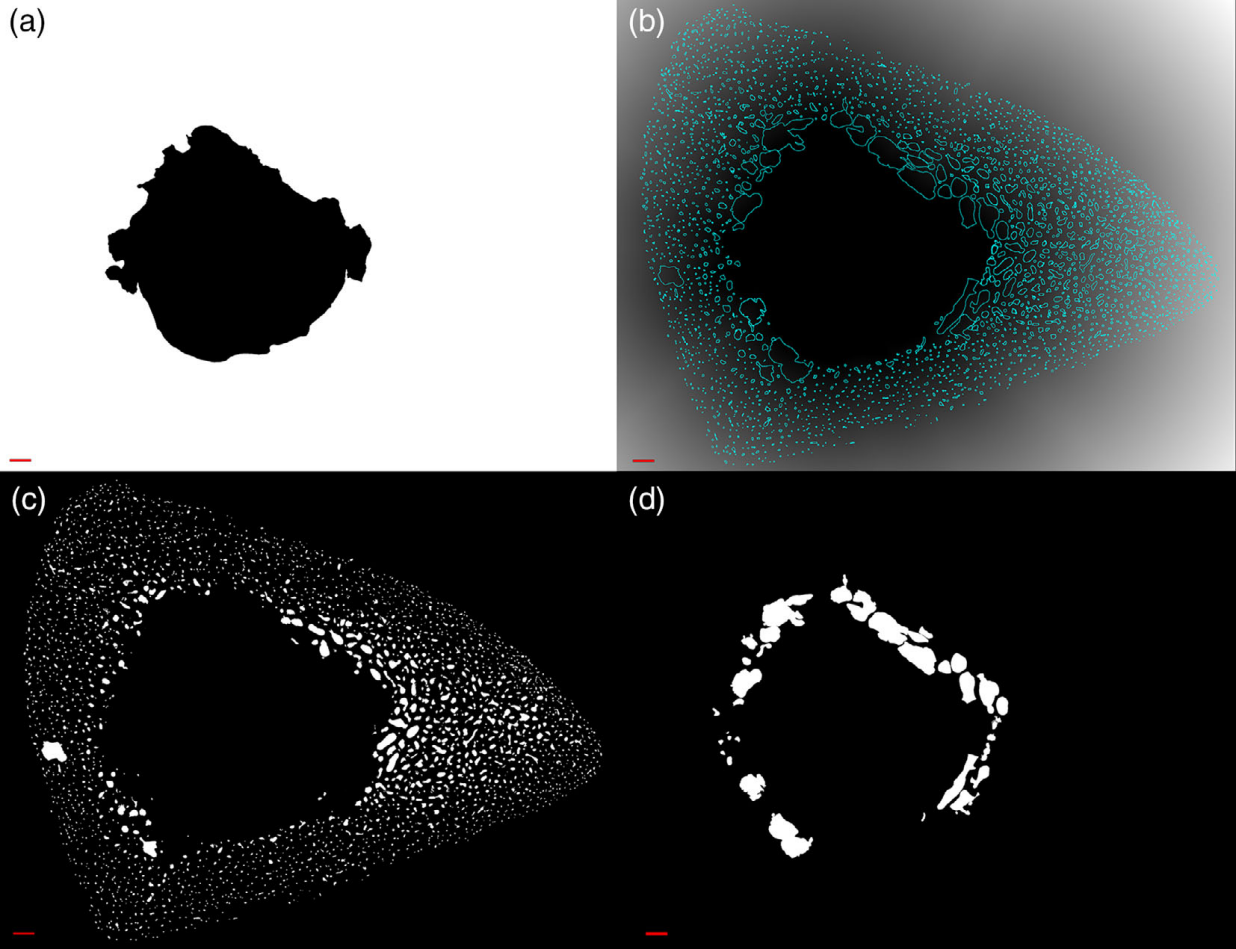
Pore type subdivision from the *Pore Analyzer* tool is demonstrated on a human tibia. The marrow cavity is converted to background (a) and then to a 32‐bit Euclidean Distance Map with pore ROIs (cyan) superimposed (b). The minimum gray value measured inside each pore ROI corresponds to its minimum distance, in pixels, from the endosteum. Pore are classified as “cortical” (minimum pore diameter < minimum endosteal distance) (c) or “trabecularized” (minimum pore diameter ≥ minimum endosteal distance) (d). Scale bar is 1 mm

The *Pore Analyzer* tool (Figures 7, 8, 9) automatically subdivides finalized pore ROIs by anatomical region and pore type (cortical or trabecularized). Summary statistics for pore morphometry (Table 2) are exported for total pores, cortical pores, and trabecularized pores across the whole cross‐section and in each anatomical region.

**TABLE 2:** Pore analyzer summary statistics

	Morphometric variable name	Calculation
Cross‐sectional geometry	Total Area (mm^2^)	Measured from cleared section borders
	Endosteal Area (mm^2^)	Measured from cleared section borders
	Cortical Area (mm^2^)	Total Area − Endosteal Area
	% Cortical Area	(Cortical Area/Total Area) * 100
	% Bone Area Total Porosity	[(Cortical Area − Total Pore Area)/Total Area] * 100
	% Bone Area Cortical Porosity	[(Cortical Area − Cortical Pore Area)/Total Area] * 100
	% Bone Area Trabecularized Porosity	[(Cortical Area − Trabecularized Pore Area)/Total Area] * 100
	% Endosteal Area	(Endosteal Area/Total Area) * 100
	Parabolic Index (*Y*)	(Cortical Area * Endosteal Area)/(Total Area^2^)
	Imin (mm^4^)	Calculated by BoneJ
	Imax (mm^4^)	Calculated by BoneJ
	Zpol (mm^4^)	Calculated by BoneJ
Summary measurements (region and type)	Cortical Area (μm^2^ and mm^2^)	Measured within region ROI
	% Cortical Area	(Region Cortical Area/Total Pore Area) * 100
	Bone Area (μm^2^ and mm^2^)	Region Cortical Area − Total Pore Area
	% Bone Area	[(Region Cortical Area − Total Pore Area)/Total Area] * 100
	Percent Porosity (%)	(Total Pore Area/Region Cortical Area) * 100
	Pore Density (1/μm^2^ and 1/mm^2^)	Total Pore Number/Region Cortical Area
	Total Pore Number	Total number of pores of that type within that region
	Total Pore Area (μm^2^)	Total area of pores of that type within that region
	Mean Pore Area (μm^2^)	Calculated by ROI Manager
	Mean Pore Perimeter (μm)	Calculated by ROI Manager
	Mean Pore Circularity (0–1)	Calculated by ROI Manager as 4π * Area/Perimeter^2^
	Mean Pore Max Feret Diameter (μm)	Calculated by ROI Manager (Maximum Caliper Diameter)
	Mean Pore Min Feret Diameter (μm)	Calculated by ROI Manager (Minimum Caliper Diameter)
	Mean Pore Aspect Ratio	Calculated by ROI Manager as Major Axis/Minor Axis
	Mean Pore Roundness	Calculated by ROI Manager as 4 * Area/(π * Major Axis^2^)
	Mean Pore Solidity (0–1)	Calculated by ROI Manager as Area/Convex Area

#### 2.7.1 Regional subdivision

Total area, endosteal area, and cortical area are detected by cleared section borders. The cortical area image is used to calculate the section centroid, major axis, and additional cross‐sectional geometry values (Imin, Imax, and Zpol) using the BoneJ plugin Slice Geometry (Doube et al., 2010). Users are prompted to choose the section type as a rib or long bone.

Ribs are divided into cutaneous and pleural halves along the major axis, consistent with previous work (Agnew & Stout, 2012; Dominguez & Agnew, 2016) (Figure 7). The user can also switch to the minor axis, which may be appropriate for ribs that are elongated along the cutaneous‐pleural axis, where use of the major axis can result in a superior–inferior subdivision. The toolkit will guess that the more circular ROI is the pleural cortex, as it contains the costal groove, but these designations can be switched by the user.

Long bones are divided into quadrants using the longest anatomical axis of the cross‐section (Figure 8), such as the anterior–posterior axis in the midshaft femur and tibia. If the user selects *Section Alignment with Image Borders*, it is assumed that the cross‐section is already rotated such that the longest anatomical axis aligns with the horizontal or vertical image border. In this case, a horizontal line is drawn through the section centroid as the starting axis. If the user indicates *Section Major Axis*, the section can have any orientation, and the major axis detected by BoneJ is used as the starting axis. The user can also switch to the minor axis as the starting axis, which may be required for unusually wide cross‐sections. The starting axis is rotated 45° and then 90° to obtain coordinates for the four quadrants. The user is asked to assign names to each displayed quadrant, choosing from preset names (Anterior, Posterior, Medial, Lateral, Superior, or Inferior) or “Custom,” which allows the user to enter any text for a region name.

Each pore ROI is automatically assigned to the region that contains the majority of its pore area. Each region, in turn, is binarized to 8‐bit absolute white (255), and the mean gray value for all pore ROIs is recorded. Pores that lie fully within a given region will score 255 for that region and 0 for any other region. Pores that lie on a regional boundary will score between 0 and 255 in every region they partially occupy. A boundary pore is sorted into the region that contains the higher mean gray value, which corresponds to the larger portion of its pore area.

#### 2.7.2 Pore type subdivision

A pore is considered “trabecularized” if its minimum diameter exceeds its minimum distance from the endosteum (Keshawarz & Recker, 1984). Minimum diameter is measured as the minimum feret (caliper) diameter for each pore. To calculate minimum distance from the endosteum, the marrow cavity is converted into a 32‐bit Euclidean Distance Map (Figure 9). The minimum gray value measured inside each pore ROI corresponds to its minimum distance, in pixels, from the endosteum. Each pore ROI is classified as “cortical” (diameter < distance) or “trabecularized” (diameter ≥ distance).

### 2.8 Validation sample and statistical analysis

Pore Extractor 2D was validated against traditional manual annotation on a histological sample (*n* = 30) of mid‐shaft, mid‐thoracic human ribs (Table 3). These ribs originated from individuals donated to The Ohio State University's Whole Body Donation Program or Lifeline of Ohio, histologically prepared as previously described (Dominguez & Agnew, 2016). In brief, rib cross‐sections were photographed under brightfield light at 40× or 100× magnification with an Olympus VS120 slide scanner. A qualified observer (VMD) used a digitizing pen and tablet to manually identify and outline pore spaces on each cross‐section. Each hand‐drawn pore outline was saved to a pore ROI set in ImageJ (Dominguez & Agnew, 2016, 2019). Another qualified observer (MEC) assessed these same digital images with Pore Extractor 2D. Both the manual and toolkit‐derived ROI sets were morphometrically characterized (Table 3) using the *Pore Analyzer* macro tool.

**TABLE 3:** Sample demographics

Statistic		Validation set	Intraskeletal set
# Individuals		30	9
# Elements		30	27
Age (years)	Range	17–32	39–82
	Mean	45.9	61.3
	Std.Dv.	22.1	12.4
# Males		19	6
# Females		12	3
Midshaft elements		Ribs 4, 5, 6, 7	Femur, Tibia, Rib 6

All statistical analyses were performed in R (The R Foundation, v. 4.1.1). An alpha of *α* = 0.05 was used for all significance testing. Paired *t*‐tests from package *rstatix* (Kassambara, 2021) compared morphometric variables derived from manual and Pore Extractor 2D annotation (Table 4). Effect size was assessed with Cohen's *d* (Cohen, 1969) from package *lsr* (Navarro, 2019). Morphometric variables that were not normally distributed (Shapiro–Wilk) were assessed with a Wilcoxon Signed Rank test from core R package *stats* and a Wilcoxon effect size from package *rstatix*.

**TABLE 4:** Validation of Pore Extractor 2D versus manual annotation

	Variable	*p*‐value	Effect size	Effect magnitude	Directionality
Aggregate	Bone Area^a^	0.589	0.100	‐	PE2 > Manual
	% Bone Area^a^	0.609	0.094	‐	PE2 > Manual
	Percent Porosity^a^	0.859	0.033	‐	PE2 < Manual
	Pore Density^b^	0.226	0.256	Small	PE2 < Manual
	Total Pore Number^a^	0.177	0.253	Small	PE2 < Manual
	Total Pore Area^a^	0.588	0.100	‐	PE2 < Manual
Pore means	Area^b^	0.697	0.071	Small	PE2 > Manual
	Perimeter^a^	***<0.001***	0.992	Large	PE2 > Manual
	Circularity^a^	***<0.001***	2.027	Large	PE2 < Manual
	Max Feret Diameter^a^	***0.0189***	0.454	Small	PE2 > Manual
	Min Feret Diameter^a^	0.469	0.134	‐	PE2 < Manual
	Aspect Ratio^b^	***<0.001***	0.699	Large	PE2 > Manual
	Roundness^a^	***<0.001***	1.068	Large	PE2 < Manual
	Solidity^b^	***<0.001***	0.871	Large	PE2 < Manual

^a^ Normal; Paired *t*‐test and Cohen's *d* effect size (Small = 0.2–<0.5, Medium = 0.5–<0.8, Large ≥0.8).

^b^ Not normal; Wilcoxon Signed‐Rank test and effect size (Small = 0.1–<0.3, Medium = 0.3–<0.5, Large ≥0.5).

*Note*: Significant (*p* < 0.05) *p*‐values are bolded and italicized.

### 2.9 Intraskeletal sample and statistical analysis

Intraskeletal and regional analyses were demonstrated on a histological sample of matched femora, tibiae, and sixth ribs from the same individuals (*n* = 9) (Table 3). These samples originated from individuals donated to Washington University in St. Louis and the University of Missouri, Columbia, histologically prepared as previously described (Stewart et al., 2015). Cross‐sections were photographed under brightfield light at 100× magnification with an Olympus BX51 microscope. Additional white‐balancing in Adobe Photoshop compensated for epoxy resin yellowing (Marty, 2007). Pore spaces were extracted, modified, and analyzed on these digital images by a qualified observer (MEC) using Pore Extractor 2D.

Linear Mixed Models (LMM) from package *lme4* (Bates et al., 2015) assessed intraskeletal and regional variation in morphometry. LMM were chosen because they can correct for random effects, which here included chronological age, sex, and the repeated measurements (skeletal element or region) from within the same individual. Each morphometric variable was centered and scaled. If the residuals of a given LMM were not normally distributed (Shapiro–Wilk) or were heteroskedastic (Levene's Test), that morphometric variable was also transformed with Tukey's Ladder of Powers. This function from package *rcompanion* finds the lambda that maximizes the *W* statistic of the Shapiro–Wilk test (Mangiafico, 2018).

Percent cortical area was compared to percent bone area, as variably calculated through the removal of total, cortical, or trabecularized pores, using model: % Area ~ Calculation Method * Bone + (1|Individual) + (1|Age) + (1|Sex).

Cross‐sectional geometry and pore morphometric variables were compared between femora, tibiae, and ribs using model: Morphometric Variable ~ Bone * Pore Type + (1|Individual) + (1|Age) + (1|Sex).

Pore morphometry was compared between each region of a skeletal element using model: Morphometric Variable ~ Region * Pore Type + (1|Individual) + (1|Age) + (1|Sex).

Following LMM modeling, patterns of significance were confirmed by fitting a generalized LMM with a Markov Chain Monte Carlo approach, to draw on the high accuracy of Bayesian modeling. This secondary analysis used package *MCMCglmm* (Hadfield, 2010) with a parameter‐expanded prior and 60,000 iterations.

The variation that each model explained in each morphometric variable was quantified using a pseudo *R* ^2^ from package MuMIn (Barton, 2019). Marginal *R* ^2^ is the variance explained by fixed factors alone (calculation method, bone, region, and/or pore type), while Conditional *R* ^2^ is the variance explained by both fixed factors and random effects (age, sex, and individual).

For models that detected significant differences (*p* < .05) within fixed factors, *post‐hoc* analyses were carried out with package *emmeans* (Lenth, 2020). Effect size was quantified using Cohen's *d* from package *EMAtools* (Kleiman, 2017) with thresholds of small (*d* = 0.2), medium (*d* = 0.5), or large (*d* = 0.8) effect size (Cohen, 1969).

In the statistical analysis, age and sex never reached significance as random effects, potentially due to the bias towards males and older individuals in the small pilot sample. The only significance detected for the repeated measures of individuals was for femoral regional percent porosity, which was particularly low in the lateral quadrant of an 82‐year‐old male.

## 3 RESULTS

### 3.1 Validation against manual annotation

Pore Extractor 2D replicated manual annotation of percent porosity, pore density, and mean pore size (Table 4) in the validation sample of midshaft, midthoracic human ribs (*n* = 30). No statistically significant differences were identified in bone area, percent bone area, percent porosity, pore density, total pore number, total pore mean pore area, or mean pore minimum feret diameter. Pore Extractor 2D did significantly increase mean pore perimeter, reflecting a closer fit to the pixelated color boundary between pore and tissue. Pores were also significantly less uniformly circular (circularity, roundness, and solidity) and significantly more elongated (maximum feret diameter, and aspect ratio), compared to manual annotation.

### 3.2 Intraskeletal variability in section areas

Analysis of the intraskeletal sample (*n* = 9) found that correction for porosity (“percent bone area”) always significantly reduced percent cortical area, regardless of the skeletal element or type of porosity (Marg. *R* ^2^ = 66.8%) (Tables S2 and S3).

Cross‐sectional analyses (Table 5, Tables S4 and S5) found that the femur significantly exceeded both the tibia and the rib in absolute values of total area (Marg. *R* ^2^ = 90.4%), endosteal area (Marg. *R* ^2^ = 67.0%), and cortical area (Marg. *R* ^2^ = 90.3%). However, the femur and tibia did not significantly differ in any percent area metrics, which were corrected for total area. Both the femur and tibia significantly exceeded the rib in percent cortical area (Marg. *R* ^2^ = 65.2%) and percent bone area corrected for total porosity (Marg. *R* ^2^ = 69.3%), cortical porosity (Marg. *R* ^2^ = 64.7%), and trabecularized porosity (Marg. *R* ^2^ = 72.3%). The rib significantly exceeded both the femur and tibia in percent endosteal area (Marg. *R* ^2^ = 65.2%).

**TABLE 5:** Significant post‐hocs for cross‐sectional areas

Variable	Marg. *R* ^2^ (%)	Femur ‐ Tibia	Femur ‐ Rib	Tibia ‐ Rib
Total Area	90.4	>	>	>
Endosteal Area	67.0	>	>	>
Cortical Area	90.3	>	>	>
% Endosteal Area	65.2	‐	<	<
% Cortical Area	65.2	‐	>	>
% Bone Area Total Porosity	69.3	‐	>	>
% Bone Area Ct. Porosity	64.7	‐	>	>
% Bone Area Tb. Porosity	72.3	‐	>	>

### 3.3 Intraskeletal variability in pore morphometry

Analyses of pore type over whole cross‐sections (Table 6, Tables S6 and S7) found no intraskeletal differences in total percent porosity (Marg. *R* ^2^ = 32.0%). The interaction contrast did reveal intraskeletal variation when pore types were considered separately. Cortical percent porosity was significantly elevated in the femur and tibia over the rib. Trabecularized percent porosity was significantly elevated in the rib over the femur and tibia. The rib significantly exceeded both the femur and tibia in total pore density (Marg. *R* ^2^ = 95.2%), which the interaction contrast indicated was due to trabecularized pores alone.

**TABLE 6:** Significant post‐hocs for intraskeletal comparisons

Variable		Marg. *R* ^2^ (%)	Bone			Type	Cortical pores			Trabecularized pores			Femur	Tibia	Rib
			Femur ‐ Tibia	Femur ‐ Rib	Tibia ‐ Rib	Ct. ‐ Tb.	Femur ‐ Tibia	Femur ‐ Rib	Tibia ‐ Rib	Femur ‐ Tibia	Femur ‐ Rib	Tibia ‐ Rib	Ct. ‐ Tb.		
Agg.	Percent Porosity	32.0						>	>		<	<			<
	Pore Density	95.2		<	<	>					<	<	>	>	>
Pore Means	Area	88.6		>	>	<									
	Perimeter	88.7		>	>	<									
	Circularity	54.0		<	<	>									
	Max Feret Diameter	87.9		>	>	<									
	Min Feret Diameter	89.2		>	>	<									
	Aspect Ratio	18.2			>							>		<	
	Roundness	25.9				>									
	Solidity	11.3			<										

The femur and tibia both formed significantly larger and less circular (Marg. *R* ^2^ = 54.0%) pores than the rib. Increased pore size was reflected in several aspects of mean pore morphometry, including area (Marg. *R* ^2^ = 88.6%), perimeter (Marg. *R* ^2^ = 88.7%), maximum feret diameter (Marg. *R* ^2^ = 87.9%), and minimum feret diameter (Marg. *R* ^2^ = 89.2%). The tibia alone was significantly elevated over the rib in mean pore aspect ratio (Marg. *R* ^2^ = 18.2%), which the interaction contrast indicated was due to trabecularized pores alone. These irregular borders also reduced tibial mean pore solidity (Marg. *R* ^2^ = 11.3%) in respect to the rib.

### 3.4 Regional variability in pore morphometry

The femur displayed no significant regional variation in total percent porosity, pore density, or mean pore size and shape (Table 7, Tables S8 and S9). Trabecularized pore density was significantly concentrated in the anterior quadrant relative to lateral and posterior quadrants (Marg. *R* ^2^ = 86.7%).

**TABLE 7:** Significant post‐hocs for regional comparisons: Femur

Variable		Marg.*R* ^2^ (%)	Region						Type	Cortical pores						Trabecularized pores						A	L	M	P
			A‐L	A‐M	A‐P	L‐M	L‐P	M‐P	Ct. ‐ Tb.	A‐L	A‐M	A‐P	L‐M	L‐P	M‐P	A‐L	A‐M	A‐P	L‐M	L‐P	M‐P	Ct. ‐ Tb.			
Agg.	Percent Porosity	11.7							>																
	Pore Density	86.7							>							>		>				>	>	>	>
Pore Means	Area	82.4							<																
	Perimeter	84.3							<																
	Circularity	38.9							>																
	Max Feret Diameter	82.9							<																
	Min Feret Diameter	84.2							<																
	Aspect Ratio	6.2																							
	Roundness	12.2							>																
	Solidity	3.2																							

The tibia showed no significant regional variation in pore density, but did display regional patterning in percent porosity and mean pore size and shape (Table 8, Tables S10 and S11). Cortical percent porosity and pore size were concentrated anteriorly. The anterior quadrant significantly exceeded all other regions in percent cortical porosity (Marg. *R* ^2^ = 29.3%), the lateral and medial quadrants in mean cortical pore area (Marg. *R* ^2^ = 79.5%), and the medial quadrant alone in mean cortical pore minimum feret diameter (Marg. *R* ^2^ = 83.8%). Trabecularized percent porosity was concentrated both anteriorly and medially (Marg. *R* ^2^ = 29.3%). Trabecularized pore size similarly followed the pattern Anterior > Medial > Posterior > Lateral, with only the Anterior‐Medial and Medial‐Posterior comparisons remaining insignificant. This pattern included means of trabecularized pore area (Marg. *R* ^2^ = 79.5%), perimeter (Marg. *R* ^2^ = 81.1%), maximum feret diameter (Marg. *R* ^2^ = 80.3%), and minimum feret diameter (Marg. *R* ^2^ = 83.8%).

**TABLE 8:** Significant post‐hocs for regional comparisons: Tibia

Variable		Marg.*R* ^2^ (%)	Region						Type	Cortical pores						Trabecularized pores						A	L	M	P
			A‐L	A‐M	A‐P	L‐M	L‐P	M‐P	Ct. ‐ Tb.	A‐L	A‐M	A‐P	L‐M	L‐P	M‐P	A‐L	A‐M	A‐P	L‐M	L‐P	M‐P	Ct. ‐ Tb.			
Agg.	Percent Porosity	29.3	>		>	<			>	>	>	>				>		>	<		>			<	
	Pore Density	85.1							>																
Pore Means	Area	79.5	>			<			<	>	>					>		>	<	<		<	<	<	<
	Perimeter	81.1	>						<							>		>	<	<		<	<	<	<
	Circularity	44.0							>																
	Max Feret Diameter	80.3	>						<							>		>	<	<		<	<	<	<
	Min Feret Diameter	83.8	>			<			<		>					>		>	<	<		<	<	<	<
	Aspect Ratio	12.9							<																
	Roundness	19.2							>																
	Solidity	7.7																							

The rib displayed a significant cutaneous elevation in total percent porosity (Marg. *R* ^2^ = 55.7%), mean pore area (Marg. *R* ^2^ = 74.8%), and mean pore minimum feret diameter (Marg. *R* ^2^ = 79.8%), which the interaction contrast showed was due to trabecularized pores (Table 9, Tables S12 and S13). The rib also showed a significant cutaneous elevation in means of pore perimeter (Marg. *R* ^2^ = 75.1%) and max feret diameter (Marg. *R* ^2^ = 74.7%), derived from cortical and trabecularized pores in aggregate. Conversely, the pleural cortex had a significantly elevated pore density (Marg. *R* ^2^ = 91.6%), which the interaction contrast showed was due to cortical pores.

**TABLE 9:** Significant post‐hocs for regional comparisons: Rib

Variable		Marg. *R* ^2^ (%)	Region	Type	Cortical	Trabecularized	Cutaneous	Pleural
			Cut ‐ Ple	Ct. ‐ Tb.	Cut ‐ Ple	Cut ‐ Ple	Ct. ‐ Tb.	
Agg.	Percent Porosity	55.7	>	<		>	<	
	Pore Density	91.6	<	>	<		>	>
Pore Means	Area	74.8	>	<		>	<	<
	Perimeter	75.1	>	<				
	Circularity	25.6		>				
	Max Feret Diameter	74.7	>	<				
	Min Feret Diameter	79.8	>	<		>	<	<
	Aspect Ratio	7.6						
	Roundness	8.6						
	Solidity	5.6						

### 3.5 Characterizing pore type morphometry

Cortical porosity significantly predominated in the femur and tibia, while trabecularized porosity was more prevalent in the rib (Table 10). Cortical pore density significantly exceeded trabecularized pore density in all skeletal elements. Pore type size and shape were consistent across the femur, tibia, and rib. Cortical pores were significantly more circular but had a smaller size (means of pore area, perimeter, and maximum and minimum feret diameter) than trabecularized pores for all skeletal elements. Cortical pores were also significantly rounder than trabecularized pores in the femur and tibia, and had a significantly smaller mean aspect ratio in the tibia alone.

**TABLE 10:** Comparison of pore type morphometry

Variable		Cortical ‐ Trabecularized			
		All	Femur	Tibia	Rib
Agg.	Percent Porosity		>	>	<
	Pore Density	>	>	>	>
Pore Means	Area	<	<	<	<
	Perimeter	<	<	<	<
	Circularity	>	>	>	>
	Max Feret Diameter	<	<	<	<
	Min Feret Diameter	<	<	<	<
	Aspect Ratio			<	
	Roundness	>	>	>	
	Solidity				

## 4 DISCUSSION

### 4.1 Validation against manual annotation

Pore Extractor 2D is a toolkit for computer‐assisted extraction of pore spaces. This toolkit replicates manual annotation, as demonstrated in the validation sample of midshaft, midthoracic human ribs (*n* = 30). Porosity, pore density, and mean pore size were statistically comparable both between methods and observers. Pore Extractor 2D did significantly increase pore perimeter and decrease pore circularity, compared to manual annotation. This suggests that manual annotation smooths and rounds pore borders, compared to computer‐assisted pixel fitting. The toolkit uses variations in pixel brightness to precisely fit the borders between pores and external tissue.

The typical processing time for a single rib section, excluding section border clearing, was 3–4 h with manual annotation, compared to 1–1.5 h with Pore Extractor 2D. User‐optimized *Pore Extractor* and automated *Pore Analyzer* each required approximately 15 min. Manual correction in *Pore Modifier* typically required between 30 min (for a small rib with 200 pores) and 1 h (for a large rib with 400 pores). Pore Extractor 2D offers a potential time savings of 50–75% over manual annotation for experienced users.

### 4.2 Intraskeletal variation in pore morphometry

Intraskeletal and regional variation in porosity was demonstrated in a pilot sample of matched (*n* = 9) midshaft human femora, tibiae, and ribs. Removal of porosity always significantly reduced percent cortical area, regardless of pore type or skeletal element. This calculation of “bone area” has been shown to improve prediction of rib structural properties associated with fracture, although *R* ^2^ gains are marginal (Dominguez et al., 2016).

Porosity can also inform the mechanical strain patterns that influence tissue retention or resorption. No significant intraskeletal differences were observed between the femur, tibia, and rib in total percent porosity. Similarly, a previous study (Hunter & Agnew, 2016) which used an early developmental version of Pore Extractor 2D (Cole & Stout, 2015), found no significant intraskeletal differences in total percent porosity between the midshaft femur, midshaft rib, and distal radius. However, intraskeletal differences in porosity were observed in the present study after pores were subdivided into “cortical” and “trabecularized.” Cortical percent porosity was significantly elevated in the femur and tibia, compared to the rib. Trabecularized percent porosity showed the inverse pattern of significant elevation in the rib over the femur and tibia. This patterning is consistent with the relatively larger cortical area of the femur and tibia, and with the extensive trabecularization commonly observed in the rib (Dominguez & Agnew, 2016).

Total pore density was significantly elevated in the rib, compared to the femur and tibia. The interaction contrast suggested this was due to the rib's higher density of trabecularized pores. Studies of humans and quadrupeds have confirmed that the rib experiences a higher remodeling rate than limb bones, inferred from its elevated osteon population density (Cho & Stout, 2011; Fahy et al., 2017; Mulhern, 2000; Mulhern & Van Gerven, 1997; Skedros et al., 2003; Stout, 1984; Vajda et al., 1999; Wilson et al., 1998). Elevated rib remodeling has been attributed to the rib's low‐strain permissiveness to stochastic remodeling (Frost, 1989), sensitivity to metabolic stimulation of resorption (Skedros et al., 2013), a potentially earlier effective age of adult compacta (Mulhern, 2000; Mulhern & Van Gerven, 1997), and more frequent targeted remodeling of microdamage associated with respiration (Cho & Stout, 2011; Skedros et al., 2003).

Femoral and tibial pores were significantly larger and less circular than rib pores, for all pore types. The tibia additionally created pores with more irregular borders than the rib, which the interaction contrast indicated was derived from tibial trabecularized pores. This patterning may be derived from the pilot sample's bias towards older individuals. Average pore size has been shown to increase with age in the human femur, while rib pore size remains approximately the same through the lifespan (Jowsey, 1966; Takahashi et al., 1965) potentially related to the limited cortex available in ribs for remodeling events (Dominguez & Agnew, 2016).

The femur and tibia did not significantly differ in any aspect of percent porosity, pore density, or pore size and shape morphometry. Comprehensive sampling of the femoral and tibia cortex suggests that the femur and tibia have similar remodeling rates (Drapeau & Streeter, 2006), supporting the intraskeletal invariability observed in this study.

### 4.3 Regional variation in pore morphometry

Pore Extractor 2D provides summary measurements for each pore type in long bone quadrants and rib cutaneous and pleural cortices. Assessment of this regional distribution provides insight into remodeling activity associated with localized mechanical strain. Compressed regions of a cross‐section experience higher strain and are typically less porous than tensed regions, as observed in human ribs (Agnew & Stout, 2012), human femoral necks (Bell, Loveridge, Power, Garrahan, Meggitt, & Reeve, 1999; Bell, Loveridge, Power, Garrahan, Stanton, et al., 1999), and mule deer calcanei (Skedros, Bloebaum, et al., 1994).

The femur experiences complex mechanical loading, combining axial compression, bending, and torsion. Consequently, femoral regions should not be expected to display clear microstructural patterns associated with local strain (Skedros, 2012). Micro‐CT analyses have found both significant elevation of porosity in anterolateral and posterior regions (Thomas et al., 2005) and no significant regional patterning in total pore volume or mean pore diameter (Carter et al., 2014). The pilot study did not identify any regional femoral patterns in aggregate pore morphometry. Total, cortical, and trabecularized percent porosity were highest in the anterior quadrant. However, the only significant regional comparison was an anterior elevation of trabecularized pore density, relative to lateral and posterior quadrants. Collagen fiber orientation suggests that the anterolateral femur experiences more tensile strains than compressive strains (Goldman et al., 2003; Portigliatti Barbos et al., 1984). Significantly elevated remodeling in the anterolateral femur has been attributed to targeted remodeling of this tension and shear damage (Gocha & Agnew, 2016). This increased remodeling could explain the localized anterior elevation in trabecularized pore density.

This pilot study did find significant regional patterning in the tibia, corresponding to localized mechanical strain. Cortical percent porosity and pore size were elevated anteriorly. Trabecularized percent porosity and pore size were elevated both anteriorly and medially. Gait simulation in strain‐gaged cadaveric tibiae suggests that the anterior crest experiences peak tensile strains, while the medial region experiences relatively low strains. (Peterman et al., 2001). The anterior concentration of porosity could reflect these locally high strains. The additional medial concentration of trabecularized porosity could suggest permissiveness to disuse‐mode remodeling, stimulated by locally low strains. Disuse‐mode remodeling favors resorption and preferentially occurs at the endosteum, as is characteristic of trabecularized pores (Hughes et al., 2020).

The sixth rib displayed a significant cutaneous elevation in perc porosity and pore size, and a significant cutaneous depression in pore density. Previous studies have confirmed high cutaneous percent porosity in human ribs across the lifespan, from pediatric to elderly ribs (Agnew et al., 2013; Agnew & Stout, 2012; Dominguez & Agnew, 2016). The localized strain mode that causes this cutaneous localization of percent porosity is unclear. Inspiration bends the lower ribs (primarily 7th–10th) inwards, along their curvature, potentially placing the cutaneous cortex in lower‐strain tension and the pleural cortex in higher‐strain compression. The upper ribs (primarily 2nd–6th) bend outwards, against their curvature, potentially reversing this strain patterning (Agnew et al., 2013; Dominguez & Agnew, 2016; Moore et al., 2013). Given the placement of the sixth rib at the intersection of these movements, its high cutaneous porosity could be attributed to high strain (targeted remodeling) or to low strain (disuse‐related or stochastic remodeling). This pilot study indicates that increased cutaneous porosity and pore size are derived from trabecularized pores. These results support the explanation that high cutaneous porosity is derived from disuse‐mode remodeling (low strain), which favors trabecularization through pore expansion and convergence (Hughes et al., 2020).

### 4.4 Trabecularized pore morphometry reflects pore erosion

Skeletal biologists have long recognized that porosity increases towards the endosteum, where mechanical strain is low due to proximity to the neutral axis (Atkinson, 1965; Jowsey, 1960; Martin et al., 1980; Thomas et al., 2005; Zebaze et al., 2010). Quantifying this trabecularized porosity is important for accurately characterizing age‐associated bone loss. For example, in the human distal radius, bone loss between ages 50 and 80 is primarily derived from trabecularization (47%), followed by trabecular thinning (32%) and cortical pore formation (21%) (Zebaze et al., 2010).

Cortical pores were significantly smaller and more circular than trabecularized pores in all elements, and were significantly less elongated in the femur and tibia. A histological analysis of the human fibula similarly found that endosteal pores significantly exceeded periosteal pores in diameter, despite equivalent pore densities. Large‐diameter (>100 μm) endosteal pores were almost exclusively derived from the remodeling and erosion of existing pores (97.5%), rather than the creation of new pores (Andreasen et al., 2020). This formation through convergence could explain the trabecularized pore shape distortion observed in the pilot study.

## 5 CONCLUSION

Cortical porosity is a key metric for characterizing bone loss in biomedical, biomechanical, and anthropological studies. Porosity quantification has been limited by the time required for tedious manual annotation of discrete pore spaces. Pore Extractor 2D is a free, open‐source toolkit for semi‐automated extraction, expedited manual modification, and fully automated analysis of pore morphometry on histological images. Pore Extractor 2D measurements of percent porosity, pore density, and pore size were statistically equivalent to manual modification. The toolkit also facilitated close pixel fitting to pore borders. A pilot study of intraskeletal and regional variation demonstrated that porosity analysis can inform localized strain patterning.

## AUTHOR CONTRIBUTIONS

**Mary E. Cole:** Conceptualization (lead); data curation (lead); formal analysis (lead); investigation (lead); methodology (lead); project administration (lead); software (lead); validation (lead); visualization (lead); writing – original draft (lead); writing – review and editing (equal). **Sam D. Stout:** Conceptualization (supporting); data curation (supporting); funding acquisition (lead); investigation (supporting); methodology (supporting); project administration (supporting); resources (equal); supervision (equal); writing – review and editing (equal). **Victoria M. Dominguez:** Data curation (supporting); formal analysis (supporting); investigation (supporting); methodology (supporting); software (supporting); validation (equal); writing – review and editing (equal). **Amanda M. Agnew:** Conceptualization (supporting); data curation (supporting); funding acquisition (supporting); investigation (supporting); methodology (supporting); project administration (supporting); resources (equal); supervision (equal); writing – review and editing (equal).

## CONFLICT OF INTEREST

The authors declare no potential conflict of interest.

## Supporting information


**Appendix S1** Supporting Information

## Data Availability

The data that support the findings of this study are available from the corresponding author upon reasonable request.
