# Supplementary material for: Pore Extractor 2D: An ImageJ toolkit for quantifying cortical pore morphometry on histological bone images, with application to intraskeletal and regional patterning
Source: Am J Biol Anthropol. 2022 Sep 14;179(3):365–85. doi: 10.1002/ajpa.24618 (PMC9826150; doi:10.1002/ajpa.24618)
Supplement: Supplementary file 1 — Appendix S1 Supporting Information [file AJPA-179-365-s001.zip › AJPA_24618_Cole_Supplement_Final.pdf]

Table of Contents

Pore Extractor 2D Installation Instructions 2
Pore Extractor 2D Session Startup Instructions 6
Pore Extractor 2D Recommended Starter Settings 7
SUPPLEMENTAL TABLE 1. Pore Modifier Keyboard Shortcuts 13
SUPPLEMENTAL TABLE 2: Linear Mixed Model for Cortical Area vs. Bone Area 14
SUPPLEMENTAL TABLE 3: Post-Hoc Analysis for Cortical Area vs. Bone Area 14
SUPPLEMENTAL TABLE 4: Linear Mixed Model for Cross-Sectional Geometry 15
SUPPLEMENTAL TABLE 5: Post-Hoc Analysis for Cross-Sectional Geometry 18
SUPPLEMENTAL TABLE 6: Linear Mixed Model for Intraskeletal Comparisons 19
SUPPLEMENTAL TABLE 7: Post-Hoc Analysis for Intraskeletal Comparisons 23
SUPPLEMENTAL TABLE 8: Linear Mixed Model for Regional Comparisons: Femur 27
SUPPLEMENTAL TABLE 9: Post-Hoc Analysis for Regional Comparisons: Femur 31
SUPPLEMENTAL TABLE 10: Linear Mixed Model for Regional Comparisons: Tibia 33
SUPPLEMENTAL TABLE 11: Post-Hoc Analysis for Regional Comparisons: Tibia 37
SUPPLEMENTAL TABLE 12: Linear Mixed Model for Regional Comparisons: Rib 41
SUPPLEMENTAL TABLE 13: Post-Hoc Analysis for Regional Comparisons: Rib 44

Pore Extractor 2D Installation Instructions

Sample images and tutorials for Pore Extractor 2D are available on the site:
https://github.com/MaryECole/PoreExtractor2D

Pore Extractor 2D requires ImageJ FIJI version **1.53g** or later distribution.
Downloads and system requirements can be found at: https://imagej.net/Fiji/Downloads

Unzip the download and extract the Fiji.app folder. If installing on Windows, the folder should be stored somewhere on the user space (e.g. Desktop, Documents), rather than in Program Files, to facilitate write permissions.

ImageJ can be opened by clicking on the application within the Fiji.app folder.

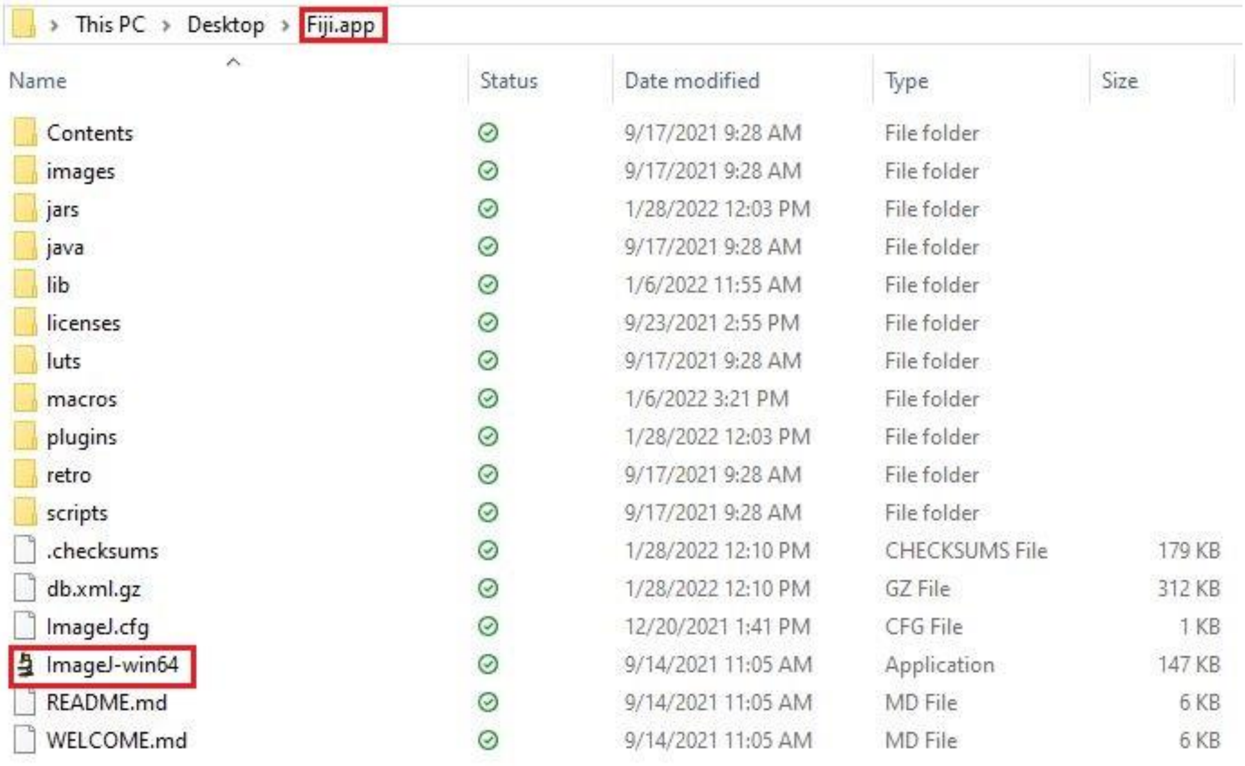

The current version of Pore Extractor 2D should be installed from its ImageJ update site. This update site also automatically installs the Pore Analyzer tool dependencies BoneJ (Doube et al., 2010) and BioVoxxel Toolbox (Brocher, 2015).

The user will be prompted to close and restart ImageJ several times during this installation process.

1. Open the ImageJ application and navigate to **Help → Update.** If the ImageJ installation is new, several default updates may install, the user will be prompted to **Apply Changes**, close ImageJ, re-open ImageJ, and call Help → Update again.

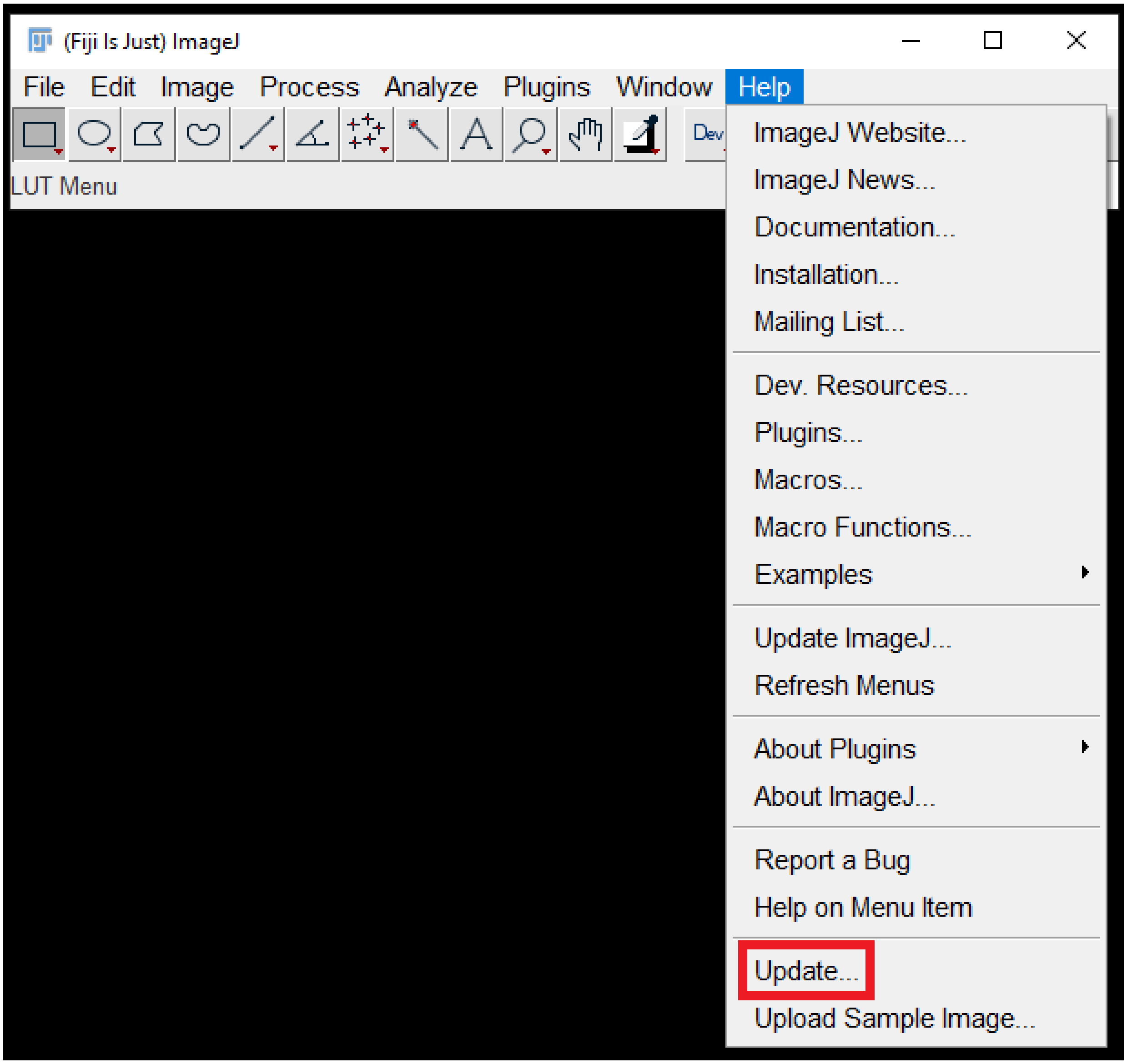

2. Select **Manage update sites,** then **Add update site**, and input the following information:

Name: PoreExtractor2D
URL: https://sites.imagej.net/PoreExtractor2D/
Host: webdav:MECole

Then select **Close** to be returned to the **Manage update sites** window.

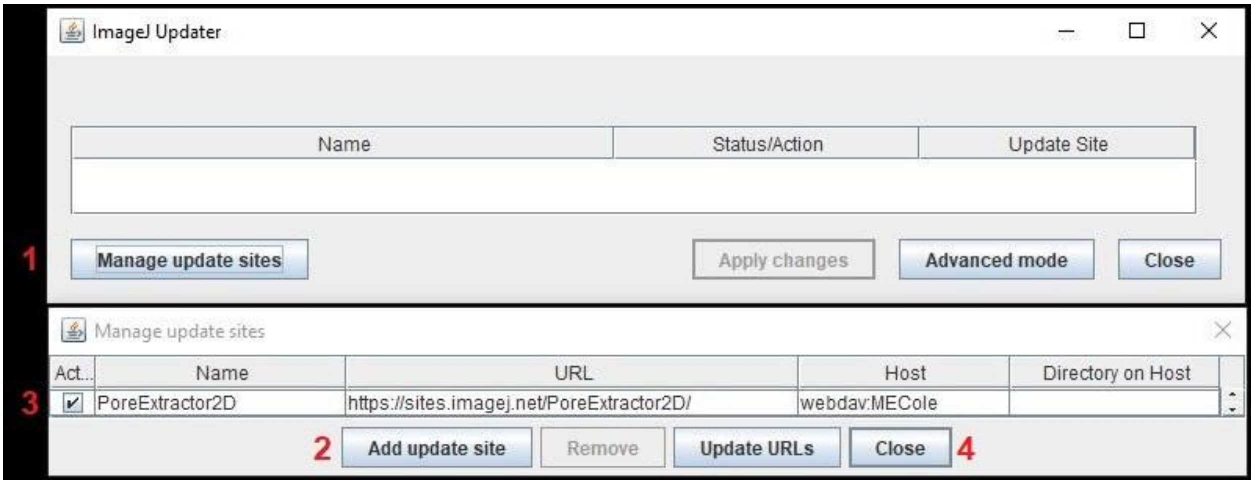

3. Pore Extractor 2D dependencies will populate in the **Manage update sites** window. Note that only "Pore_Extractor_2D.ijm" will populate if the user previously installed BoneJ and Biovoxxel dependencies. Select **Apply changes**. The user will be prompted to restart ImageJ.

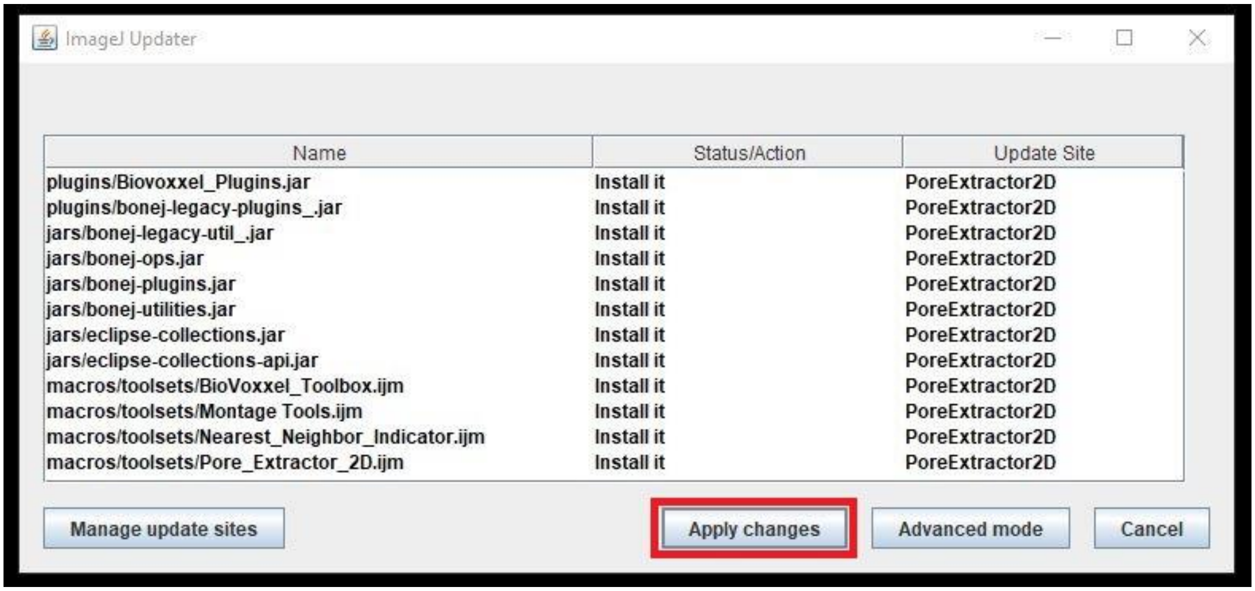

After download, the “Pore_Extractor_2D.ijm” file will appear in the Fiji.app subfolder **macros → toolsets.**

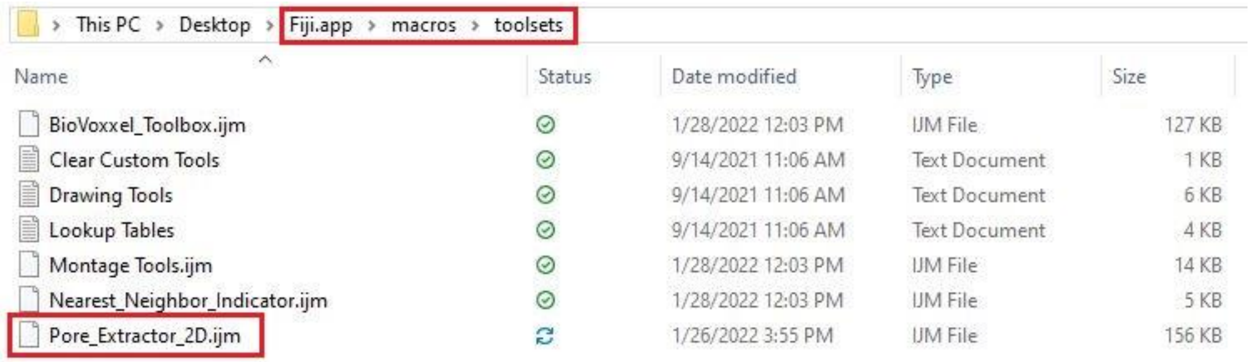

If the user encounters difficulties downloading from the update site, the “Pore_Extractor_2D.ijm” file attached with this publication’s supplement can be manually copied into the toolset subfolder. In this case, the user would also need to install BoneJ and BioVoxxel Toolbox individually from the ImageJ Update menu.

4. Navigate to **Help → Update ImageJ** and update to the latest version (**1.53g or later**). ImageJ will close automatically after this update and will need to be restarted.

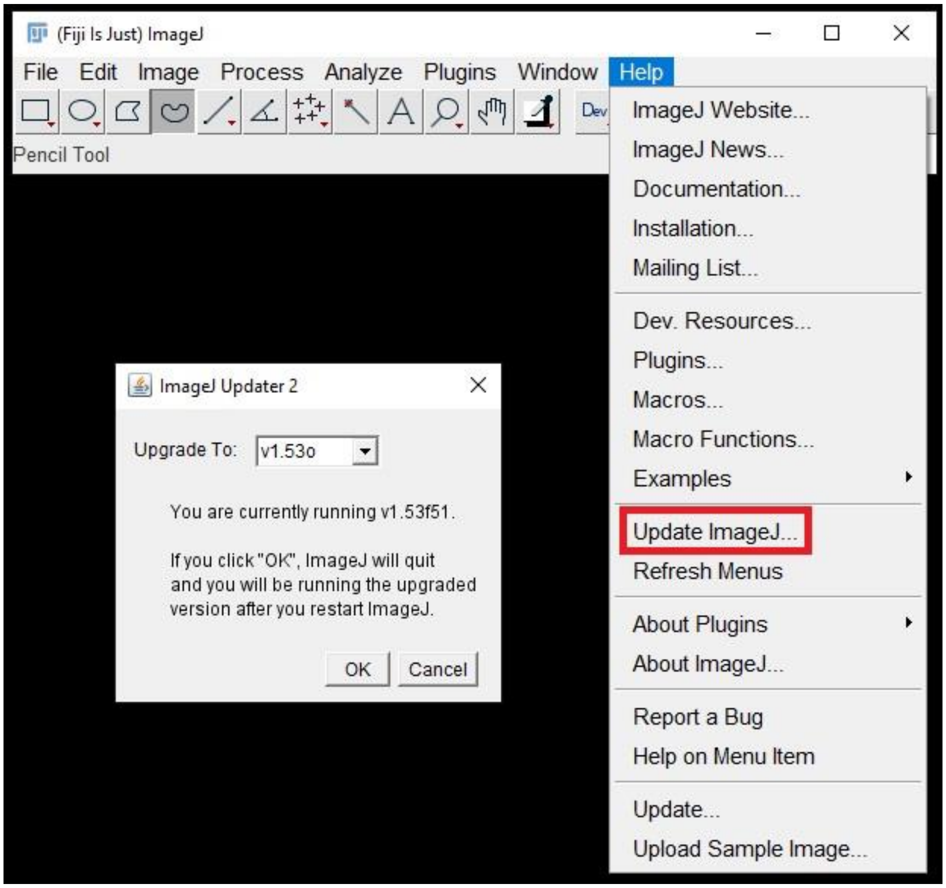

Pore Extractor 2D Session Startup Instructions

Pore Extractor 2D must be installed each time a new session of ImageJ is opened.

1. Select “Pore_Extractor_2D” from the “More Tools” (**>>**) dropdown menu, and the toolkit icon will appear in the ImageJ toolbar.

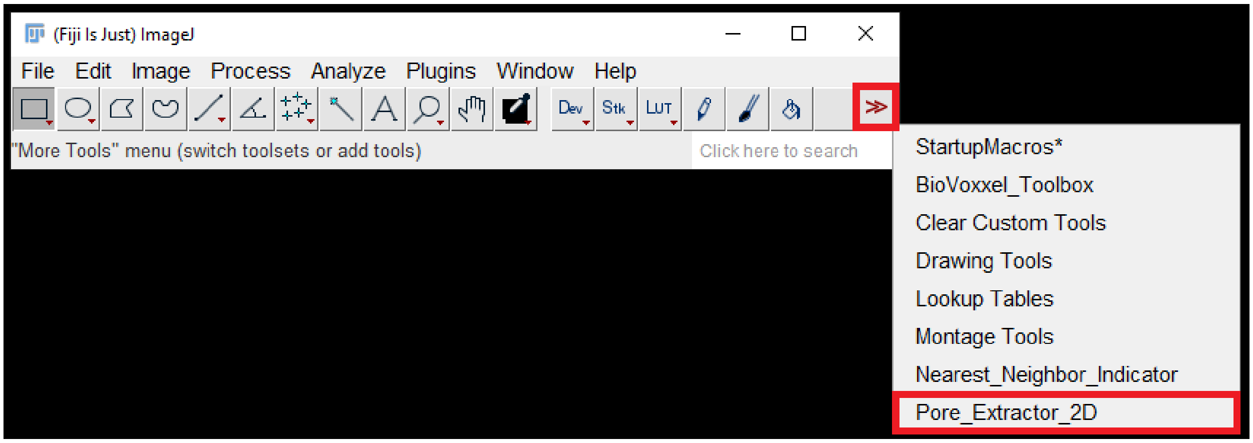

2. Click the Pore Extractor 2D toolkit icon to select a macro from its dropdown menu.

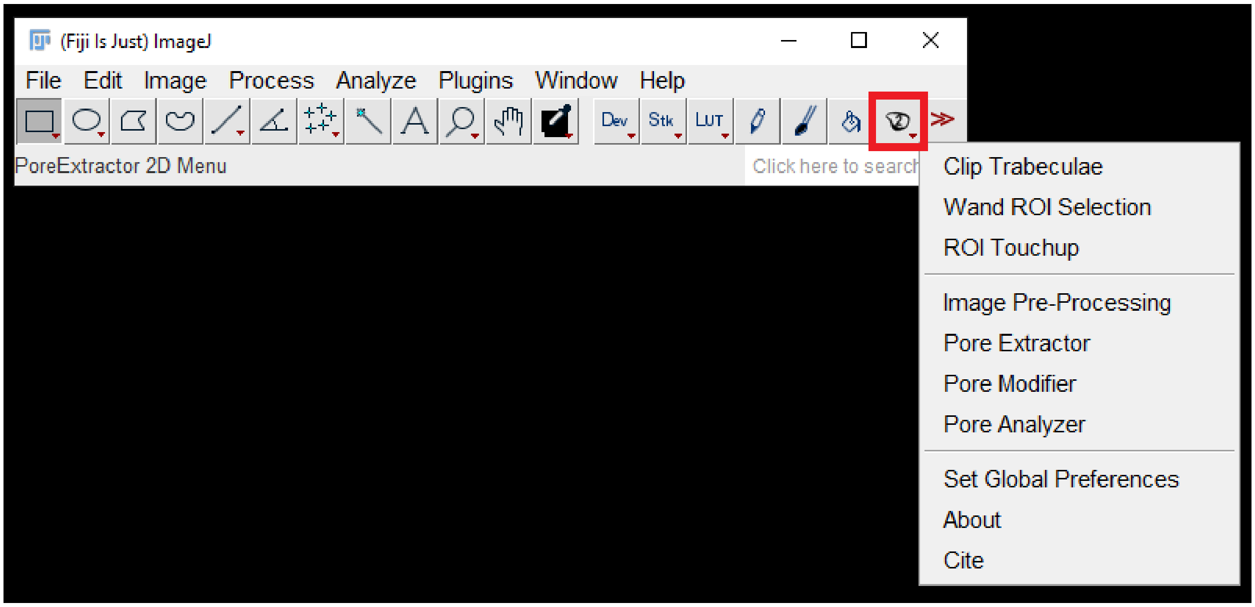

Pore Extractor 2D Recommended Starter Settings

Pore Extractor 2D requires user-selected settings for *Image Pre-Processing* and *Pore Extractor* macros. The following settings are recommended as a starting point for this optimization. Users are encouraged to test and modify these settings to optimize cortical pore identification for their own samples.

Image Pre-Processing Recommended Settings

1. **Enhance Global Contrast** (Normalize) – 0.3% Saturation (default)
2. **Subtract Background** (Rolling Ball) – 50.0 px (default)
3. **Gaussian Blur** – 2.00 px (default)
    a. Recommended for samples with 100x or greater magnification, as 40x magnification samples already have some inherent blurring of pore borders
    b. Also recommended for "noisy" images with surface debris, tissue discoloration or bleaching, or bubbling and partitioning inside large pore spaces
4. **Save Image Channels** (All)
    a. Recommended for stained samples. Select the grayscale color channel that minimizes the intensity of tissue staining for further processing in *Pore Extractor*
    b. If resulting grayscale images are faint, run Image Pre-Processing without the Subtract Background option.

Pore Extractor Recommended Workflow

Expect Proximity, Not Perfection

Due to the color heterogeneity of histological bone tissue, *Pore Extractor* will not perfectly select all pore spaces. You will always need to run *Pore Modifier* to remove some incorrectly selected tissue, and to add or alter some pore selections.

Choose *Pore Extractor* settings to select **small-to-medium** sized pores, which are the majority of cortical pores in a typical section. These pores tend to have a uniformly colored lumen and complete border.

- Very small pores tend to be removed by the smoothing cycle(s) and minimum pore size filters necessary to exclude tissue noise.
- Large "trabecularized" pores near the endosteum tend to be excluded by the circularity filter necessary to exclude tissue noise, either because they have incomplete borders that cannot be filled, they are sub-segmented by internal bubbling or debris, or they are irregularly shaped.
- It is quicker to add these pores back in *Pore Modifier* than it is to delete a large amount of selected tissue noise. Aim to minimize tissue noise over selecting every pore.

For a good-quality histological section, you should aim to select about **60 - 75%** of all pores with *Pore Extractor*.

Thresholding Recommended Workflow:

1. Follow the load dialog prompts.
   a. Load the cleared and (optionally) preprocessed brightfield image
   b. Set the image scale in pixels/mm

2. The *Thresholding* Method popup dialog will appear. Select Try *All*, which will cycle through each thresholding option in turn.
   a. Keep the default Auto Local Phansalkar radius of 15 pixels.
   b. Click OK.

3. *Pore Lumen Extraction* will prompt user interaction.
   a. Move the **top** slider to the **right** in the popup Threshold window, until light pore **spaces** are selected in **red**.
   b. Minimize speckles of red inside the bone tissue (outside of the pores). Clusters of speckles should not exceed the size of pore spaces. A scattered red "television static" appearance inside the tissue is normal.
   c. If you have moved the top slider all the way to the right (255) and large clusters of red persist inside the tissue, change the dialog option to "Do Not Use Pore Lumen Threshold." This is common if you are using a grayscale image.
   d. **Do not click Apply on the Threshold popup!** Instead, click **OK** in *the Pore Lumen Extraction* popup dialog to continue.

4. *Pore Border Extraction* will prompt user interaction.
   a. Move the **bottom** slider to the **left** in the popup Threshold window, until dark pore **borders** are selected in **red**.
   b. Minimize speckles of red inside the bone tissue (outside of the pores). Clusters of speckles should not exceed the size of pore spaces. A scattered red "television static" appearance inside the tissue is normal.
   c. If you have moved the bottom slider all the way to the left (0) and large clusters of red persist inside the tissue, change the dialog option to "Do Not Use Pore Border Threshold." This is common if the tissue has dark discoloration.
   d. **Do not click Apply on the Threshold popup!** Instead, click **OK** in *the Pore Border Extraction* popup dialog to continue.

5. *Auto Local Thresholding* will run automatically.
   a. All image windows will disappear while this function runs in the background. This may take several minutes, depending on image size and computer processing power. A pop-up progress dialog will alert you that the function is still running.

6. All thresholding outputs will appear in tiled windows. Select your choice from the drop-down menu, then click OK.
    a. Manual Pore Lumens and/or Manual Pore Borders will not appear if you selected the option to not use that threshold
    b. For better visibility, you can use the *Zoom* tool (keyboard shortcut [4]) to right-click and zoom in, and the *Scrolling* tool (keyboard shortcut [5] or hold [Space]) to grab and drag the image while zoomed.
    c. Choose the thresholding output that maximizes **filled, isolated pores** while minimizing large clumps of white inside the bone tissue
    d. For tissue with minimal debris or discoloration, *Auto_Local_Phansalkar* or *All_Thresholds_Combined* is typically the best option
    e. For tissue with more surface debris or discoloration, one of the *Manual* outputs is typically the best option.

Morphological Modification Recommended Workflow:

Following thresholding, the *Morphological Modification* popup dialog will appear. You will also see displayed 1) the brightfield cross-section, titled with its image name and 2) the thresholded pore image, titled as *Current_Pore_Modification*.

This is a repeating modification loop that you can return to until you are satisfied with final pore selections. You can select multiple modifications to run at once, by checking multiple boxes. However, it is recommended to test out a single modification, view the results, and accept or reject that change, before testing and adding the next modification.

Before or during modification, you can visualize pore selection from the current image modification by selecting "Preview Selected Workflow Option(s)" and clicking OK.

If, at any point, you want to discard morphological change(s) you have already accepted, select "Revert to Original Thresholded Image" and click OK.

A. Close and Fill Pore Borders

Try the following steps if many pores appear as incomplete, unfilled "rings." **If most small to medium sized pores are already filled in, skip to *B: Smooth Pore Borders.***

1. In the *Morphological Modification* popup dialog, check the box for "Close and Fill Pores (1 pixel)".
    a. Do not check any other boxes.
    b. Select the preview option "Preview Selected Workflow Option(s)."
    c. Click OK and wait for the modification to run.

2. The output of your Close and Fill Pores modification will appear as *Preview_Pores.*
    a. The *Pore Filter Settings* popup dialog will appear.

3. **Did large regions of non-pore tissue merge into white patches?** Reject Close and Fill, then proceed to smoothing.
    a. In the *Pore Filter Settings* popup dialog, select “Skip Pore Display” and click OK.
    b. The *Accept Morphological Changes* popup dialog will appear. Select “Discard Change” and click OK.
    c. You will be returned to the *Morphological Modification* popup dialog.
    d. The *Current_Pore_Modification* image will revert to its previous state.
    e. Continue to ***B: Smooth Pore Borders.***

4. **Are there still many pores that appear as incomplete, unfilled “rings”?** Reject Close and Fill and re-run with a higher pixel value.
    a. In the *Pore Filter Settings* popup dialog, select “Skip Pore Display” and click OK.
    b. The *Accept Morphological Changes* popup dialog will appear. Select “Discard Change” and click OK.
    c. You will be returned to the *Morphological Modification* popup dialog.
    d. The *Current_Pore_Modification* image will revert to its previous state.
    e. Repeat ***A. Close and Fill Pore Borders*** with the “Close and Fill Pores” setting increased to 2 pixels.

5. **Are pores suitably filled?** Accept Close and Fill, then proceed to smoothing.
    a. In the *Pore Filter Settings* popup dialog, select “Skip Pore Display” and click OK.
    b. The *Accept Morphological Changes* popup will appear. Select “Accept Change” and click OK.
    c. You will be returned to the *Morphological Modification* popup dialog.
    d. The *Current_Pore_Modification* image will now match the closed and filled *Preview_Pores* image.
    e. Continue to ***B: Smooth Pore Borders.***

B. Smooth Pore Borders

In almost all cases, smoothing is required to reduce the pixelated noise of pore borders. Smoothing can also cut off extraneous connections to tissue caused by thresholding or Close and Fill operations. This operation will remove pore border projections that are a smaller diameter than the selected smoothing value (typically 2 – 10 pixels).

1. In the *Morphological Modification* popup dialog, check the box for “Smooth Pore Borders.” Do not check any other boxes.
    a. If you did **not** accept a Close and Fill operation: Enter 5 pixels.
    b. If you **did** accept a Close and Fill operation: Enter 7 pixels.
    c. Select the preview option “Preview Selected Workflow Option(s).”
    d. Click OK and wait for the modification to run.

2. The output of your Close and Fill Pores modification will appear as *Preview_Pores.*
    a. The *Pore Filter Settings* popup dialog will appear.

3. In the *Pore Filter Settings* popup dialog, keep all default settings and click OK.
    a. The *Confirm Pore Selection* popup dialog will appear.
    b. Pores that pass the default minimum size (> 300 μm^2) and circularity (> 0.3) thresholds will display as cyan-colored overlays on the brightfield image and on the modified *Preview_Pores* image.

4. **Is it difficult to see the pore selections on the brightfield image?** Change the selection color to better contrast with your brightfield image.
    a. In the *Confirm Pore Selection* popup dialog, select "No, Adjust Pore Filter or Color" and click OK.
    b. The *Pore Filter Settings* popup dialog will appear.
    c. Under "Change Pore Color," select a color that contrasts with your brightfield image. Red or green are good choices for an unstained bone section. Cyan or green is a good contrasting color for a section stained pink with basic fuschin.
    d. You can also switch the pore selection type from "Outline" to "Filled." A filled selection is more visible than an outline, but it can also obscure the true boundaries of the pore.
    e. Click OK to apply these changes to the pore color and selection type.
    f. For better visibility, you can use the *Zoom* tool (keyboard shortcut [4]) to right-click and zoom in, and the *Scrolling* tool (keyboard shortcut [5] or hold [Space]) to grab and drag the image while zoomed.
    g. Repeat this step until you can see the pore selections on the brightfield image. Then proceed to the next questions.

5. **Do pore borders still appear too pixelated, jagged, or connected to surrounding tissue?** Increase the smoothing threshold.
    a. In the *Confirm Pore Selection* popup dialog, select "No, Adjust Morphometry" and click OK.
    b. The *Accept Morphological Changes* popup dialog will appear. Select "Discard Change" and click OK.
    c. You will be returned to the *Morphological Modification* popup dialog. The "Smooth Pore Borders" pixel value will be set to the cycle you just tested.
    d. The *Current_Pore_Modification* image will revert to its previous state.
    e. Repeat ***B. Smooth Pore Borders*** with the "Smooth Pore Borders" value **increased** by one or more pixels.

6. **Do many regions of bone tissue appear as small, round blobs that are approximately the same size as the pores?** Decrease the smoothing threshold.
    a. In the *Confirm Pore Selection* popup dialog, select "No, Adjust Morphometry" and click OK.
    b. The *Accept Morphological Changes* popup dialog will appear. Select "Discard Change" and click OK.
    c. You will be returned to the *Morphological Modification* popup dialog. The "Smooth Pore Borders" pixel value will be set to the cycle you just tested.
    d. The *Current_Pore_Modification* image will revert to its previous state.
    e. Repeat ***B. Smooth Pore Borders*** with the "Smooth Pore Borders" value **decreased** by one or more pixels.

7. **Are pore borders suitably smoothed, but A) too much tissue is also selected or B) too many small pores are excluded?** Modify pore size and/or circularity thresholds.
    a. In the *Confirm Pore Selection* popup dialog, select "No, Adjust Pore Filter or Color" and click OK.
    b. The *Pore Filter Settings* popup dialog will appear.
    c. **Increase** pore size and/or circularity (range: 0 to 1) to exclude more tissue
    d. Or **Decrease** pore size and/or circularity (range: 0 to 1) to include more pores.
    e. You will likely need to repeat this cycle multiple times to fine-tune your selection.
    f. Once you are satisfied, in the *Confirm Pore Selection* popup dialog, select "Yes, Save ROIs and Exit Macro" and click OK. The macro will export the final pore ROI set.

SUPPLEMENTAL TABLE 1. Pore Modifier Keyboard Shortcuts

Shortcut	Tool Name	Tool Function
**[Ctrl+Shift+E]**	Clear Selection Brush Restore Deleted ROI	Remove selection brush modifications before the selected ROI is updated, or immediately restore a deleted ROI.
**[Backspace]**	Delete Selected ROI	Delete the selected pore ROI from the ROI manager.
**[t]**	Add Selection as ROI	Add a newly selected pore ROI to the ROI manager.
**[1]**	Wand Tool	Select the Wand tool to click and select connected pixels within the same pixel brightness tolerance range.
**[2]**	Adjust Wand Tool Tolerance	Open the Wand tool tolerance popup.
**[3]**	Freehand Selection Tool	Select the freehand selection tool, so that pores can be manually outlined with a mouse or stylus.
**[4]**	Zoom Tool	Select the Zoom tool and click on the image to zoom in (mouse left-click) or out (mouse right-click).
**[+/-]**	Zoom Without Switching Tools	Use the **[+]** key to zoom in on the mouse cursor, or the **[-]** key to zoom out from the mouse cursor, without switching tools.
**[5]**	Scrolling Tool	Select the Scrolling tool to click and drag the image to move around while zoomed.
**[Space]**	Scroll Without Switching Tools	Hold **[Space]** to click and drag the image while zoomed, without switching tools.
**[6]**	Selection Brush Tool	Activate the selection brush tool for modifying existing pore ROIs to expand the selected ROI with **[Shift]** or trim the ROI with **[Alt]**
**[8]**	Update ROI After Selection Brush Modification	Save changes to an ROI made with the Selection Brush Tool.
**[F1]**	Toggle ROI Labels On	Toggle ROI selections **on** and ROI labels **on**, which is required to select an incorrect ROI label for deletion or modification.
**[F2]**	Toggle ROI Labels Off	Toggle ROI selections **on** and ROI labels **off** for image visibility.
**[F3]**	Toggle ROIs Off	Toggle ROI selections **off** and ROI labels **off** for image visibility.
**[F4]**	Reset Wand Tool Tolerance to Zero	Reset the Wand Tool tolerance to 0 pixels, such that only pixels of the same grayscale value will be selected adjacent to a clicked pixel.
**[F5]**	Split ROI After Selection Brush Division	To split apart pores that are joined in a single ROI, click the ROI label, switch to the Selection Brush **[6]**, and hold down **[Alt]** to draw along the split line. Then split **[F5]** into two or more separate ROIs.
**[F6]**	Fill ROI After Selection Brush Expansion	To expand a partial ROI to reach the pore border, click the ROI label, switch to the Selection Brush **[6]**, and hold down **[Shift]** to draw along the missing rim. Then fill **[F6]** the pore to this drawn rim.
**[F7]**	Merge Adjacent ROIs	To merge two adjacent ROIs, use **[F7]** then click the labels of the two ROIs when prompted. Drag the mouse to connect the two ROI borders.
**[F8]**	Decrease Selection Brush Tool Size	Decrease the selection brush diameter by 5 pixels each time the key is pressed. The new diameter is displayed in the ImageJ toolbar.
**[F9]**	Increase Selection Brush Tool Size	Increase the selection brush diameter by 5 pixels each time the key is pressed. The new diameter is displayed in the ImageJ toolbar.
**[F10]**	Revert Selected ROI After Update, Split, Fill, or Merge	When an existing ROI is updated, split, filled, merged, the original ROI is stored in the output subfolder *Temp ROIs*. To restore the original ROI, click the label of the modified ROI, and use **[F10]** to revert. For split ROIs, any of the multiple ROI outputs can be selected to revert.
**[F11]**	Save New Version of ROI Set	Saves a new copy of the ROI set to the output folder each time key is pressed. Manually modified ROIs update to the user-selected color.

SUPPLEMENTAL TABLE 2: Linear Mixed Model for Cortical Area vs. Bone Area

**Cortical Area Correction Method Lambda = 2.275**							
Fixed Factor	***Estimate***	***Std. Error***	***CI (2.5%)***	***CI (97.5%)***	***t***	***p-value***	***p-value MCMC***
Intercept	9130	976	7300	11000	9.35	0.138	0.957
%Ct.Ar - %B.Ar Total	1890	287	1330	2430	6.59	***< 0.001***	***< 0.001***
%Ct.Ar - %B.Ar Ct.	-146	287	-790	438	-0.508	0.612	0.611
%Ct.Ar - %B.Ar Tb.	-1690	287	-2280	-1070	-5.91	***< 0.001***	***< 0.001***
Femur - Tibia	1680	234	1250	2110	7.19	***< 0.001***	***< 0.001***
Femur - Rib	4350	234	3920	4810	18.6	***< 0.001***	***< 0.001***
%Ct.Ar - %B.Ar Total : Femur - Tibia	261	405	-540	1130	0.645	0.521	0.513
%Ct.Ar - %B.Ar Ct. : Femur – Tibia	-296	405	-1090	514	-0.73	0.467	0.466
%Ct.Ar - %B.Ar Tb. : Femur – Tibia	-265	405	-1030	438	-0.655	0.514	0.509
%Ct.Ar - %B.Ar Total : Femur - Rib	639	405	-137	1390	1.58	0.119	0.122
%Ct.Ar - %B.Ar Ct. : Femur – Rib	-241	405	-1010	603	-0.594	0.554	0.543
%Ct.Ar - %B.Ar Tb. : Femur - Rib	-598	405	-1390	197	-1.48	0.144	0.141
Random Factor	***Estimate***	***Std. Error***	***CI (2.5%)***	***CI (97.5%)***	***ICC (%)***	***p-value***	***Model Fit***
Individual	7580000	2750	1210	4410	71	**< 0.001**	R^2 M = 0.668
Age	0	0.137	0.0004	0.415	0	1	R^2 C = 0.908
Sex	149000	386	0	3230	1.4	0.96	AIC = 1810
Residual	2960000	1720	1450	1970	27.7		BIC = 1850

SUPPLEMENTAL TABLE 3: Post-Hoc Analysis for Cortical Area vs. Bone Area

**Cortical Area Correction Method**				
Factor	***Comparison***	***p-value***	***Cohen's D***	***Effect Size***
Method	%Ct.Ar > %B.Ar Total	***< 0.001***	2.08	Large
Method	%Ct.Ar > %B.Ar Ct.	***< 0.001***	1.18	Large
Method	%Ct.Ar > %B.Ar Tb.	***< 0.001***	1.13	Large
Method	%B.Ar Ct. > %B.Ar Total	***0.00733***	0.9	Large
Method	%B.Ar Ct. < %B.Ar Tb.	0.997	0.0555	Small
Method	%B.Ar Total < %B.Ar Tb.	***0.00387***	0.956	Large
Bone	Femur < Tibia	***< 0.001***	1.55	Large
Bone	Femur > Rib	***< 0.001***	4.49	Large
Bone	Tibia > Rib	***< 0.001***	6.04	Large

SUPPLEMENTAL TABLE 4: Linear Mixed Model for Cross-Sectional Geometry

**Total Area (mm^2) Lambda = None**							
Fixed Factor	***Estimate***	***Std. Error***	***CI (2.5%)***	***CI (97.5%)***	***t***	***p-value***	***p-value MCMC***
Intercept	397	40.9	321	478	9.71	0.0668	0.925
Femur-Tibia	254	14.8	229	289	17.2	***< 0.001***	***< 0.001***
Femur-Rib	86.9	14.8	60.1	115	5.88	***< 0.001***	***< 0.001***
Random Factor	***Estimate***	***Std. Error***	***CI (2.5%)***	***CI (97.5%)***	***ICC (%)***	***p-value***	***Model Fit***
Individual	1120	33.5	0	59.5	16.2	0.24	R^2 M = 0.904
Age	0	0	0	62.3	0	1	R^2 C = 0.959
Sex	2830	53.2	0	127	41	0.132	AIC = 291
Residual	2940	54.3	35.4	70.8	42.7		BIC = 300
**Endosteal Area (mm^2) Lambda = None**							
Fixed Factor	***Estimate***	***Std. Error***	***CI (2.5%)***	***CI (97.5%)***	***t***	***p-value***	***p-value MCMC***
Intercept	138	27.4	81.9	186	5.03	0.13	0.956
Femur-Tibia	97.7	10.2	77.7	117	9.56	***< 0.001***	***< 0.001***
Femur-Rib	4.94	10.2	-13.3	26.4	0.484	0.635	0.622
Random Factor	***Estimate***	***Std. Error***	***CI (2.5%)***	***CI (97.5%)***	***ICC (%)***	***p-value***	***Model Fit***
Individual	351	18.7	0	41.8	10.2	0.668	R^2 M = 0.67
Age	502	22.4	0	51	14.6	0.723	R^2 C = 0.864
Sex	1170	34.2	0	87.9	34.1	0.265	AIC = 274
Residual	1410	37.5	23.9	47.9	41.1		BIC = 384
**Cortical Area (mm^2) Lambda = None**							
Fixed Factor	***Estimate***	***Std. Error***	***CI (2.5%)***	***CI (97.5%)***	***t***	***p-value***	***p-value MCMC***
Intercept	262	15.3	231	293	17.1	***< 0.001***	0.977
Femur-Tibia	157	11.3	134	178	13.8	***< 0.001***	***< 0.001***
Femur-Rib	81.9	11.3	61.7	106	7.24	***< 0.001***	***< 0.001***
Random Factor	***Estimate***	***Std. Error***	***CI (2.5%)***	***CI (97.5%)***	***ICC (%)***	***p-value***	***Model Fit***
Individual	1540	39.2	0.000053	66.7	47.1	***0.0277***	R^2 M = 0.903
Age	0	0.00042	0.000039	0.00085	0	1	R^2 C = 0.949
Sex	0	0	0	47.2	0	1	AIC = 280
Residual	1730	41.6	26.5	54.6	52.9		BIC = 289
**% Endosteal Area Lambda = None**							
Fixed Factor	***Estimate***	***Std. Error***	***CI (2.5%)***	***CI (97.5%)***	***t***	***p-value***	***p-value MCMC***
Intercept	43.8	3.19	37.7	50	13.7	***< 0.001***	0.942
Femur-Tibia	-7.13	1.85	-10.8	-3.55	-3.86	***0.00138***	***0.0014***
Femur-Rib	-13.6	1.85	-16.9	-9.68	-7.34	***< 0.001***	***< 0.001***
Random Factor	***Estimate***	***Std. Error***	***CI (2.5%)***	***CI (97.5%)***	***ICC (%)***	***p-value***	***Model Fit***
Individual	76.5	8.75	3.75	14.7	62.4	***0.0162***	R^2 M = 0.652
Age	0	0.00037	0.00002	0.00082	0	1	R^2 C = 0.869
Sex	0	0.00009	0.000053	0.00015	0	1	AIC = 197
Residual	46.1	6.79	4.33	8.96	37.6		BIC = 206

**% Cortical Area Lambda = None**							
Fixed Factor	***Estimate***	***Std. Error***	***CI (2.5%)***	***CI (97.5%)***	***t***	***p-value***	***p-value MCMC***
Intercept	56.2	3.19	50.2	62.5	17.6	***< 0.001***	0.986
Femur-Tibia	7.13	1.85	3.52	10.7	3.86	***0.00138***	***0.00105***
Femur-Rib	13.6	1.85	10.3	17.5	7.34	***< 0.001***	***< 0.001***
Random Factor	***Estimate***	***Std. Error***	***CI (2.5%)***	***CI (97.5%)***	***ICC (%)***	***p-value***	***Model Fit***
Individual	76.5	8.75	3.75	14.7	62.4	***0.0162***	R^2 M = 0.652
Age	0	0.00037	0.00002	0.000823	0	1	R^2 C = 0.869
Sex	0	0.00009	0.000054	0.000152	0	1	AIC = 197
Residual	46.1	6.79	4.33	8.96	37.6		BIC = 206
**% Bone Area Total Porosity Lambda = None**							
Fixed Factor	***Estimate***	***Std. Error***	***CI (2.5%)***	***CI (97.5%)***	***t***	***p-value***	***p-value MCMC***
Intercept	47	3.03	41.1	53.2	15.5	0.0828	0.979
Femur-Tibia	6.69	1.41	3.93	9.42	4.75	***< 0.001***	***< 0.001***
Femur-Rib	12.5	1.41	10	15.5	8.88	***< 0.001***	***< 0.001***
Random Factor	***Estimate***	***Std. Error***	***CI (2.5%)***	***CI (97.5%)***	***ICC (%)***	***p-value***	***Model Fit***
Individual	57	7.55	0	11.3	65.4	***0.00449***	R^2 M = 0.693
Age	0	0	0	11.2	0	1	R^2 C = 0.906
Sex	3.43	1.85	0	9.52	3.93	0.898	AIC = 186
Residual	26.8	5.18	3.3	6.78	30.7		BIC = 195
**% Bone Area Ct. Porosity Lambda = None**							
Fixed Factor	***Estimate***	***Std. Error***	***CI (2.5%)***	***CI (97.5%)***	***t***	***p-value***	***p-value MCMC***
Intercept	51.6	2.89	45.7	57.5	17.9	***< 0.001***	0.963
Femur-Tibia	5.89	1.56	2.84	8.91	3.78	***0.00163***	***0.00105***
Femur-Rib	12.2	1.56	9.45	15.5	7.86	***< 0.001***	***< 0.001***
Random Factor	***Estimate***	***Std. Error***	***CI (2.5%)***	***CI (97.5%)***	***ICC (%)***	***p-value***	***Model Fit***
Individual	64.2	8.01	0	12	66.3	***0.00667***	R^2 M = 0.647
Age	0	0	0	11.6	0	1	R^2 C = 0.881
Sex	0	0	0	8.79	0	1	AIC = 190
Residual	32.7	5.72	3.64	7.48	33.7		BIC = 199
**% Bone Area Tb. Porosity Lambda = None**							
Fixed Factor	***Estimate***	***Std. Error***	***CI (2.5%)***	***CI (97.5%)***	***t***	***p-value***	***p-value MCMC***
Intercept	51	2.94	44.9	57	17.3	***< 0.001***	0.966
Femur-Tibia	8.1	1.52	5.12	11	5.32	***< 0.001***	***< 0.001***
Femur-Rib	13.9	1.52	11.2	17.1	9.14	***< 0.001***	***< 0.001***
Random Factor	***Estimate***	***Std. Error***	***CI (2.5%)***	***CI (97.5%)***	***ICC (%)***	***p-value***	***Model Fit***
Individual	67.5	8.21	0	12.3	68.3	***0.0063***	R^2 M = 0.723
Age	0	0	0	11.8	0	1	R^2 C = 0.912
Sex	0	0	0	8.97	0	1	AIC = 190
Residual	31.2	5.59	3.56	7.33	31.7		BIC = 199

**Parabolic Index (Y) Lambda = None**							
Fixed Factor	***Estimate***	***Std. Error***	***CI (2.5%)***	***CI (97.5%)***	***t***	***p-value***	***p-value MCMC***
Intercept	0.213	0.00549	0.202	0.224	38.8	***< 0.001***	0.978
Femur-Tibia	0.0108	0.00777	-0.0044	0.0259	1.39	0.177	0.205
Femur-Rib	-0.00924	0.00777	-0.0231	0.00711	-1.19	0.246	0.272
Random Factor	***Estimate***	***Std. Error***	***CI (2.5%)***	***CI (97.5%)***	***ICC (%)***	***p-value***	***Model Fit***
Individual	0	0	0	0.0167	0	1	R^2 M = 0.0799
Age	0	0	0	0.0184	0	1	R^2 C = 0.0799
Sex	0	0	0	0.0185	0	1	AIC = -79.8
Residual	0.00082	0.0286	0.0182	0.0352	100	NA	BIC = - 70.7

SUPPLEMENTAL TABLE 5: Post-Hoc Analysis for Cross-Sectional Geometry

**Total Area (mm^2)**			
Comparison	***p-value***	***Cohen's D***	***Effect Size***
Femur > Tibia	***< 0.001***	3.08	Large
Femur > Rib	***< 0.001***	11	Large
Tibia > Rib	***< 0.001***	7.89	Large
**Endosteal Area (mm^2)**			
Comparison	***p-value***	***Cohen's D***	***Effect Size***
Femur > Tibia	***< 0.001***	2.47	Large
Femur > Rib	***< 0.001***	5.33	Large
Tibia > Rib	***< 0.001***	2.86	Large
**Cortical Area (mm^2)**			
Comparison	***p-value***	***Cohen's D***	***Effect Size***
Femur > Tibia	***0.00415***	1.8	Large
Femur > Rib	***< 0.001***	9.5	Large
Tibia > Rib	***< 0.001***	7.71	Large
**% Endosteal Area**			
Comparison	***p-value***	***Cohen's D***	***Effect Size***
Femur > Tibia	0.142	0.947	Large
Femur < Rib	***< 0.001***	4.1	Large
Tibia < Rib	***< 0.001***	5.05	Large
**% Cortical Area**			
Comparison	***p-value***	***Cohen's D***	***Effect Size***
Femur < Tibia	0.142	0.947	Large
Femur > Rib	***< 0.001***	4.1	Large
Tibia > Rib	***< 0.001***	5.05	Large
**% Bone Area Total Porosity**			
Comparison	***p-value***	***Cohen's D***	***Effect Size***
Femur < Tibia	0.072	1.13	Large
Femur > Rib	***< 0.001***	5	Large
Tibia > Rib	***< 0.001***	6.13	Large
**% Bone Area Ct. Porosity**			
Comparison	***p-value***	***Cohen's D***	***Effect Size***
Femur < Tibia	0.077	1.11	Large
Femur > Rib	***< 0.001***	4.2	Large
Tibia > Rib	***< 0.001***	5.31	Large
**% Bone Area Tb. Porosity**			
Comparison	***p-value***	***Cohen's D***	***Effect Size***
Femur < Tibia	0.101	1.04	Large
Femur > Rib	***< 0.001***	5.39	Large
Tibia > Rib	***< 0.001***	6.43	Large

SUPPLEMENTAL TABLE 6: Linear Mixed Model for Intraskeletal Comparisons

**Percent Porosity (%) Lambda = 0.075**							
Fixed Factor	***Estimate***	***Std. Error***	***CI (2.5%)***	***CI (97.5%)***	***t***	***p-value***	***p-value MCMC***
Intercept	1.16	0.00894	1.14	1.18	130	***< 0.001***	0.946
Femur-Tibia	-0.00548	0.00867	-0.0215	0.0105	-0.632	0.531	0.536
Femur-Rib	7.74E-05	0.00867	-0.0174	0.0176	0.00892	0.993	0.976
Ct.-Tb.	-0.00719	0.00613	-0.02	0.00447	-1.17	0.248	0.25
Femur-Tibia : Ct.-Tb.	0.0285	0.00867	0.00855	0.0476	3.29	***0.00211***	***0.0014***
Femur-Rib : Ct.-Tb.	0.0167	0.00867	0.00122	0.033	1.92	0.0616	0.0621
Random Factor	***Estimate***	***Std. Error***	***CI (2.5%)***	***CI (97.5%)***	***ICC (%)***	***p-value***	***Model Fit***
Individual	0.00038	0.0195	0	0.0331	15.8	0.529	R^2 M = 0.32
Age	0	0	0	0.0355	0	1	R^2 C = 0.428
Sex	0	0	0	0.0273	0	1	AIC = -114
Residual	0.00203	0.045	0.0341	0.0536	84.2		BIC = - 93.7
**Pore Density (1/mm²) Lambda = 0.025**							
Fixed Factor	***Estimate***	***Std. Error***	***CI (2.5%)***	***CI (97.5%)***	***t***	***p-value***	***p-value MCMC***
Intercept	1.01	0.0032	1.01	1.02	316	***0.00479***	0.942456
Femur-Tibia	-0.00617	0.00158	-0.00922	-0.00318	-3.91	***< 0.001***	***< 0.001***
Femur-Rib	-0.00828	0.00158	-0.0112	-0.00541	-5.25	***< 0.001***	***< 0.001***
Ct.-Tb.	0.0465	0.00111	0.0443	0.0486	41.8	***< 0.001***	***< 0.001***
Femur-Tibia : Ct.-Tb.	0.00643	0.00158	0.0034	0.00941	4.08	***< 0.001***	***< 0.001***
Femur-Rib : Ct.-Tb.	0.00874	0.00158	0.00568	0.0117	5.55	***< 0.001***	***< 0.001***
Random Factor	***Estimate***	***Std. Error***	***CI (2.5%)***	***CI (97.5%)***	***ICC (%)***	***p-value***	***Model Fit***
Individual	0	0	0	0.00759	0	1	R^2 M = 0.952
Age	0.00005	0.00722	0	0.0122	41.7	0.207	R^2 C = 0.974
Sex	0	0.00215	0	0.0106	0	0.871	AIC = -270
Residual	0.00007	0.00819	0.00632	0.0096	58.3		BIC = - 251
**Mean Area (μm²) Lambda = -0.15**							
Fixed Factor	***Estimate***	***Std. Error***	***CI (2.5%)***	***CI (97.5%)***	***t***	***p-value***	***p-value MCMC***
Intercept	-0.214	0.00428	-0.223	-0.205	-49.9	***< 0.001***	0.994
Femur-Tibia	0.00839	0.00355	0.00183	0.0149	2.36	***0.023***	***0.02***
Femur-Rib	0.0116	0.00355	0.00444	0.0188	3.26	***0.00223***	***0.00316***
Ct.-Tb.	-0.0556	0.00251	-0.0609	-0.0508	-22.1	***< 0.001***	***< 0.001***
Femur-Tibia : Ct.-Tb.	0.00364	0.00355	-0.00453	0.0114	1.03	0.311	0.3
Femur-Rib : Ct.-Tb.	-0.0028	0.00355	-0.00913	0.00387	-0.788	0.435	0.436
Random Factor	***Estimate***	***Std. Error***	***CI (2.5%)***	***CI (97.5%)***	***ICC (%)***	***p-value***	***Model Fit***
Individual	0	0	0	0.0128	0	0.999	R^2 M = 0.886
Age	0.00009	0.00971	0	0.0176	20.9	0.488	R^2 C = 0.91
Sex	0	0	1E-08	8.96E-06	0	1	AIC = -198
Residual	0.00034	0.0184	0.0142	0.0222	79.1		BIC = - 178

**Mean Perimeter (µm) Lambda = -0.325**							
Fixed Factor	***Estimate***	***Std. Error***	***CI (2.5%)***	***CI (97.5%)***	***t***	***p-value***	***p-value MCMC***
Intercept	-0.125	0.00257	-0.131	-0.12	-48.5	***< 0.001***	0.982
Femur-Tibia	0.00538	0.00242	0.000908	0.00983	2.22	***0.032***	***0.0256***
Femur-Rib	0.00892	0.00242	0.00405	0.0138	3.69	***< 0.001***	***< 0.001***
Ct.-Tb.	-0.0366	0.00171	-0.0402	-0.0334	-21.4	***< 0.001***	***< 0.001***
Femur-Tibia : Ct.-Tb.	0.00166	0.00242	-0.00391	0.00698	0.686	0.497	0.499
Femur-Rib : Ct.-Tb.	-0.0026	0.00242	-0.00691	0.00195	-1.07	0.29	0.289
Random Factor	***Estimate***	***Std. Error***	***CI (2.5%)***	***CI (97.5%)***	***ICC (%)***	***p-value***	***Model Fit***
Individual	0.00002	0.00417	0	0.00888	10.5	0.675	R^2 M = 0.887
Age	0.00001	0.00366	0	0.01	5.26	0.815	R^2 C = 0.905
Sex	0	0	0	0.00734	0	1	AIC = -236
Residual	0.00016	0.0126	0.00955	0.0149	84.2		BIC = - 216
**Mean Circularity Lambda = None**							
Fixed Factor	***Estimate***	***Std. Error***	***CI (2.5%)***	***CI (97.5%)***	***t***	***p-value***	***p-value MCMC***
Intercept	0.653	0.0116	0.629	0.674	56.4	***< 0.001***	0.982
Femur-Tibia	-0.00548	0.00964	-0.023	0.0143	-0.569	0.573	0.581
Femur-Rib	-0.0355	0.00964	-0.0536	-0.0158	-3.68	***< 0.001***	***< 0.001***
Ct.-Tb.	0.0514	0.00682	0.0379	0.0658	7.55	***< 0.001***	***< 0.001***
Femur-Tibia : Ct.-Tb.	-0.00501	0.00964	-0.0228	0.0139	-0.519	0.606	0.607
Femur-Rib : Ct.-Tb.	0.0136	0.00964	-0.00608	0.0311	1.41	0.165	0.158
Random Factor	***Estimate***	***Std. Error***	***CI (2.5%)***	***CI (97.5%)***	***ICC (%)***	***p-value***	***Model Fit***
Individual	0.00038	0.0196	0	0.0425	11.8	0.586	R^2 M = 0.540
Age	0.00034	0.0185	0	0.0503	10.5	0.766	R^2 C = 0.643
Sex	0	0	0	1.02E-05	0	1	AIC = -102
Residual	0.00251	0.0501	0.0398	0.0616	77.7		BIC = - 81.7
**Mean Max Feret Diameter (µm) Lambda = -0.3**							
Fixed Factor	***Estimate***	***Std. Error***	***CI (2.5%)***	***CI (97.5%)***	***t***	***p-value***	***p-value MCMC***
Intercept	-0.2	0.00388	-0.209	-0.193	-51.6	***< 0.001***	0.989
Femur-Tibia	0.00719	0.00352	0.000691	0.0137	2.04	***0.0477***	***0.0407***
Femur-Rib	0.0126	0.00352	0.00551	0.0197	3.58	***< 0.001***	***0.0014***
Ct.-Tb.	-0.0517	0.00249	-0.0569	-0.047	-20.8	***< 0.001***	***< 0.001***
Femur-Tibia : Ct.-Tb.	0.00267	0.00352	-0.00544	0.0104	0.759	0.452	0.441
Femur-Rib : Ct.-Tb.	-0.00484	0.00352	-0.0111	0.00177	-1.37	0.177	0.177
Random Factor	***Estimate***	***Std. Error***	***CI (2.5%)***	***CI (97.5%)***	***ICC (%)***	***p-value***	***Model Fit***
Individual	0.00002	0.00409	0	0.0126	5	0.834	R^2 M = 0.879
Age	0.00005	0.00735	0	0.0151	12.5	0.641	R^2 C = 0.900
Sex	0	0	0	0.0111	0	1	AIC = -200
Residual	0.00033	0.0183	0.0139	0.0219	82.5		BIC = - 180

**Mean Min Feret Diameter (µm)** **Lambda = -0.425**							
Fixed Factor	***Estimate***	***Std. Error***	***CI (2.5%)***	***CI (97.5%)***	***t***	***p-value***	***p-value MCMC***
Intercept	-0.133	0.00352	-0.141	-0.127	-37.8	***< 0.001***	0.978
Femur-Tibia	0.00656	0.00281	0.00137	0.0117	2.33	***0.0248***	***0.0214***
Femur-Rib	0.00939	0.00281	0.00373	0.0151	3.34	***0.00181***	***0.00246***
Ct.-Tb.	-0.0462	0.00199	-0.0504	-0.0424	-23.3	***< 0.001***	***< 0.001***
Femur-Tibia : Ct.-Tb.	0.00347	0.00281	-0.00301	0.00964	1.23	0.225	0.215
Femur-Rib : Ct.-Tb.	-0.00185	0.00281	-0.00686	0.00342	-0.659	0.514	0.514
Random Factor	***Estimate***	***Std. Error***	***CI (2.5%)***	***CI (97.5%)***	***ICC (%)***	***p-value***	***Model Fit***
Individual	0.00001	0.00238	0	0.011	3.57	0.922	R^2 M = 0.892
Age	0.00006	0.00783	0	0.0145	21.4	0.603	R^2 C = 0.918
Sex	0	0	5E-09	4.74E-07	0	1	AIC = -220
Residual	0.00021	0.0146	0.0112	0.0176	75		BIC = - 200
**Mean Aspect Ratio** **Lambda = -2.225**							
Fixed Factor	***Estimate***	***Std. Error***	***CI (2.5%)***	***CI (97.5%)***	***t***	***p-value***	***p-value MCMC***
Intercept	-0.253	0.0121	-0.28	-0.229	-20.9	0.0621	0.944
Femur-Tibia	0.000943	0.00989	-0.0173	0.0191	0.0953	0.925	0.921
Femur-Rib	0.0213	0.00989	0.00142	0.0414	2.16	***0.0371***	***0.0418***
Ct.-Tb.	-0.0123	0.00699	-0.0269	0.00104	-1.75	0.087	0.0832
Femur-Tibia : Ct.-Tb.	0.00461	0.00989	-0.0182	0.0263	0.466	0.644	0.645
Femur-Rib : Ct.-Tb.	-0.0215	0.00989	-0.0391	-0.00291	-2.17	***0.0359***	***0.0288***
Random Factor	***Estimate***	***Std. Error***	***CI (2.5%)***	***CI (97.5%)***	***ICC (%)***	***p-value***	***Model Fit***
Individual	0.00004	0.00601	0	0.0339	1.2	0.938	R^2 M = 0.182
Age	0.00062	0.0248	0	0.0469	18.6	0.341	R^2 C = 0.349
Sex	0.00003	0.00519	0	0.0404	0.901	0.953	AIC = -100
Residual	0.00264	0.0514	0.0393	0.0618	79.3		BIC = - 80.2
**Mean Roundness†** **Lambda = 2.875**							
Fixed Factor	***Estimate***	***Std. Error***	***CI (2.5%)***	***CI (97.5%)***	***t***	***p-value***	***p-value MCMC***
Intercept	0.257	0.0117	0.23	0.279	21.9	***0.048***	0.915
Femur-Tibia	-0.00777	0.00867	-0.0238	0.00819	-0.896	0.376	0.382
Femur-Rib	-0.0168	0.00867	-0.0342	0.000769	-1.94	0.0599	0.0625
Ct.-Tb.	0.0204	0.00613	0.00755	0.0321	3.33	***0.00189***	***0.0014***
Femur-Tibia : Ct.-Tb.	-0.00727	0.00867	-0.0272	0.0118	-0.838	0.407	0.407
Femur-Rib : Ct.-Tb.	0.0174	0.00867	0.00192	0.0337	2	0.0518	0.0505
Random Factor	Estimate	Std. Error	CI (2.5%)	CI (97.5%)	ICC (%)	p-value	***Model Fit***
Individual	0.00003	0.00508	0	0.0291	1.16	0.941	R^2 M = 0.259
Age	0.00044	0.021	0	0.0402	17.1	0.354	R^2 C = 0.416
Sex	0.00008	0.00867	0	0.0414	3.1	0.839	AIC = -113
Residual	0.00203	0.045	0.0344	0.0541	78.7		BIC = - 92.9

**Mean Solidity Lambda = None**							
Fixed Factor	***Estimate***	***Std. Error***	***CI (2.5%)***	***CI (97.5%)***	***t***	***p-value***	***p-value MCMC***
Intercept	0.874	0.00488	0.865	0.884	179	***< 0.001***	0.96
Femur-Tibia	-0.00062	0.00459	-0.00896	0.0088	-0.135	0.893	0.892
Femur-Rib	-0.00989	0.00459	-0.0185	-0.00052	-2.16	***0.0369***	***0.0379***
Ct.-Tb.	0.00296	0.00324	-0.0035	0.00978	0.913	0.366	0.355
Femur-Tibia : Ct.-Tb.	-0.00115	0.00459	-0.00962	0.00783	-0.251	0.803	0.794
Femur-Rib : Ct.-Tb.	0.0033	0.00459	-0.00608	0.0116	0.719	0.476	0.471
Random Factor	***Estimate***	***Std. Error***	***CI (2.5%)***	***CI (97.5%)***	***ICC (%)***	***p-value***	***Model Fit***
Individual	0	0	0	0.0151	0	1	R^2 M = 0.113
Age	0.0001	0.0102	0	0.0202	14.9	0.331	R^2 C = 0.250
Sex	0	0	6E-09	1.71E-06	0	1	AIC = -175
Residual	0.00057	0.0238	0.0189	0.0291	85.1		BIC = - 155

† Variable residuals remain not normally distributed after transformation; use Bayesian (MCMC) values

SUPPLEMENTAL TABLE 7: Post-Hoc Analysis for Intraskeletal Comparisons

**Percent Porosity (%)**					
Factor	***Contrast***	***Comparison***	***p-value***	***Cohen's D***	***Effect Size***
Pore Type | Bone	Ct.	Femur > Tibia	0.769	0.139	Small
Pore Type | Bone	Ct.	Femur > Rib	***0.00518***	1.39	Large
Pore Type | Bone	Ct.	Tibia > Rib	***0.0111***	1.25	Large
Pore Type | Bone	Tb.	Femur < Tibia	0.418	0.386	Small
Pore Type | Bone	Tb.	Femur < Rib	***< 0.001***	1.88	Large
Pore Type | Bone	Tb.	Tibia < Rib	***0.00297***	1.49	Large
Bone | Pore Type	Femur	Ct. > Tb.	0.0515	0.946	Large
Bone | Pore Type	Tibia	Ct. > Tb.	0.377	0.421	Small
Bone | Pore Type	Rib	Ct. < Tb.	***< 0.001***	2.33	Large
**Pore Density ($1/mm^2$)**					
Factor	***Contrast***	***Comparison***	***p-value***	***Cohen's D***	***Effect Size***
Bone		Femur > Tibia	0.721	0.258	Medium
Bone		Femur < Rib	***< 0.001***	2.52	Large
Bone		Tibia < Rib	***< 0.001***	2.78	Large
Pore Type		Ct. > Tb.	***< 0.001***	11.4	Large
Pore Type | Bone	Ct.	Femur < Tibia	0.958	0.0248	Small
Pore Type | Bone	Ct.	Femur > Rib	0.797	0.122	Small
Pore Type | Bone	Ct.	Tibia > Rib	0.757	0.147	Small
Pore Type | Bone	Tb.	Femur > Tibia	0.259	0.54	Medium
Pore Type | Bone	Tb.	Femur < Rib	***< 0.001***	5.16	Large
Pore Type | Bone	Tb.	Tibia < Rib	***< 0.001***	5.7	Large
Bone | Pore Type	Femur	Ct. > Tb.	***< 0.001***	12.9	Large
Bone | Pore Type	Tibia	Ct. > Tb.	***< 0.001***	13.5	Large
Bone | Pore Type	Rib	Ct. > Tb.	***< 0.001***	7.66	Large
**Mean Area (μm^2)**					
Factor	***Contrast***	***Comparison***	***p-value***	***Cohen's D***	***Effect Size***
Bone		Femur < Tibia	0.862	0.173	Small
Bone		Femur > Rib	***< 0.001***	1.54	Large
Bone		Tibia > Rib	***< 0.001***	1.71	Large
Pore Type		Ct. < Tb.	***< 0.001***	6.03	Large
Pore Type | Bone	Ct.	Femur > Tibia	0.711	0.176	Small
Pore Type | Bone	Ct.	Femur > Rib	***< 0.001***	1.78	Large
Pore Type | Bone	Ct.	Tibia > Rib	***0.00152***	1.61	Large
Pore Type | Bone	Tb.	Femur < Tibia	0.274	0.523	Medium
Pore Type | Bone	Tb.	Femur > Rib	***0.00902***	1.29	Large
Pore Type | Bone	Tb.	Tibia > Rib	***< 0.001***	1.82	Large
Bone | Pore Type	Femur	Ct. < Tb.	***< 0.001***	5.63	Large
Bone | Pore Type	Tibia	Ct. < Tb.	***< 0.001***	6.33	Large

Mean Area (µm^2) *Continued*					
Factor	***Factor***	***Factor***	***Factor***	***Factor***	***Factor***
Bone | Pore Type	Rib	Ct. < Tb.	***< 0.001***	6.12	Large
**Mean Perimeter (µm)**					
Factor	***Contrast***	***Comparison***	***p-value***	***Cohen's D***	***Effect Size***
Bone		Femur < Tibia	0.677	0.282	Small
Bone		Femur > Rib	***< 0.001***	1.56	Large
Bone		Tibia > Rib	***< 0.001***	1.85	Large
Pore Type		Ct. < Tb.	***< 0.001***	5.82	Large
Pore Type | Bone	Ct.	Femur > Tibia	0.906	0.0563	Small
Pore Type | Bone	Ct.	Femur > Rib	***0.00137***	1.62	Large
Pore Type | Bone	Ct.	Tibia > Rib	***0.00192***	1.57	Large
Pore Type | Bone	Tb.	Femur < Tibia	0.195	0.621	Medium
Pore Type | Bone	Tb.	Femur > Rib	***0.00271***	1.51	Large
Pore Type | Bone	Tb.	Tibia > Rib	***< 0.001***	2.13	Large
Bone | Pore Type	Femur	Ct. < Tb.	***< 0.001***	5.56	Large
Bone | Pore Type	Tibia	Ct. < Tb.	***< 0.001***	6.24	Large
Bone | Pore Type	Rib	Ct. < Tb.	***< 0.001***	5.67	Large
**Mean Circularity**					
Factor	***Contrast***	***Comparison***	***p-value***	***Cohen's D***	***Effect Size***
Bone		Femur > Tibia	0.183	0.599	Medium
Bone		Femur < Rib	***0.0218***	0.928	Large
Bone		Tibia < Rib	***< 0.001***	1.53	Large
Pore Type		Ct. > Tb.	***< 0.001***	2.05	Large
Pore Type | Bone	Ct.	Femur > Tibia	0.632	0.227	Small
Pore Type | Bone	Ct.	Femur < Rib	0.077	0.856	Large
Pore Type | Bone	Ct.	Tibia < Rib	***0.0269***	1.08	Large
Pore Type | Bone	Tb.	Femur > Tibia	***0.046***	0.971	Large
Pore Type | Bone	Tb.	Femur < Rib	***0.0402***	1	Large
Pore Type | Bone	Tb.	Tibia < Rib	***< 0.001***	1.97	Large
Bone | Pore Type	Femur	Ct. > Tb.	***< 0.001***	1.85	Large
Bone | Pore Type	Tibia	Ct. > Tb.	***< 0.001***	2.6	Large
Bone | Pore Type	Rib	Ct. > Tb.	***< 0.001***	1.71	Large
**Mean Max Feret Diameter (µm)**					
Factor	***Contrast***	***Comparison***	***p-value***	***Cohen's D***	***Effect Size***
Bone		Femur < Tibia	0.651	0.296	Small
Bone		Femur > Rib	***< 0.001***	1.47	Large
Bone		Tibia > Rib	***< 0.001***	1.77	Large
Pore Type		Ct. < Tb.	***< 0.001***	5.65	Large
Pore Type | Bone	Ct.	Femur > Tibia	0.809	0.115	Small
Pore Type | Bone	Ct.	Femur > Rib	***0.00279***	1.5	Large

**Mean Max Feret Diameter (µm)** ***Continued***					
Factor	***Factor***	***Factor***	***Factor***	***Factor***	***Factor***
Pore Type | Bone	Ct.	Tibia > Rib	***0.00538***	1.39	Large
Pore Type | Bone	Tb.	Femur < Tibia	0.142	0.706	Medium
Pore Type | Bone	Tb.	Femur > Rib	***0.00383***	1.45	Large
Pore Type | Bone	Tb.	Tibia > Rib	***< 0.001***	2.15	Large
Bone | Pore Type	Femur	Ct. < Tb.	***< 0.001***	5.36	Large
Bone | Pore Type	Tibia	Ct. < Tb.	***< 0.001***	6.18	Large
Bone | Pore Type	Rib	Ct. < Tb.	***< 0.001***	5.41	Large
**Mean Min Feret Diameter (µm)**					
Factor	***Contrast***	***Comparison***	***p-value***	***Cohen's D***	***Effect Size***
Bone		Femur < Tibia	0.83	0.194	Small
Bone		Femur > Rib	***< 0.001***	1.54	Large
Bone		Tibia > Rib	***< 0.001***	1.74	Large
Pore Type		Ct. < Tb.	***< 0.001***	6.33	Large
Pore Type | Bone	Ct.	Femur > Tibia	0.72	0.17	Small
Pore Type | Bone	Ct.	Femur > Rib	***< 0.001***	1.89	Large
Pore Type | Bone	Ct.	Tibia > Rib	***< 0.001***	1.72	Large
Pore Type | Bone	Tb.	Femur < Tibia	0.243	0.558	Medium
Pore Type | Bone	Tb.	Femur > Rib	***0.0154***	1.19	Large
Pore Type | Bone	Tb.	Tibia > Rib	***< 0.001***	1.75	Large
Bone | Pore Type	Femur	Ct. < Tb.	***< 0.001***	5.86	Large
Bone | Pore Type	Tibia	Ct. < Tb.	***< 0.001***	6.58	Large
Bone | Pore Type	Rib	Ct. < Tb.	***< 0.001***	6.55	Large
**Mean Aspect Ratio**					
Factor	***Contrast***	***Comparison***	***p-value***	***Cohen's D***	***Effect Size***
Bone		Femur < Tibia	0.466	0.397	Small
Bone		Femur > Rib	0.374	0.452	Small
Bone		Tibia > Rib	***0.0387***	0.849	Large
Pore Type		Ct. < Tb.	0.087	0.478	Small
Pore Type | Bone	Ct.	Femur > Tibia	0.815	0.111	Small
Pore Type | Bone	Ct.	Femur > Rib	0.654	0.213	Small
Pore Type | Bone	Ct.	Tibia > Rib	0.829	0.102	Small
Pore Type | Bone	Tb.	Femur < Tibia	0.0622	0.904	Large
Pore Type | Bone	Tb.	Femur > Rib	0.151	0.69	Medium
Pore Type | Bone	Tb.	Tibia > Rib	***0.00161***	1.59	Large
Bone | Pore Type	Femur	Ct. < Tb.	0.531	0.298	Small
Bone | Pore Type	Tibia	Ct. < Tb.	***0.00812***	1.31	Large
Bone | Pore Type	Rib	Ct. > Tb.	0.706	0.179	Small

<table>
<tr><td colspan="6">Mean Roundness</td></tr>
<tr><th>Factor</th><th>Contrast</th><th>Comparison</th><th>p-value</th><th>Cohen's D</th><th>Effect Size</th></tr>
<tr><td>Bone</td><td></td><td>Femur > Tibia</td><td>0.821</td><td>0.2</td><td>Small</td></tr>
<tr><td>Bone</td><td></td><td>Femur < Rib</td><td>0.0921</td><td>0.718</td><td>Medium</td></tr>
<tr><td>Bone</td><td></td><td>Tibia < Rib</td><td>0.0235</td><td>0.918</td><td>Large</td></tr>
<tr><td>Pore Type</td><td></td><td>Ct. > Tb.</td><td>< 0.001</td><td>0.905</td><td>Large</td></tr>
<tr><td>Pore Type | Bone</td><td>Ct.</td><td>Femur < Tibia</td><td>0.466</td><td>0.347</td><td>Small</td></tr>
<tr><td>Pore Type | Bone</td><td>Ct.</td><td>Femur < Rib</td><td>0.173</td><td>0.654</td><td>Medium</td></tr>
<tr><td>Pore Type | Bone</td><td>Ct.</td><td>Tibia < Rib</td><td>0.518</td><td>0.307</td><td>Small</td></tr>
<tr><td>Pore Type | Bone</td><td>Tb.</td><td>Femur > Tibia</td><td>0.121</td><td>0.747</td><td>Medium</td></tr>
<tr><td>Pore Type | Bone</td><td>Tb.</td><td>Femur < Rib</td><td>0.106</td><td>0.781</td><td>Medium</td></tr>
<tr><td>Pore Type | Bone</td><td>Tb.</td><td>Tibia < Rib</td><td>0.0024</td><td>1.53</td><td>Large</td></tr>
<tr><td>Bone | Pore Type</td><td>Femur</td><td>Ct. > Tb.</td><td>0.224</td><td>0.583</td><td>Medium</td></tr>
<tr><td>Bone | Pore Type</td><td>Tibia</td><td>Ct. > Tb.</td><td>< 0.001</td><td>1.68</td><td>Large</td></tr>
<tr><td>Bone | Pore Type</td><td>Rib</td><td>Ct. > Tb.</td><td>0.339</td><td>0.456</td><td>Small</td></tr>
<tr><td colspan="6">Mean Solidity</td></tr>
<tr><th>Factor</th><th>Contrast</th><th>Comparison</th><th>p-value</th><th>Cohen's D</th><th>Effect Size</th></tr>
<tr><td>Bone</td><td></td><td>Femur > Tibia</td><td>0.48</td><td>0.389</td><td>Small</td></tr>
<tr><td>Bone</td><td></td><td>Femur < Rib</td><td>0.35</td><td>0.467</td><td>Small</td></tr>
<tr><td>Bone</td><td></td><td>Tibia < Rib</td><td>0.0368</td><td>0.856</td><td>Large</td></tr>
<tr><td>Pore Type | Bone</td><td>Ct.</td><td>Femur > Tibia</td><td>0.67</td><td>0.202</td><td>Small</td></tr>
<tr><td>Pore Type | Bone</td><td>Ct.</td><td>Femur < Rib</td><td>0.373</td><td>0.425</td><td>Small</td></tr>
<tr><td>Pore Type | Bone</td><td>Ct.</td><td>Tibia < Rib</td><td>0.191</td><td>0.627</td><td>Medium</td></tr>
<tr><td>Pore Type | Bone</td><td>Tb.</td><td>Femur > Tibia</td><td>0.229</td><td>0.576</td><td>Medium</td></tr>
<tr><td>Pore Type | Bone</td><td>Tb.</td><td>Femur < Rib</td><td>0.287</td><td>0.508</td><td>Medium</td></tr>
<tr><td>Pore Type | Bone</td><td>Tb.</td><td>Tibia < Rib</td><td>0.0268</td><td>1.08</td><td>Large</td></tr>
</table>

SUPPLEMENTAL TABLE 8: Linear Mixed Model for Regional Comparisons: Femur

**Percent Porosity (%) Lambda = 0.3**							
Fixed Factor	***Estimate***	***Std. Error***	***CI (2.5%)***	***CI (97.5%)***	***t***	***p-value***	***p-value MCMC***
Intercept	1.83	0.0833	1.66	1.99	21.9	***< 0.001***	0.975
A-L	0.0354	0.0686	-0.0977	0.163	0.517	0.607	0.624
A-M	-0.106	0.0686	-0.25	0.0314	-1.55	0.126	0.128
A-P	-0.0808	0.0686	-0.217	0.0427	-1.18	0.244	0.255
Ct.-Tb.	0.0954	0.0396	0.00911	0.17	2.41	***0.0193***	***0.0182***
A-L : Ct.-Tb.	-0.0572	0.0686	-0.198	0.0891	-0.834	0.408	0.392
A-M : Ct.-Tb.	-0.003	0.0686	-0.147	0.127	-0.0437	0.965	0.996
A-P : Ct.-Tb.	0.00998	0.0686	-0.126	0.151	0.145	0.885	0.887
Random Factor	***Estimate***	***Std. Error***	***CI (2.5%)***	***CI (97.5%)***	***ICC (%)***	***p-value***	***Model Fit***
Individual	0.0484	0.22	0	0.356	30	***0.0125***	R^2 M = 0.117
Age	0	0	0	0.344	0	1	R^2 C = 0.382
Sex	0	0.00001	9E-08	2.32E-05	0	1	AIC = 197
Residual	0.113	0.336	0.269	0.401	70		BIC = 134
**Pore Density (1/mm^2) † Lambda = -0.3**							
Fixed Factor	***Estimate***	***Std. Error***	***CI (2.5%)***	***CI (97.5%)***	***t***	***p-value***	***p-value MCMC***
Intercept	-1.15	0.0501	-1.25	-1.05	-23	***< 0.001***	0.971
A-L	0.101	0.0459	0.00523	0.195	2.19	***0.0326***	***0.0354***
A-M	-0.0693	0.0459	-0.157	0.0271	-1.51	0.137	0.136
A-P	0.00977	0.0459	-0.0925	0.0947	0.213	0.832	0.835
Ct.-Tb.	0.649	0.0265	0.599	0.702	24.5	***< 0.001***	***< 0.001***
A-L : Ct.-Tb.	-0.0945	0.0459	-0.178	0.00131	-2.06	***0.0443***	0.0502
A-M : Ct.-Tb.	0.071	0.0459	-0.0107	0.153	1.55	0.128	0.132
A-P : Ct.-Tb.	-0.00467	0.0459	-0.0902	0.0841	-0.102	0.919	0.917
Random Factor	***Estimate***	***Std. Error***	***CI (2.5%)***	***CI (97.5%)***	***ICC (%)***	***p-value***	***Model Fit***
Individual	0.0162	0.127	0	0.2	24.3	0.0775	R^2 M = 0.867
Age	0	0	0	0.208	0	1	R^2 C = 0.899
Sex	0	0	0	0.165	0	1	AIC = 53.5
Residual	0.0506	0.225	0.184	0.261	75.7		BIC = 80.8
**Mean Area (μm^2) Lambda = -0.1**							
Fixed Factor	***Estimate***	***Std. Error***	***CI (2.5%)***	***CI (97.5%)***	***t***	***p-value***	***p-value MCMC***
Intercept	-0.35	0.00646	-0.364	-0.337	-54.2	***< 0.001***	0.988
A-L	-0.00097	0.00413	-0.00898	0.00673	-0.234	0.816	0.804
A-M	-0.00676	0.00413	-0.0154	0.00153	-1.64	0.107	0.102
A-P	-0.00539	0.00413	-0.0136	0.00204	-1.31	0.197	0.21
Ct.-Tb.	-0.057	0.00238	-0.0622	-0.0526	-23.9	***< 0.001***	***< 0.001***
A-L : Ct.-Tb.	-0.00154	0.00413	-0.01	0.00726	-0.373	0.711	0.699
A-M : Ct.-Tb.	-0.00149	0.00413	-0.0102	0.00632	-0.36	0.72	0.746
A-P : Ct.-Tb.	-0.00035	0.00413	-0.00854	0.00814	-0.0849	0.933	0.931

Mean Area (µm²) Lambda = -0.1 *Continued*							
Random Factor	***Estimate***	***Std. Error***	***CI (2.5%)***	***CI (97.5%)***	***ICC (%)***	***p-value***	***Model Fit***
Individual	0.00024	0.0155	0	0.0265	33.3	0.132	R^2 M = 0.824
Age	0.00007	0.00845	0	0.0277	9.72	0.887	R^2 C = 0.900
Sex	0	0	0	1.33E-06	0	1	AIC = -249
Residual	0.00041	0.0202	0.0161	0.0242	56.9		BIC = -222
**Mean Perimeter (µm) Lambda = -0.275**							
Fixed Factor	***Estimate***	***Std. Error***	***CI (2.5%)***	***CI (97.5%)***	***t***	***p-value***	***p-value MCMC***
Intercept	-0.167	0.00418	-0.175	-0.158	-39.8	***< 0.001***	0.986
A-L	-0.00147	0.00269	-0.00669	0.00355	-0.544	0.588	0.571
A-M	-0.00387	0.00269	-0.00953	0.00154	-1.44	0.156	0.155
A-P	-0.00345	0.00269	-0.00879	0.0014	-1.28	0.205	0.22
Ct.-Tb.	-0.0398	0.00155	-0.0432	-0.0369	-25.6	***< 0.001***	***< 0.001***
A-L : Ct.-Tb.	0.000445	0.00269	-0.00508	0.00619	0.165	0.869	0.884
A-M : Ct.-Tb.	-0.00082	0.00269	-0.00649	0.00427	-0.306	0.761	0.784
A-P : Ct.-Tb.	0.000106	0.00269	-0.00524	0.00565	0.0394	0.969	0.973
Random Factor	***Estimate***	***Std. Error***	***CI (2.5%)***	***CI (97.5%)***	***ICC (%)***	***p-value***	***Model Fit***
Individual	0.00011	0.0104	0	0.0168	36.7	0.0537	R^2 M = 0.843
Age	0.00002	0.00478	0	0.0179	6.67	0.87	R^2 C = 0.910
Sex	0	0	0	0.0131	0	1	AIC = -304
Residual	0.00017	0.0132	0.0105	0.0158	56.7		BIC = -277
**Mean Circularity Lambda = 3.475**							
Fixed Factor	***Estimate***	***Std. Error***	***CI (2.5%)***	***CI (97.5%)***	***t***	***p-value***	***p-value MCMC***
Intercept	0.223	0.0239	0.175	0.27	9.31	0.0849	0.949
A-L	0.0191	0.012	-0.00419	0.0415	1.59	0.117	0.114
A-M	0.00869	0.012	-0.0165	0.0328	0.724	0.472	0.479
A-P	-0.00035	0.012	-0.0242	0.0213	-0.0288	0.977	0.995
Ct.-Tb.	0.0575	0.00693	0.0424	0.0705	8.29	***< 0.001***	***< 0.001***
A-L : Ct.-Tb.	-0.0071	0.012	-0.0317	0.0185	-0.591	0.557	0.547
A-M : Ct.-Tb.	0.00747	0.012	-0.0178	0.0302	0.622	0.536	0.504
A-P : Ct.-Tb.	-0.00608	0.012	-0.0299	0.0186	-0.506	0.615	0.635
Random Factor	***Estimate***	***Std. Error***	***CI (2.5%)***	***CI (97.5%)***	***ICC (%)***	***p-value***	***Model Fit***
Individual	0.00123	0.0351	0	0.0603	21.2	0.169	R^2 M = 0.389
Age	0.00049	0.0222	0	0.0671	8.45	0.802	R^2 C = 0.636
Sex	0.00062	0.0248	0	0.0837	10.7	0.606	AIC = -115
Residual	0.00346	0.0588	0.0468	0.0701	59.7		BIC = -87.5
**Mean Max Feret Diameter (µm) Lambda = -0.3**							
Fixed Factor	***Estimate***	***Std. Error***	***CI (2.5%)***	***CI (97.5%)***	***t***	***p-value***	***p-value MCMC***
Intercept	-0.195	0.00538	-0.207	-0.184	-36.3	***< 0.001***	0.988
A-L	-0.00171	0.00333	-0.00817	0.0045	-0.513	0.61	0.595
A-M	-0.00353	0.00333	-0.0105	0.00316	-1.06	0.294	0.305

**Mean Max Feret Diameter (µm) Lambda = -0.3 *Continued***							
Fixed Factor	***Estimate***	***Std. Error***	***CI (2.5%)***	***CI (97.5%)***	***t***	***p-value***	***p-value MCMC***
A-P	-0.00426	0.00333	-0.0109	0.00174	-1.28	0.206	0.221
Ct.-Tb.	-0.0478	0.00192	-0.052	-0.0442	-24.9	***< 0.001***	***< 0.001***
A-L : Ct.-Tb.	0.000274	0.00333	-0.00656	0.00737	0.0823	0.935	0.948
A-M : Ct.-Tb.	-0.0019	0.00333	-0.00891	0.0044	-0.572	0.57	0.589
A-P : Ct.-Tb.	0.000306	0.00333	-0.0063	0.00715	0.0918	0.927	0.934
Random Factor	***Estimate***	***Std. Error***	***CI (2.5%)***	***CI (97.5%)***	***ICC (%)***	***p-value***	***Model Fit***
Individual	0.00017	0.0132	0	0.0221	35.4	0.0655	R^2 M = 0.829
Age	0.00004	0.00666	0	0.0232	8.33	0.865	R^2 C = 0.906
Sex	0	0	0	7.82E-06	0	1	AIC = -276
Residual	0.00027	0.0163	0.013	0.0195	56.2		BIC = -249
**Mean Min Feret Diameter (µm) Lambda = -0.175**							
Fixed Factor	***Estimate***	***Std. Error***	***CI (2.5%)***	***CI (97.5%)***	***t***	***p-value***	***p-value MCMC***
Intercept	-0.422	0.00679	-0.435	-0.409	-62.2	***< 0.001***	0.992
A-L	-0.00179	0.00379	-0.00916	0.00527	-0.473	0.638	0.624
A-M	-0.00478	0.00379	-0.0127	0.00283	-1.26	0.213	0.218
A-P	-0.00518	0.00379	-0.0127	0.00165	-1.37	0.177	0.193
Ct.-Tb.	-0.0595	0.00219	-0.0643	-0.0554	-27.2	***< 0.001***	***< 0.001***
A-L : Ct.-Tb.	0.000961	0.00379	-0.00682	0.00905	0.253	0.801	0.814
A-M : Ct.-Tb.	-0.00083	0.00379	-0.00881	0.00634	-0.22	0.827	0.855
A-P : Ct.-Tb.	-0.00058	0.00379	-0.0081	0.00722	-0.152	0.879	0.878
Random Factor	***Estimate***	***Std. Error***	***CI (2.5%)***	***CI (97.5%)***	***ICC (%)***	***p-value***	***Model Fit***
Individual	0.00011	0.0106	0	0.0229	16.2	0.29	R^2 M = 0.842
Age	0.00022	0.015	0	0.0288	32.4	0.624	R^2 C = 0.920
Sex	0	0.00001	1E-08	2.69E-05	0	1	AIC = -259
Residual	0.00035	0.0186	0.0148	0.0222	51.5		BIC = -232
**Mean Aspect Ratio Lambda = -3.525**							
Fixed Factor	***Estimate***	***Std. Error***	***CI (2.5%)***	***CI (97.5%)***	***t***	***p-value***	***p-value MCMC***
Intercept	-0.116	0.0108	-0.139	-0.0933	-10.8	0.0881	0.939
A-L	-0.0149	0.00835	-0.0311	0.000641	-1.79	0.0793	0.0835
A-M	0.00587	0.00835	-0.0117	0.0226	0.703	0.485	0.492
A-P	-0.00064	0.00835	-0.0172	0.0144	-0.0768	0.939	0.962
Ct.-Tb.	-0.00474	0.00482	-0.0153	0.00432	-0.984	0.329	0.339
A-L : Ct.-Tb.	0.00136	0.00835	-0.0158	0.0192	0.163	0.871	0.886
A-M : Ct.-Tb.	-0.00837	0.00835	-0.0259	0.00743	-1	0.32	0.335
A-P : Ct.-Tb.	0.00597	0.00835	-0.0106	0.0231	0.715	0.477	0.473
Random Factor	***Estimate***	***Std. Error***	***CI (2.5%)***	***CI (97.5%)***	***ICC (%)***	***p-value***	***Model Fit***
Individual	0.00031	0.0176	0	0.0315	14.6	0.206	R^2 M = 0.0617
Age	0.00004	0.00645	0	0.0326	1.89	0.896	R^2 C = 0.260
Sex	0.0001	0.00997	0	0.0363	4.72	0.722	AIC = -167

**Mean Aspect Ratio Lambda = -3.525** ***Continued***							
Random Factor	***Estimate***	***Std. Error***	***CI (2.5%)***	***CI (97.5%)***	***ICC (%)***	***p-value***	***Model Fit***
Residual	0.00167	0.0409	0.0326	0.0485	78.8		BIC = -139
**Mean Roundness Lambda = 4.825**							
Fixed Factor	***Estimate***	***Std. Error***	***CI (2.5%)***	***CI (97.5%)***	***t***	***p-value***	***p-value MCMC***
Intercept	0.0961	0.0112	0.0737	0.118	8.57	0.0923	0.921
A-L	0.0132	0.00608	0.00141	0.0245	2.17	***0.034***	***0.0337***
A-M	-0.00467	0.00608	-0.0174	0.00754	-0.768	0.446	0.445
A-P	0.000824	0.00608	-0.0112	0.0118	0.136	0.893	0.875
Ct.-Tb.	0.0101	0.00351	0.00242	0.0167	2.87	***0.0058***	***0.00526***
A-L : Ct.-Tb.	-0.00247	0.00608	-0.0149	0.0105	-0.406	0.686	0.676
A-M : Ct.-Tb.	0.00709	0.00608	-0.00569	0.0186	1.17	0.248	0.222
A-P : Ct.-Tb.	-0.00447	0.00608	-0.0165	0.00802	-0.736	0.465	0.491
Random Factor	***Estimate***	***Std. Error***	***CI (2.5%)***	***CI (97.5%)***	***ICC (%)***	***p-value***	***Model Fit***
Individual	0.00025	0.016	0	0.0279	18	0.121	R^2 M = 0.122
Age	0.00012	0.011	0	0.0315	8.63	0.677	R^2 C = 0.442
Sex	0.00013	0.0115	0	0.0396	9.35	0.617	AIC = -203
Residual	0.00089	0.0298	0.0237	0.0354	64		BIC = -176
**Mean Solidity Lambda = 9.875**							
Fixed Factor	***Estimate***	***Std. Error***	***CI (2.5%)***	***CI (97.5%)***	***t***	***p-value***	***p-value MCMC***
Intercept	0.272	0.0179	0.234	0.309	15.2	***< 0.001***	0.945
A-L	0.0193	0.0186	-0.0167	0.054	1.04	0.302	0.3
A-M	0.0122	0.0186	-0.0268	0.0495	0.656	0.515	0.522
A-P	-0.0123	0.0186	-0.0491	0.0212	-0.662	0.511	0.52
Ct.-Tb.	0.00437	0.0107	-0.019	0.0245	0.407	0.686	0.693
A-L : Ct.-Tb.	-0.00696	0.0186	-0.0451	0.0326	-0.375	0.709	0.698
A-M : Ct.-Tb.	-0.00288	0.0186	-0.042	0.0323	-0.155	0.877	0.9
A-P : Ct.-Tb.	-0.00046	0.0186	-0.0373	0.0377	-0.0247	0.98	0.973
Random Factor	***Estimate***	***Std. Error***	***CI (2.5%)***	***CI (97.5%)***	***ICC (%)***	***p-value***	***Model Fit***
Individual	0	0	0	0.055	0	1	R^2 M = 0.0318
Age	0.00162	0.0402	0	0.0701	16.4	0.459	R^2 C = 0.190
Sex	0	0	0	0.0591	0	1	AIC = -65.5
Residual	0.00828	0.091	0.0726	0.109	83.6		BIC = -38.2

† Variable residuals remain not normally distributed after transformation; use Bayesian (MCMC) values

SUPPLEMENTAL TABLE 9: Post-Hoc Analysis for Regional Comparisons: Femur

Percent Porosity (%)

Factor	*Contrast*	*Comparison*	*p-value*	*Cohen's D*	*Effect Size*
Pore Type		Ct. > Tb.	***0.0193***	0.568	Medium

Pore Density ($1/mm^2$)

Factor	*Contrast*	*Comparison*	*p-value*	*Cohen's D*	*Effect Size*
Region		Anterior > Lateral	0.118	0.755	Medium
Region		Anterior > Medial	0.622	0.404	Medium
Region		Anterior > Posterior	0.244	0.63	Medium
Region		Lateral < Medial	0.718	0.351	Medium
Region		Lateral < Posterior	0.982	0.125	Small
Region		Medial > Posterior	0.905	0.226	Medium
Pore Type		Ct. > Tb.	***< 0.001***	5.77	Large
Pore Type | Region	Ct.	Anterior > Lateral	0.967	0.0197	Small
Pore Type | Region	Ct.	Anterior > Medial	0.992	0.00479	Small
Pore Type | Region	Ct.	Anterior > Posterior	0.857	0.0853	Small
Pore Type | Region	Ct.	Lateral < Medial	0.975	0.0149	Small
Pore Type | Region	Ct.	Lateral > Posterior	0.89	0.0656	Small
Pore Type | Region	Ct.	Medial > Posterior	0.865	0.0805	Small
Pore Type | Region	Tb.	Anterior > Lateral	***0.00252***	1.49	Large
Pore Type | Region	Tb.	Anterior > Medial	0.0939	0.803	Large
Pore Type | Region	Tb.	Anterior > Posterior	***0.0156***	1.18	Large
Pore Type | Region	Tb.	Lateral < Medial	0.15	0.688	Medium
Pore Type | Region	Tb.	Lateral < Posterior	0.506	0.316	Medium
Pore Type | Region	Tb.	Medial > Posterior	0.433	0.372	Medium
Region | Pore Type	Anterior	Ct. > Tb.	***< 0.001***	4.93	Large
Region | Pore Type	Lateral	Ct. > Tb.	***< 0.001***	6.4	Large
Region | Pore Type	Medial	Ct. > Tb.	***< 0.001***	5.73	Large
Region | Pore Type	Posterior	Ct. > Tb.	***< 0.001***	6.02	Large

Mean Area (μm^2)

Factor	*Contrast*	*Comparison*	*p-value*	*Cohen's D*	*Effect Size*
Pore Type		Ct. < Tb.	***< 0.001***	5.64	Large

Mean Perimeter (μm)

Factor	*Contrast*	*Comparison*	*p-value*	*Cohen's D*	*Effect Size*
Pore Type		Ct. < Tb.	***< 0.001***	6.04	Large

Mean Circularity

Factor	*Contrast*	*Comparison*	*p-value*	*Cohen's D*	*Effect Size*
Pore Type		Ct. > Tb.	***< 0.001***	1.95	Large

Mean Max Feret Diameter (μm)

Factor	*Contrast*	*Comparison*	*p-value*	*Cohen's D*	*Effect Size*
Pore Type		Ct. < Tb.	***< 0.001***	5.86	Large

**Mean Min Feret Diameter (µm)**					
Factor	***Contrast***	***Comparison***	***p-value***	***Cohen's D***	***Effect Size***
Pore Type		Ct. < Tb.	***< 0.001***	6.41	Large
**Mean Roundness**					
Factor	***Contrast***	***Comparison***	***p-value***	***Cohen's D***	***Effect Size***
Region		Anterior > Lateral	0.284	0.601	Medium
Region		Anterior > Medial	0.600	0.416	Small
Region		Anterior > Posterior	0.116	0.758	Medium
Region		Lateral < Medial	0.945	0.184	Small
Region		Lateral > Posterior	0.965	0.158	Small
Region		Medial > Posterior	0.735	0.342	Small
Pore Type		Ct. > Tb.	***0.0058***	0.676	Medium

SUPPLEMENTAL TABLE 10: Linear Mixed Model for Regional Comparisons: Tibia

**Percent Porosity (%)† Lambda = 0.525**							
Fixed Factor	***Estimate***	***Std. Error***	***CI (2.5%)***	***CI (97.5%)***	***t***	***p-value***	***p-value MCMC***
Intercept	2.92	0.176	2.59	3.26	16.6	***< 0.001***	0.991
A-L	0.723	0.149	0.402	0.999	4.86	***< 0.001***	***< 0.001***
A-M	-0.605	0.149	-0.883	-0.32	-4.07	***< 0.001***	***< 0.001***
A-P	0.176	0.149	-0.139	0.473	1.18	0.243	0.25
Ct.-Tb.	0.0275	0.0858	-0.14	0.193	0.32	0.75	0.734
A-L : Ct.-Tb.	0.0401	0.149	-0.247	0.333	0.269	0.789	0.797
A-M : Ct.-Tb.	0.189	0.149	-0.0757	0.465	1.27	0.209	0.208
A-P : Ct.-Tb.	-0.383	0.149	-0.688	-0.0889	-2.57	***0.0128***	***0.0133***
Random Factor	***Estimate***	***Std. Error***	***CI (2.5%)***	***CI (97.5%)***	***ICC (%)***	***p-value***	***Model Fit***
Individual	0.213	0.461	0.102	0.794	28.6	0.24	R^2 M = 0.293
Age	0	0.00065	0.0000109	0.00141	0	1	R^2 C = 0.495
Sex	0	0.0003	0.0000419	0.000603	0	1	AIC = 205
Residual	0.531	0.728	0.586	0.861	71.4		BIC = 233
**Pore Density (1/mm²)† Lambda = -0.225**							
Fixed Factor	***Estimate***	***Std. Error***	***CI (2.5%)***	***CI (97.5%)***	***t***	***p-value***	***p-value MCMC***
Intercept	-1.13	0.071	-1.27	-0.992	-15.9	***0.0457***	0.927
A-L	-0.0386	0.0331	-0.101	0.0278	-1.17	0.248	0.24
A-M	-0.028	0.0331	-0.0889	0.0322	-0.846	0.401	0.417
A-P	0.00978	0.0331	-0.0594	0.0798	0.295	0.769	0.768
Ct.-Tb.	0.504	0.0191	0.47	0.541	26.4	***< 0.001***	0.000175
A-L : Ct.-Tb.	0.0451	0.0331	-0.017	0.115	1.36	0.178	0.167
A-M : Ct.-Tb.	0.0314	0.0331	-0.0308	0.102	0.949	0.347	0.354
A-P : Ct.-Tb.	-0.00493	0.0331	-0.0758	0.0597	-0.149	0.882	0.876
Random Factor	***Estimate***	***Std. Error***	***CI (2.5%)***	***CI (97.5%)***	***ICC (%)***	***p-value***	***Model Fit***
Individual	0	0.00056	0.000005	0.00121	0	1	R^2 M = 0.851
Age	6.79	2.61	0.497	4.42	29.7	0.235	R^2 C = 0.914
Sex	2.87	1.7	0	5.16	12.6	0.554	AIC = 14.1
Residual	13.2	3.63	2.92	4.27	57.7		BIC = 41.4
**Mean Area (μm²)† Lambda = -0.225**							
Fixed Factor	***Estimate***	***Std. Error***	***CI (2.5%)***	***CI (97.5%)***	***t***	***p-value***	***p-value MCMC***
Intercept	-0.1	0.00447	-0.109	-0.0914	-22.4	***< 0.001***	0.996
A-L	0.0133	0.00324	0.0072	0.02	4.11	***< 0.001***	***< 0.001***
A-M	-0.0137	0.00324	-0.0203	-0.00739	-4.23	***< 0.001***	***< 0.001***
A-P	0.000994	0.00324	-0.00573	0.00676	0.307	0.76	0.767
Ct.-Tb.	-0.0368	0.00187	-0.0409	-0.0333	-19.7	***< 0.001***	***< 0.001***
A-L : Ct.-Tb.	-0.00149	0.00324	-0.00806	0.00481	-0.459	0.648	0.64
A-M : Ct.-Tb.	0.00566	0.00324	-0.000877	0.0132	1.75	0.0864	0.0891
A-P : Ct.-Tb.	-0.00666	0.00324	-0.0132	-0.000189	-2.05	***0.0448***	0.0523

**Mean Area (µm^2)† Lambda = -0.225 *Continued***							
Random Factor	***Estimate***	***Std. Error***	***CI (2.5%)***	***CI (97.5%)***	***ICC (%)***	***p-value***	***Model Fit***
Individual	0.00002	0.00429	0	0.0133	5.26	0.726	R^2 M = 0.795
Age	0.00011	0.0107	0	0.0185	28.9	0.431	R^2 C = 0.865
Sex	0	0.00002	0.000000005	0.0084	0	1	AIC = -283
Residual	0.00025	0.0159	0.0127	0.0187	65.8		BIC = -256
**Mean Perimeter (µm)† Lambda = -0.5**							
Fixed Factor	***Estimate***	***Std. Error***	***CI (2.5%)***	***CI (97.5%)***	***t***	***p-value***	***p-value MCMC***
Intercept	-0.0406	0.00182	-0.0443	-0.0369	-22.3	***< 0.001***	0.994
A-L	0.00546	0.00151	0.0026	0.00855	3.61	***< 0.001***	***0.0014***
A-M	-0.00585	0.00151	-0.00892	-0.0029	-3.87	***< 0.001***	***0.00105***
A-P	0.000441	0.00151	-0.00269	0.00313	0.292	0.771	0.779
Ct.-Tb.	-0.0173	0.000873	-0.0192	-0.0157	-19.8	***< 0.001***	***< 0.001***
A-L : Ct.-Tb.	-0.00203	0.00151	-0.00509	0.000908	-1.34	0.186	0.193
A-M : Ct.-Tb.	0.00389	0.00151	0.000846	0.00741	2.58	***0.0127***	***0.0165***
A-P : Ct.-Tb.	-0.00303	0.00151	-0.00609	-0.0000156	-2	0.0498	0.0575
Random Factor	***Estimate***	***Std. Error***	***CI (2.5%)***	***CI (97.5%)***	***ICC (%)***	***p-value***	***Model Fit***
Individual	0.00001	0.0038	0	0.00711	14.3	0.311	R^2 M = 0.811
Age	0.00001	0.00265	0	0.00761	14.3	0.807	R^2 C = 0.864
Sex	0	0	0	0.00000351	0	1	AIC = -382
Residual	0.00005	0.00741	0.00591	0.00873	71.4		BIC = -355
**Mean Circularity† Lambda = 1.225**							
Fixed Factor	***Estimate***	***Std. Error***	***CI (2.5%)***	***CI (97.5%)***	***t***	***p-value***	***p-value MCMC***
Intercept	0.56	0.0107	0.536	0.582	52.4	***< 0.001***	0.996
A-L	-0.0248	0.0172	-0.0573	0.0104	-1.44	0.156	0.145
A-M	0.033	0.0172	-0.00201	0.0665	1.91	0.0608	0.0611
A-P	-0.0101	0.0172	-0.0458	0.0205	-0.586	0.561	0.562
Ct.-Tb.	0.0678	0.00994	0.0461	0.0865	6.82	***< 0.001***	***< 0.001***
A-L : Ct.-Tb.	0.0239	0.0172	-0.011	0.0573	1.39	0.171	0.168
A-M : Ct.-Tb.	-0.0326	0.0172	-0.0673	0.00747	-1.89	0.0635	0.0632
A-P : Ct.-Tb.	0.0218	0.0172	-0.013	0.0561	1.26	0.211	0.194
Random Factor	***Estimate***	***Std. Error***	***CI (2.5%)***	***CI (97.5%)***	***ICC (%)***	***p-value***	***Model Fit***
Individual	0.00014	0.0118	0	0.0379	1.93	0.781	R^2 M = 0.440
Age	0	0	0	0.034	0	1	R^2 C = 0.450
Sex	0	0	0	0.0328	0	1	AIC = -81
Residual	0.00712	0.0844	0.0676	0.0984	98.1		BIC = -53.7
**Mean Max Feret Diameter (µm)† Lambda = -0.525**							
Fixed Factor	***Estimate***	***Std. Error***	***CI (2.5%)***	***CI (97.5%)***	***t***	***p-value***	***p-value MCMC***
Intercept	-0.0603	0.00298	-0.0661	-0.054	-20.2	***< 0.001***	0.981
A-L	0.00793	0.00226	0.00366	0.0126	3.51	***< 0.001***	***0.0014***
A-M	-0.0089	0.00226	-0.0135	-0.00449	-3.93	***< 0.001***	***< 0.001***

**Mean Max Feret Diameter (µm)† Lambda = -0.525** ***Continued***							
Fixed Factor	***Estimate***	***Std. Error***	***CI (2.5%)***	***CI (97.5%)***	***t***	***p-value***	***p-value MCMC***
A-P	0.00081	0.00226	-0.00388	0.00483	0.358	0.722	0.731
Ct.-Tb.	-0.0259	0.00131	-0.0288	-0.0235	-19.8	***< 0.001***	***< 0.001***
A-L : Ct.-Tb.	-0.00295	0.00226	-0.00753	0.00145	-1.3	0.198	0.204
A-M : Ct.-Tb.	0.00614	0.00226	0.00158	0.0114	2.71	***0.00881***	***0.00772***
A-P : Ct.-Tb.	-0.0051	0.00226	-0.00968	-0.00059	-2.26	***0.028***	***0.0312***
Random Factor	***Estimate***	***Std. Error***	***CI (2.5%)***	***CI (97.5%)***	***ICC (%)***	***p-value***	***Model Fit***
Individual	0.00001	0.00367	0	0.00928	5.88	0.599	R^2 M = 0.803
Age	0.00004	0.00668	0	0.0122	23.5	0.458	R^2 C = 0.866
Sex	0	0	0	0.0096	0	1	AIC = -330
Residual	0.00012	0.0111	0.00886	0.013	70.6		BIC = -303
**Mean Min Feret Diameter (µm)† Lambda = -0.525**							
Fixed Factor	***Estimate***	***Std. Error***	***CI (2.5%)***	***CI (97.5%)***	***t***	***p-value***	***p-value MCMC***
Intercept	-0.0811	0.00385	-0.0889	-0.0728	-21	***< 0.001***	0.998
A-L	0.00978	0.0024	0.00525	0.0147	4.08	***< 0.001***	***< 0.001***
A-M	-0.0101	0.0024	-0.0149	-0.00539	-4.19	***< 0.001***	***< 0.001***
A-P	0.000443	0.0024	-0.00453	0.00471	0.184	0.854	0.858
Ct.-Tb.	-0.0343	0.00139	-0.0373	-0.0317	-24.7	***< 0.001***	***< 0.001***
A-L : Ct.-Tb.	-0.00286	0.0024	-0.00772	0.0018	-1.19	0.238	0.241
A-M : Ct.-Tb.	0.00597	0.0024	0.00113	0.0116	2.49	***0.0159***	***0.0193***
A-P : Ct.-Tb.	-0.00508	0.0024	-0.00993	-0.000297	-2.12	***0.0386***	***0.0442***
Random Factor	***Estimate***	***Std. Error***	***CI (2.5%)***	***CI (97.5%)***	***ICC (%)***	***p-value***	***Model Fit***
Individual	0.00003	0.00563	0	0.0124	12.5	0.376	R^2 M = 0.838
Age	0.00007	0.00858	0	0.0161	29.2	0.517	R^2 C = 0.908
Sex	0	0	0	0.0125	0	1	AIC = -319
Residual	0.00014	0.0118	0.00938	0.0138	58.3		BIC = -292
**Mean Aspect Ratio Lambda = None**							
Fixed Factor	***Estimate***	***Std. Error***	***CI (2.5%)***	***CI (97.5%)***	***t***	***p-value***	***p-value MCMC***
Intercept	2	0.106	1.78	2.2	18.9	***0.036***	0.921
A-L	0.0232	0.0657	-0.11	0.14	0.353	0.725	0.726
A-M	-0.0434	0.0657	-0.197	0.0869	-0.661	0.511	0.519
A-P	0.0161	0.0657	-0.124	0.14	0.245	0.807	0.814
Ct.-Tb.	-0.118	0.0379	-0.188	-0.0388	-3.12	***0.00284***	***0.00386***
A-L : Ct.-Tb.	-0.0485	0.0657	-0.181	0.0803	-0.739	0.463	0.452
A-M : Ct.-Tb.	0.0636	0.0657	-0.0721	0.201	0.968	0.337	0.333
A-P : Ct.-Tb.	-0.0789	0.0657	-0.204	0.0699	-1.2	0.235	0.236
Random Factor	***Estimate***	***Std. Error***	***CI (2.5%)***	***CI (97.5%)***	***ICC (%)***	***p-value***	***Model Fit***
Individual	0	0	0	0.166	0	1	R^2 M = 0.129
Age	0.00885	0.0941	0	0.2	6.85	0.325	R^2 C = 0.302
Sex	0.0168	0.13	0	0.345	13	0.3	AIC = 94.4

**Mean Aspect Ratio** **Lambda = None** ***Continued***							
Random Factor	***Estimate***	***Std. Error***	***CI (2.5%)***	***CI (97.5%)***	***ICC (%)***	***p-value***	***Model Fit***
Residual	0.104	0.322	0.259	0.369	80.1		BIC = 122
**Mean Roundness†** **Lambda = 2.325**							
Fixed Factor	***Estimate***	***Std. Error***	***CI (2.5%)***	***CI (97.5%)***	***t***	***p-value***	***p-value MCMC***
Intercept	0.316	0.0154	0.283	0.347	20.5	0.0531	0.932
A-L	0.00104	0.0158	-0.0288	0.0332	0.0657	0.948	0.974
A-M	0.0149	0.0158	-0.0172	0.0456	0.943	0.35	0.341
A-P	-0.011	0.0158	-0.0437	0.0171	-0.697	0.489	0.493
Ct.-Tb.	0.0345	0.00911	0.0146	0.0516	3.78	***< 0.001***	***0.00105***
A-L : Ct.-Tb.	0.00705	0.0158	-0.0249	0.0377	0.447	0.657	0.681
A-M : Ct.-Tb.	-0.0192	0.0158	-0.051	0.0175	-1.22	0.229	0.227
A-P : Ct.-Tb.	0.0261	0.0158	-0.00571	0.0576	1.66	0.103	0.102
Random Factor	***Estimate***	***Std. Error***	***CI (2.5%)***	***CI (97.5%)***	***ICC (%)***	***p-value***	***Model Fit***
Individual	0	0	0	0.0423	0	1	R^2 M = 0.192
Age	0.00074	0.0272	0	0.0503	10.9	0.196	R^2 C = 0.293
Sex	0.00011	0.0106	0	0.0488	1.61	0.866	AIC = -88.1
Residual	0.00597	0.0773	0.0617	0.0909	87.5		BIC = -60.7
**Mean Solidity** **Lambda = None**							
Fixed Factor	***Estimate***	***Std. Error***	***CI (2.5%)***	***CI (97.5%)***	***t***	***p-value***	***p-value MCMC***
Intercept	0.869	0.00601	0.857	0.882	144	***< 0.001***	0.979
A-L	-0.0114	0.00712	-0.0258	0.00122	-1.6	0.115	0.115
A-M	0.00851	0.00712	-0.00811	0.0226	1.2	0.237	0.228
A-P	-0.000101	0.00712	-0.0153	0.0133	-0.0142	0.989	0.98
Ct.-Tb.	0.00368	0.00411	-0.00387	0.0123	0.895	0.375	0.359
A-L : Ct.-Tb.	0.0104	0.00712	-0.00399	0.0243	1.46	0.151	0.164
A-M : Ct.-Tb.	-0.00781	0.00712	-0.0225	0.00705	-1.1	0.277	0.292
A-P : Ct.-Tb.	0.00171	0.00712	-0.0119	0.0178	0.241	0.811	0.82
Random Factor	***Estimate***	***Std. Error***	***CI (2.5%)***	***CI (97.5%)***	***ICC (%)***	***p-value***	***Model Fit***
Individual	0.00015	0.0122	0	0.0224	10.8	0.401	R^2 M = 0.0769
Age	0.00002	0.00458	0	0.0235	1.44	0.918	R^2 C = 0.189
Sex	0	0	0	0.021	0	1	AIC = -189
Residual	0.00122	0.0349	0.0281	0.0401	87.8		BIC = -162

† Variable residuals remain not normally distributed after transformation; use Bayesian (MCMC) values

SUPPLEMENTAL TABLE 11: Post-Hoc Analysis for Regional Comparisons: Tibia

**Percent Porosity (%)**					
Factor	***Contrast***	***Comparison***	***p-value***	***Cohen's D***	***Effect Size***
Region		Anterior > Lateral	***< 0.001***	1.82	Large
Region		Anterior > Medial	0.121	0.752	Medium
Region		Anterior > Posterior	***< 0.001***	1.4	Large
Region		Lateral < Medial	***0.0113***	1.07	Large
Region		Lateral < Posterior	0.579	0.427	Small
Region		Medial > Posterior	0.226	0.645	Medium
Pore Type | Region	Ct.	Anterior > Lateral	***0.00113***	1.62	Large
Pore Type | Region	Ct.	Anterior > Medial	***0.00654***	1.33	Large
Pore Type | Region	Ct.	Anterior > Posterior	***0.011***	1.24	Large
Pore Type | Region	Ct.	Lateral < Medial	0.545	0.287	Small
Pore Type | Region	Ct.	Lateral < Posterior	0.425	0.379	Small
Pore Type | Region	Ct.	Medial < Posterior	0.847	0.0917	Small
Pore Type | Region	Tb.	Anterior > Lateral	***< 0.001***	2.03	Large
Pore Type | Region	Tb.	Anterior > Medial	0.718	0.171	Small
Pore Type | Region	Tb.	Anterior > Posterior	***0.00172***	1.55	Large
Pore Type | Region	Tb.	Lateral < Medial	***< 0.001***	1.86	Large
Pore Type | Region	Tb.	Lateral < Posterior	0.318	0.475	Small
Pore Type | Region	Tb.	Medial > Posterior	***0.00491***	1.38	Large
Region | Pore Type	Anterior	Ct. > Tb.	0.696	0.185	Small
Region | Pore Type	Lateral	Ct. > Tb.	0.213	0.594	Medium
Region | Pore Type	Medial	Ct. < Tb.	***0.0432***	0.975	Large
Region | Pore Type	Posterior	Ct. > Tb.	0.296	0.497	Small
**Pore Density (1/µm^2)**					
Factor	***Contrast***	***Comparison***	***p-value***	***Cohen's D***	***Effect Size***
Pore Type		Ct. > Tb.	***< 0.001***	6.22	Large
**Mean Area (µm^2)**					
Factor	***Contrast***	***Comparison***	***p-value***	***Cohen's D***	***Effect Size***
Region		Anterior > Lateral	***< 0.001***	1.7	Large
Region		Anterior > Medial	0.104	0.776	Medium
Region		Anterior > Posterior	0.0519	0.877	Large
Region		Lateral < Medial	***0.0364***	0.926	Large
Region		Lateral < Posterior	0.0753	0.824	Large
Region		Medial > Posterior	0.99	0.101	Small
Pore Type		Ct. < Tb.	***< 0.001***	4.64	Large
Pore Type | Region	Ct.	Anterior > Lateral	***0.0103***	1.25	Large
Pore Type | Region	Ct.	Anterior > Medial	***0.023***	1.1	Large
Pore Type | Region	Ct.	Anterior > Posterior	0.189	0.627	Medium
Pore Type | Region	Ct.	Lateral < Medial	0.751	0.15	Small

**Mean Area (µm²) *Continued***					
Factor	***Contrast***	***Comparison***	***p-value***	***Cohen's D***	***Effect Size***
Pore Type | Region	Ct.	Lateral < Posterior	0.191	0.625	Medium
Pore Type | Region	Ct.	Medial < Posterior	0.319	0.474	Medium
Pore Type | Region	Tb.	Anterior > Lateral	***< 0.001***	2.15	Large
Pore Type | Region	Tb.	Anterior > Medial	0.343	0.451	Small
Pore Type | Region	Tb.	Anterior > Posterior	***0.0202***	1.13	Large
Pore Type | Region	Tb.	Lateral < Medial	***< 0.001***	1.7	Large
Pore Type | Region	Tb.	Lateral < Posterior	***0.034***	1.02	Large
Pore Type | Region	Tb.	Medial > Posterior	0.157	0.676	Medium
Region | Pore Type	Anterior	Ct. < Tb.	***< 0.001***	4.82	Large
Region | Pore Type	Lateral	Ct. < Tb.	***< 0.001***	3.92	Large
Region | Pore Type	Medial	Ct. < Tb.	***< 0.001***	5.48	Large
Region | Pore Type	Posterior	Ct. < Tb.	***< 0.001***	4.32	Large
**Mean Perimeter (µm)**					
Factor	***Contrast***	***Comparison***	***p-value***	***Cohen's D***	***Effect Size***
Region		Anterior > Lateral	***< 0.001***	1.53	Large
Region		Anterior > Medial	0.189	0.677	Medium
Region		Anterior > Posterior	0.127	0.744	Medium
Region		Lateral < Medial	0.0637	0.849	Large
Region		Lateral < Posterior	0.0999	0.782	Medium
Region		Medial > Posterior	0.997	0.0668	Small
Pore Type		Ct. < Tb.	***< 0.001***	4.68	Large
Pore Type | Region	Ct.	Anterior > Lateral	0.129	0.727	Medium
Pore Type | Region	Ct.	Anterior > Medial	0.0902	0.813	Large
Pore Type | Region	Ct.	Anterior > Posterior	0.509	0.313	Small
Pore Type | Region	Ct.	Lateral > Medial	0.856	0.0862	Small
Pore Type | Region	Ct.	Lateral < Posterior	0.384	0.413	Small
Pore Type | Region	Ct.	Medial < Posterior	0.294	0.5	Medium
Pore Type | Region	Tb.	Anterior > Lateral	***< 0.001***	2.33	Large
Pore Type | Region	Tb.	Anterior > Medial	0.256	0.541	Medium
Pore Type | Region	Tb.	Anterior > Posterior	***0.0157***	1.17	Large
Pore Type | Region	Tb.	Lateral < Medial	***< 0.001***	1.78	Large
Pore Type | Region	Tb.	Lateral < Posterior	***0.0179***	1.15	Large
Pore Type | Region	Tb.	Medial > Posterior	0.185	0.633	Medium
Region | Pore Type	Anterior	Ct. < Tb.	***< 0.001***	5.22	Large
Region | Pore Type	Lateral	Ct. < Tb.	***< 0.001***	3.63	Large
Region | Pore Type	Medial	Ct. < Tb.	***< 0.001***	5.5	Large
Region | Pore Type	Posterior	Ct. < Tb.	***< 0.001***	4.36	Large

**Mean Circularity**					
Factor	***Contrast***	***Comparison***	***p-value***	***Cohen's D***	***Effect Size***
Pore Type		Ct. > Tb.	***< 0.001***	1.61	Large
**Mean Max Feret Diameter (µm)**					
Factor	***Contrast***	***Comparison***	***p-value***	***Cohen's D***	***Effect Size***
Region		Anterior > Lateral	***< 0.001***	1.52	Large
Region		Anterior > Medial	0.228	0.642	Medium
Region		Anterior > Posterior	0.165	0.701	Medium
Region		Lateral < Medial	0.0524	0.876	Large
Region		Lateral < Posterior	0.0789	0.818	Large
Region		Medial > Posterior	0.998	0.0586	Small
Pore Type		Ct. < Tb.	***< 0.001***	4.68	Large
Pore Type | Region	Ct.	Anterior > Lateral	0.144	0.699	Medium
Pore Type | Region	Ct.	Anterior > Medial	0.0812	0.837	Large
Pore Type | Region	Ct.	Anterior > Posterior	0.579	0.263	Small
Pore Type | Region	Ct.	Lateral > Medial	0.77	0.138	Small
Pore Type | Region	Ct.	Lateral < Posterior	0.359	0.436	Small
Pore Type | Region	Ct.	Medial < Posterior	0.229	0.574	Medium
Pore Type | Region	Tb.	Anterior > Lateral	***< 0.001***	2.34	Large
Pore Type | Region	Tb.	Anterior > Medial	0.346	0.448	Small
Pore Type | Region	Tb.	Anterior > Posterior	***0.019***	1.14	Large
Pore Type | Region	Tb.	Lateral < Medial	***< 0.001***	1.89	Large
Pore Type | Region	Tb.	Lateral < Posterior	***0.0137***	1.2	Large
Pore Type | Region	Tb.	Medial > Posterior	0.148	0.691	Medium
Region | Pore Type	Anterior	Ct. < Tb.	***< 0.001***	5.21	Large
Region | Pore Type	Lateral	Ct. < Tb.	***< 0.001***	3.57	Large
Region | Pore Type	Medial	Ct. < Tb.	***< 0.001***	5.6	Large
Region | Pore Type	Posterior	Ct. < Tb.	***< 0.001***	4.33	Large
**Mean Min Feret Diameter (µm)**					
Factor	***Contrast***	***Comparison***	***p-value***	***Cohen's D***	***Effect Size***
Region		Anterior > Lateral	***< 0.001***	1.69	Large
Region		Anterior > Medial	0.0922	0.794	Medium
Region		Anterior > Posterior	0.0653	0.845	Large
Region		Lateral < Medial	***0.0461***	0.894	Large
Region		Lateral < Posterior	0.0663	0.843	Large
Region		Medial > Posterior	0.999	0.0509	Small
Pore Type		Ct. < Tb.	***< 0.001***	5.83	Large
Pore Type | Region	Ct.	Anterior > Lateral	0.0517	0.937	Large
Pore Type | Region	Ct.	Anterior > Medial	***0.0415***	0.983	Large
Pore Type | Region	Ct.	Anterior > Posterior	0.361	0.434	Small
Pore Type | Region	Ct.	Lateral > Medial	0.922	0.0461	Small

**Mean Min Feret Diameter (µm)** ***Continued***					
Factor	***Factor***	***Factor***	***Factor***	***Factor***	***Factor***
Pore Type | Region	Ct.	Lateral < Posterior	0.29	0.503	Medium
Pore Type | Region	Ct.	Medial < Posterior	0.249	0.55	Medium
Pore Type | Region	Tb.	Anterior > Lateral	***< 0.001***	2.44	Large
Pore Type | Region	Tb.	Anterior > Medial	0.205	0.605	Medium
Pore Type | Region	Tb.	Anterior > Posterior	***0.01***	1.26	Large
Pore Type | Region	Tb.	Lateral < Medial	***< 0.001***	1.83	Large
Pore Type | Region	Tb.	Lateral < Posterior	***0.015***	1.18	Large
Pore Type | Region	Tb.	Medial > Posterior	0.173	0.651	Medium
Region | Pore Type	Anterior	Ct. < Tb.	***< 0.001***	6.32	Large
Region | Pore Type	Lateral	Ct. < Tb.	***< 0.001***	4.82	Large
Region | Pore Type	Medial	Ct. < Tb.	***< 0.001***	6.7	Large
Region | Pore Type	Posterior	Ct. < Tb.	***< 0.001***	5.5	Large
**Mean Aspect Ratio**					
Factor	***Contrast***	***Comparison***	***p-value***	***Cohen's D***	***Effect Size***
Pore Type		Ct. < Tb.	***0.00288***	0.735	Medium
**Mean Roundness**					
Factor	***Contrast***	***Comparison***	***p-value***	***Cohen's D***	***Effect Size***
Pore Type		Ct. > Tb.	***< 0.001***	0.892	Large

SUPPLEMENTAL TABLE 12: Linear Mixed Model for Regional Comparisons: Rib

**Percent Porosity (%) Lambda = None**							
Fixed Factor	***Estimate***	***Std. Error***	***CI (2.5%)***	***CI (97.5%)***	***t***	***p-value***	***p-value MCMC***
Intercept	12.2	2.3	7.69	17.4	5.33	***< 0.001***	0.966
Region	4.87	1.16	2.61	7.28	4.21	***< 0.001***	***< 0.001***
Type	-6.96	1.16	-9.25	-4.74	-6	***< 0.001***	***< 0.001***
Region : Type	-5.48	1.16	-7.82	-3.19	-4.73	***< 0.001***	***< 0.001***
Random Factor	***Estimate***	***Std. Error***	***CI (2.5%)***	***CI (97.5%)***	***ICC (%)***	***p-value***	***Model Fit***
Individual	35.4	5.95	0	8.83	42.2	0.0647	R^2 M = 0.557
Age	0	0	0	9.01	0	1	R^2 C = 0.744
Sex	0	0	0	6.6	0	1	AIC = 256
Residual	48.3	6.95	5.04	8.6	57.8		BIC = 269
**Pore Density (1/mm^2) Lambda = None**							
Fixed Factor	***Estimate***	***Std. Error***	***CI (2.5%)***	***CI (97.5%)***	***t***	***p-value***	***p-value MCMC***
Intercept	5.43	0.311	4.84	6	17.5	***< 0.001***	0.951
Region	-0.803	0.215	-1.25	-0.353	-3.73	***< 0.001***	***0.0014***
Type	4.52	0.215	4.14	4.95	21	***< 0.001***	***< 0.001***
Region : Type	-0.841	0.215	-1.29	-0.447	-3.91	***< 0.001***	***< 0.001***
Random Factor	***Estimate***	***Std. Error***	***CI (2.5%)***	***CI (97.5%)***	***ICC (%)***	***p-value***	***Model Fit***
Individual	0	0.00005	0.0000044	0.0000809	0	1	R^2 M = 0.916
Age	0.389	0.624	0	1.22	19	0.439	R^2 C = 0.932
Sex	0	0	0	0.955	0	1	AIC = 142
Residual	1.66	1.29	0.924	1.61	81		BIC = 155
**Mean Area (μm^2) Lambda = -0.175**							
Fixed Factor	***Estimate***	***Std. Error***	***CI (2.5%)***	***CI (97.5%)***	***t***	***p-value***	***p-value MCMC***
Intercept	-0.192	0.00844	-0.211	-0.175	-22.8	***< 0.001***	0.964
Region	0.0117	0.00308	0.00499	0.018	3.81	***< 0.001***	***0.00105***
Type	-0.0473	0.00308	-0.0533	-0.0416	-15.4	***< 0.001***	***< 0.001***
Region : Type	-0.00894	0.00308	-0.0149	-0.00299	-2.91	***0.00775***	***0.0109***
Random Factor	***Estimate***	***Std. Error***	***CI (2.5%)***	***CI (97.5%)***	***ICC (%)***	***p-value***	***Model Fit***
Individual	0.00021	0.0144	0	0.0299	24.7	0.382	R^2 M = 0.748
Age	0.0003	0.0174	0	0.0348	35.3	0.805	R^2 C = 0.899
Sex	0	0	0	0.0246	0	1	AIC = -119
Residual	0.00034	0.0185	0.0131	0.0229	40		BIC = -107
**Mean Perimeter (μm) Lambda = -0.575**							
Fixed Factor	***Estimate***	***Std. Error***	***CI (2.5%)***	***CI (97.5%)***	***t***	***p-value***	***p-value MCMC***
Intercept	-0.0332	0.00233	-0.0384	-0.0282	-14.3	***< 0.001***	0.976
Region	0.00262	0.000797	0.000879	0.00424	3.29	***0.00299***	***0.00351***
Type	-0.013	0.000797	-0.0146	-0.0115	-16.3	***< 0.001***	***< 0.001***
Region : Type	-0.00147	0.000797	-0.00301	0.0000715	-1.84	0.077	0.0818

**Mean Perimeter (µm) Lambda = -0.575 *Continued***							
Random Factor	***Estimate***	***Std. Error***	***CI (2.5%)***	***CI (97.5%)***	***ICC (%)***	***p-value***	***Model Fit***
Individual	0	0	0	0.00522	0	1	R^2 M = 0.751
Age	0.00004	0.00616	0	0.0101	66.7	0.246	R^2 C = 0.906
Sex	0	0	0	0.00678	0	1	AIC = -206
Residual	0.00002	0.00478	0.00338	0.00591	33.3		BIC = -193
**Mean Circularity Lambda = None**							
Fixed Factor	***Estimate***	***Std. Error***	***CI (2.5%)***	***CI (97.5%)***	***t***	***p-value***	***p-value MCMC***
Intercept	0.689	0.02	0.65	0.73	34.5	***< 0.001***	0.993
Region	-0.00229	0.0113	-0.0234	0.0186	-0.203	0.841	0.824
Type	0.0463	0.0113	0.0257	0.0689	4.1	***< 0.001***	***< 0.001***
Region : Type	-0.00992	0.0113	-0.0314	0.0129	-0.879	0.388	0.396
Random Factor	***Estimate***	***Std. Error***	***CI (2.5%)***	***CI (97.5%)***	***ICC (%)***	***p-value***	***Model Fit***
Individual	0	0	0.00000059	0.00000594	0	1	R^2 M = 0.256
Age	0.00212	0.0461	0	0.084	31.6	0.497	R^2 C = 0.492
Sex	0	0	0.0000005	0.00000636	0	1	AIC = -43.4
Residual	0.00458	0.0677	0.0479	0.0849	68.4		BIC = -30.8
**Mean Max Feret Diameter (µm) Lambda = -0.6**							
Fixed Factor	***Estimate***	***Std. Error***	***CI (2.5%)***	***CI (97.5%)***	***t***	***p-value***	***p-value MCMC***
Intercept	-0.0531	0.00369	-0.0611	-0.045	-14.4	***< 0.001***	0.994
Region	0.00451	0.00131	0.00164	0.00717	3.44	***0.00206***	***0.00175***
Type	-0.0206	0.00131	-0.0231	-0.0181	-15.7	***< 0.001***	***< 0.001***
Region : Type	-0.0022	0.00131	-0.00472	0.000337	-1.68	0.106	0.11
Random Factor	***Estimate***	***Std. Error***	***CI (2.5%)***	***CI (97.5%)***	***ICC (%)***	***p-value***	***Model Fit***
Individual	0	0	0.000000057	0.000000661	0	1	R^2 M = 0.747
Age	0.00009	0.00971	0.00342	0.0164	60	0.243	R^2 C = 0.900
Sex	0	0	0.000000008	0.00000186	0	1	AIC = -175
Residual	0.00006	0.00786	0.00566	0.00966	40		BIC = -162
**Mean Min Feret Diameter (µm) Lambda = -0.55**							
Fixed Factor	***Estimate***	***Std. Error***	***CI (2.5%)***	***CI (97.5%)***	***t***	***p-value***	***p-value MCMC***
Intercept	-0.0912	0.00508	-0.102	-0.08	-17.9	***< 0.001***	0.966
Region	0.00603	0.00187	0.00194	0.00982	3.23	***0.00348***	***0.00386***
Type	-0.033	0.00187	-0.0367	-0.0296	-17.7	***< 0.001***	***< 0.001***
Region : Type	-0.00595	0.00187	-0.00955	-0.00234	-3.19	***0.00383***	***0.00632***
Random Factor	***Estimate***	***Std. Error***	***CI (2.5%)***	***CI (97.5%)***	***ICC (%)***	***p-value***	***Model Fit***
Individual	0	0	0	0.0118	0	1	R^2 M = 0.798
Age	0.00018	0.0133	0	0.0218	58.1	0.26	R^2 C = 0.916
Sex	0	0	0	0.0148	0	1	AIC = -153
Residual	0.00013	0.0112	0.00792	0.0138	41.9		BIC = -140

**Mean Aspect Ratio Lambda = -2.9**							
Fixed Factor	***Estimate***	***Std. Error***	***CI (2.5%)***	***CI (97.5%)***	***t***	***p-value***	***p-value MCMC***
Intercept	-0.192	0.0136	-0.219	-0.165	-14.1	***< 0.001***	0.994
Region	-0.00109	0.0136	-0.0308	0.0266	-0.08	0.937	0.937
Type	0.00185	0.0136	-0.0248	0.0268	0.136	0.893	0.897
Region : Type	0.023	0.0136	-0.00325	0.0493	1.69	0.101	0.103
Random Factor	***Estimate***	***Std. Error***	***CI (2.5%)***	***CI (97.5%)***	***ICC (%)***	***p-value***	***Model Fit***
Individual	0	0	0	0.0412	0	1	R^2 M = 0.0761
Age	0	0	0	0.0481	0	1	R^2 C = 0.0761
Sex	0	0	0	0.0383	0	1	AIC = -39.3
Residual	0.00665	0.0815	0.0576	0.0982	100		BIC = -26.6
**Mean Roundness Lambda = 4.475**							
Fixed Factor	***Estimate***	***Std. Error***	***CI (2.5%)***	***CI (97.5%)***	***t***	***p-value***	***p-value MCMC***
Intercept	0.142	0.0111	0.117	0.163	12.8	***< 0.001***	0.982
Region	-0.000165	0.00817	-0.018	0.0165	-0.0201	0.984	0.981
Type	0.00866	0.00817	-0.00735	0.0237	1.06	0.299	0.284
Region : Type	-0.0136	0.00817	-0.0293	0.00224	-1.66	0.11	0.11
Random Factor	***Estimate***	***Std. Error***	***CI (2.5%)***	***CI (97.5%)***	***ICC (%)***	***p-value***	***Model Fit***
Individual	0	0	0	0.0321	0	1	R^2 M = 0.0858
Age	0.00044	0.0209	0	0.0393	15.5	0.66	R^2 C = 0.226
Sex	0	0	0	0.0319	0	1	AIC = -67.7
Residual	0.0024	0.049	0.0347	0.0602	84.5		BIC = -55.1
**Mean Solidity Lambda = 6.25**							
Fixed Factor	***Estimate***	***Std. Error***	***CI (2.5%)***	***CI (97.5%)***	***t***	***p-value***	***p-value MCMC***
Intercept	0.475	0.0298	0.412	0.531	15.9	0.0653	0.952
Region	0.014	0.0189	-0.0273	0.0525	0.742	0.465	0.485
Type	-0.00829	0.0189	-0.0453	0.0265	-0.438	0.665	0.672
Region : Type	-0.0252	0.0189	-0.0616	0.0114	-1.33	0.195	0.191
Random Factor	***Estimate***	***Std. Error***	***CI (2.5%)***	***CI (97.5%)***	***ICC (%)***	***p-value***	***Model Fit***
Individual	0	0	0.00000136	0.00000663	0	1	R^2 M = 0.0557
Age	0.00236	0.0486	0	0.0932	15.1	0.47	R^2 C = 0.224
Sex	0.00043	0.0206	0	0.0908	2.75	0.865	AIC = -13.7
Residual	0.0129	0.113	0.0814	0.14	82.2		BIC = -1.08

SUPPLEMENTAL TABLE 13: Post-Hoc Analysis for Regional Comparisons: Rib

**Percent Porosity (%)**					
Factor	***Contrast***	***Comparison***	***p-value***	***Cohen's D***	***Effect Size***
Region		Cutaneous > Pleural	***< 0.001***	1.4	Large
Pore Type		Ct. < Tb.	***< 0.001***	2	Large
Pore Type | Region	Ct.	Cutaneous < Pleural	0.717	0.173	Small
Pore Type | Region	Tb.	Cutaneous > Pleural	***< 0.001***	2.98	Large
Region | Pore Type	Cutaneous	Ct. < Tb.	***< 0.001***	3.58	Large
Region | Pore Type	Pleural	Ct. < Tb.	0.375	0.426	Small
**Pore Density (1/mm^2)**					
Region		Cutaneous < Pleural	***0.00103***	1.24	Large
Pore Type		Cortical > Trabecularized	***< 0.001***	7.01	Large
Pore Type | Region	Ct.	Cutaneous < Pleural	***< 0.001***	2.55	Large
Pore Type | Region	Tb.	Cutaneous > Pleural	0.9	0.0597	Small
Region | Pore Type	Cutaneous	Cortical > Trabecularized	***< 0.001***	5.7	Large
Region | Pore Type	Pleural	Cortical > Trabecularized	***< 0.001***	8.31	Large
**Mean Area (µm^2)**					
Factor	***Contrast***	***Comparison***	***p-value***	***Cohen's D***	***Effect Size***
Region		Cutaneous > Pleural	***< 0.001***	1.27	Large
Pore Type		Ct. < Tb.	***< 0.001***	5.12	Large
Pore Type | Region	Ct.	Cutaneous > Pleural	0.53	0.301	Small
Pore Type | Region	Tb.	Cutaneous > Pleural	***< 0.001***	2.24	Large
Region | Pore Type	Cutaneous	Ct. < Tb.	***< 0.001***	6.09	Large
Region | Pore Type	Pleural	Ct. < Tb.	***< 0.001***	4.15	Large
**Mean Perimeter (µm)**					
Factor	***Contrast***	***Comparison***	***p-value***	***Cohen's D***	***Effect Size***
Region		Cutaneous > Pleural	***0.00309***	1.1	Large
Pore Type		Ct. < Tb.	***< 0.001***	5.44	Large
**Mean Circularity**					
Factor	***Contrast***	***Comparison***	***p-value***	***Cohen's D***	***Effect Size***
Pore Type		Ct. > Tb.	***< 0.001***	1.37	Large
**Mean Max Feret Diameter (µm)**					
Factor	***Contrast***	***Comparison***	***p-value***	***Cohen's D***	***Effect Size***
Region		Cutaneous > Pleural	***0.00213***	1.15	Large
Pore Type		Ct. < Tb.	***< 0.001***	5.23	Large
**Mean Min Feret Diameter (µm)**					
Factor	***Contrast***	***Comparison***	***p-value***	***Cohen's D***	***Effect Size***
Region		Cutaneous > Pleural	***0.00358***	1.08	Large
Pore Type		Ct. < Tb.	***< 0.001***	5.89	Large
Pore Type | Region	Ct.	Cutaneous > Pleural	0.978	0.013	Small
Pore Type | Region	Tb.	Cutaneous > Pleural	***< 0.001***	2.14	Large

**Mean Min Feret Diameter (µm)** ***Continued***					
Factor	***Contrast***	***Comparison***	***p-value***	***Cohen's D***	***Effect Size***
Region | Pore Type	Cutaneous	Ct. < Tb.	***< 0.001***	6.96	Large
Region | Pore Type	Pleural	Ct. < Tb.	***< 0.001***	4.83	Large
